# Frequency and hydrogen bonding of nucleobase homopairs in small molecule crystals

**DOI:** 10.1093/nar/gkaa629

**Published:** 2020-07-29

**Authors:** Małgorzata Katarzyna Cabaj, Paulina Maria Dominiak

**Affiliations:** Biological and Chemical Research Centre, Department of Chemistry, University of Warsaw, ul. Żwirki i Wigury 101, 02-089 Warszawa, Poland; Biological and Chemical Research Centre, Department of Chemistry, University of Warsaw, ul. Żwirki i Wigury 101, 02-089 Warszawa, Poland

## Abstract

We used the high resolution and accuracy of the Cambridge Structural Database (CSD) to provide detailed information regarding base pairing interactions of selected nucleobases. We searched for base pairs in which nucleobases interact with each other through two or more hydrogen bonds and form more or less planar structures. The investigated compounds were either free forms or derivatives of adenine, guanine, hypoxanthine, thymine, uracil and cytosine. We divided our findings into categories including types of pairs, protonation patterns and whether they are formed by free bases or substituted ones. We found base pair types that are exclusive to small molecule crystal structures, some that can be found only in RNA containing crystal structures and many that are native to both environments. With a few exceptions, nucleobase protonation generally followed a standard pattern governed by p*K*_a_ values. The lengths of hydrogen bonds did not depend on whether the nucleobases forming a base pair were charged or not. The reasons why particular nucleobases formed base pairs in a certain way varied significantly.

## INTRODUCTION

Nucleobases are small organic molecules which are the building blocks of important biological macromolecules like DNA or RNA. Nucleotides are linked into long chains by phosphodiester covalent bonds, twisted and held together by various types of interactions. Both the sequence of nucleobases in the chain, and their mutual orientation contribute to the correct performance of crucial tasks like protein synthesis, catalysis of biological reactions or gene information transmission (1). It is not surprising that these molecules caught the interest of many researchers, which contributed to numerous discoveries being made in this area of research.

**Figure 1.**
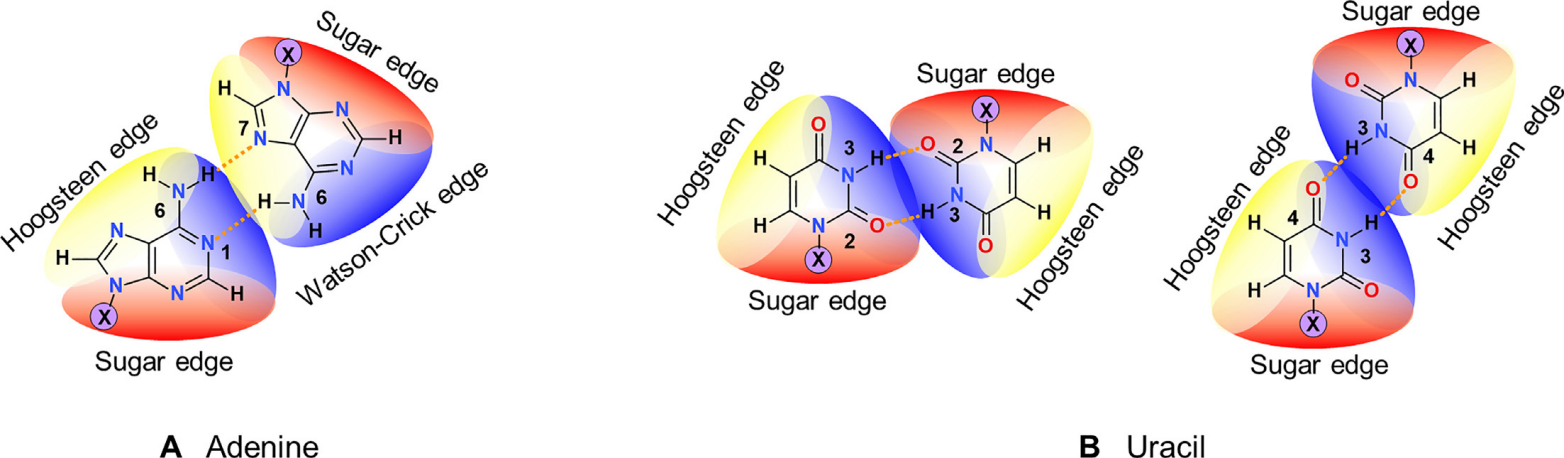
Examples of homo-base pairs of adenine (**A**) or uracil (**B**), with the naming of the edges.

At the time of the discovery of the structure of DNA, it was suggested that possible arrangements between nucleobases are limited to only a few options (2). These options are now called canonical base pairs, or Watson–Crick base pairs. Since then it has been demonstrated that the canonical base pairs were but a small fraction of possible pairs that nucleobases can form (3–6), each of which are necessary for folded molecular assemblies to function properly (7). The edges through which the nucleobases interact to form the canonical base pairs were called the Watson–Crick edges, while the other two were named Hoogsteen and Sugar edges (Figure 1, Supplementary Figure S.1 in Supplementary Information SI1). Their ability to form new types of base pairs significantly expands the possible number of orientations and interactions nucleobases may form. These interactions are not limited to hydrogen bonds between nitrogen and oxygen atoms, there are numerous possible base pairs that form hydrogen bonds with carbon atoms as proton donors (8), or have water mediated hydrogen bonds. Many pairs in RNA are assembled by nucleobases forming one base-base hydrogen bond, and one base-ribose hydrogen bond. Stacking interactions between nucleobases are another important type of interaction that allows large biological molecules like DNA or RNA to maintain their proper functions (9).

The planar, electron rich ring(s) of nucleobases enable them to adopt various tautomeric forms mediated by changes in the positions of hydrogen atoms. Moreover, nucleobases are capable of having non-neutral charge, which is usually a positive charge resulting from protonation of their lone electron pair(s). Nucleobases can have different p*K*_a_ values when they are part of a nucleic acid, different from their free state, which allows them to maintain their protonated form even at physiological pH (10–18). Changes in the protonation state of a nucleobase should significantly influence the ability of a nucleobase to form particular hydrogen bonds and base pairs. The fact of s of interacting nucleobases may modulate their hydrogen bonding interactions (19). The significance of the protonation of nucleobases in biological macromolecules has been discussed in numerous publications (20–24). One of the most recent examples is the spliceosome (25), in which one of the base pairs formed between adenine and cytosine has to be protonated to facilitate the conformational change necessary for the spliceosome's assembly and catalysis.

Nucleobases do not need to be part of a nucleic acid to form a base pair. Free nucleobases and their substituted derivatives are also capable of forming pairs. A plethora of base pairs are observed in crystalline state of these molecules. Here, we asked whether the propensity of nucleobases to form particular base pair types is intrinsic to nucleobases themselves or is forced by the presence of the nucleic acid backbone. We investigated the frequencies of occurrence of particular types of base pairs in small molecule crystals, where constraints imposed by the sugar-phosphate backbone are absent, compared to the frequency of occurrence of analogous base pairs in crystals containing RNA (3,6). Small molecule structural data was obtained from the Cambridge Structural Database (CSD) (26).

The CSD (26) is a repository of structural data obtained from different types of diffraction measurements (neutrons, X-rays etc.) for crystals of small organic molecules and metal-organic compounds. The structures of small molecule crystals are generally more precise and accurate than those of macromolecules due to higher resolution (27).The data for most crystal structures in the CSD are of atomic resolution, the majority being around 0.8 Å. In contrast, the data for RNA, DNA and NA hybrid structures in the PDB have an average resolution of 2.5 Å and only ca. 50 of them have a resolution below 1.0 Å. The CSD data are accurate enough to give reliable information about the geometry of the molecule, and often about the location of the protons, which allows accurate determination of protonation patterns and hydrogen bond geometries of the investigated structures. This gives the opportunity to analyze how preferences to form particular types of base pairs are influenced by different types of protonation, and if the geometry of hydrogen bonding is sensitive to the type of base pair and the protonation state.

The considered nucleobases were adenine, guanine, thymine, cytosine, uracil and, in addition, hypoxanthine (Supplementary Figure S.1 in SI1). The considered nucleobase pairs were only these in which two nucleobases interact with each other through at least two hydrogen bonds lying on the same molecular plane. The analysis was limited to homo-base pairs (like in Figure 1, for example) due to the fact that the majority of crystal structures in the CSD contain only one type of nucleobase per crystal, and structural data for crystals containing hetero-base pairs are very rare. The homo-base pairs comprise more than 20% of all base pairs found in the RNA containing crystal structures in the Protein Data Bank (PDB) (28), as summarized in the RNA Basepair Catalog (3,6). Thus, analysis of homo-base pairs is only still of importance for biological applications. In addition we believe that some general conclusions relevant also to hetero base pairs can be drawn.

## MATERIALS AND METHODS

### Base pair naming scheme

Leontis and Westhof invented (4) the base pair naming scheme in order to describe interactions occurring in RNA. The naming scheme involves the relative orientation of interacting RNA strands (*cis* or *trans*) and names of the nucleobase edges involved in the interactions (Watson–Crick edge, Hoogsteen edge or Sugar edge, see Figure 1). Although the scheme provides an elegant and convenient way of discerning the base pairs present in RNA, it is insufficient for our purposes. First of all, small molecule crystals of nucleobases usually do not contain ribose or phosphate moieties so referring to the strand orientation is simply misleading or sometimes impossible. Moreover quite a few base pairs found in this study have the same *cis*/*trans* orientation and interact with the same edges, but have hydrogen bonds formed between different atoms. This phenomenon was already tackled by Lemieux and Major (29), who further divided the edges into sub-fragments and assigned additional small letters to them. An example is the uracil–uracil *trans* Watson–Crick/Watson–Crick (UU *t*WW) base pair (Figure 1B, right), which in known RNA structures is formed by two N3–H3…O4 hydrogen bonds. By the Lemieux and Major nomenclature, it should be named UU *t*Ww/Ww. However in the small molecule crystals, we observed also UU *t*WW pairs with two N3-H3…O2 hydrogen bonds, which would have the same name of UU tWw/Ww in the Lemieux and Major nomenclature.

Here, we propose a more universal and precise base pair naming scheme, which is more straightforward and can unequivocally describe the interactions found in any base pair. This is done by using the letter abbreviations of nucleobases and interacting atom numbers in the name for a base pair. We modified the naming scheme of Leontis and Westhof to indicate which atoms are directly connected by hydrogen bonds. Explicit atom numbers are used in the base pair name following a predefined numbering scheme (see Supplementary Figure S.2 in SI1). For example, the base pair of adenine presented in Figure 1A according to the proposed nomenclature is:

$$\begin{equation*}{\rm{AA\_fWH\_(16)(67)}}\end{equation*}$$

where the first two letters, AA, indicate the nucleobases that form the given pair (A – adenine, G – guanine, Hx – hypoxanthine, T – thymine, U – uracil, C – cytosine). The next letter, f, indicates the relative orientation of nucleobases: the second base might be either flipped (f) and rotated or moved (m) and rotated (see Supplementary Figure S.3 in SI1). Then, two letters, WH, indicate interacting edges (W – Watson–Crick, H – Hoogsteen, S – Sugar, see Figure 1 and Supplementary Figure S.1 in SI1). To make the naming unambiguous, we used the Watson–Crick > Hoogsteen > Sugar order of edges to decide which molecule would be listed first and which would follow. Finally, two digits in parentheses, (16) and (67), indicate which two atoms form a hydrogen bond (see Supplementary Figure S.2 in SI1 for the numbering of atoms). In this exemplary base pair, the first parenthesis (16) indicates that one hydrogen bond connects nitrogen N1 of the first base with the nitrogen N6 of the second molecule. The second parenthesis (67) points to a hydrogen bond bridging N6 of the first molecule with N7 of the second one. In every parenthesis indicating a hydrogen bond, the first atom number belongs to the first molecule. The parenthesis are arranged by increasing the atom number from the first molecule, then from the second one. For the same bases interacting with the same edges (WW, HH or SS), the molecule from which the lower atom numbers come is listed first, see AA_fWW_(16)(21) (Figure 4), for example. As mentioned above, it is enough to use the first and last part of the name to obtain a unique name, AA_(16)(67) for example. The middle part of the name, here fWH, is defined only to provide a quick orientation for the investigator. The proposed naming scheme can also be used to name hetero-base pairs, see the SI1, p. 5 for the explanation and Supplementary Figure S.4 for the example.

### Search for base pairs in the CSD

We searched only for base pairs formed by two nucleobases interacting with each other through two or more hydrogen bonds and forming more or less planar structures.

We took into account either free (unsubstituted) nucleobases or nucleobases that are substituted with the substituent having a carbon atom at the site of a glycosidic bond (C1′–N9 for purines and C1′–N1 for pyrimidines) and have no substituents at other sites. In addition,we allowed each atom of a nucleobase to have an unspecified number of hydrogen atoms attached, including no hydrogen atoms. This allowed us to include forms with various protonation patterns.

To search for structures, we used the CCDC ConQuest ver. 1.23, database version 5.39 (August 2018). The search was divided into four steps in which each query narrowed down the search space for the next step:

1. **Search for all crystal structures containing molecules that had nucleobases incorporated into their structure**. No hydrogen atom positions were specified, bond types were set to ‘Any.’ An atom at the glycosidic position (bonded to the N9 nitrogen for purines and N1 for pyrimidines) was defined as ‘R,’ meaning ‘C or H’, or no atom was given. It is important to stress that the ‘R’ used here in reference to making queries to CSD means something different than later on in the text, when R indicates substituents other than hydrogen connected to the nucleobase through carbon. Organometallic compounds were excluded from the search, as well as structures without determined 3D coordinates.
2. **Exclusion of molecules that were substituted with atoms other than hydrogen in places other than the glycosidic bonds**. This was done by making a set of queries in which the structure of the nucleobase was drawn as in the first step of the search, but one atom other than N9 for purines and N1 for pyrimidines was substituted with ‘Z’, meaning ‘other than H’. Those queries were then placed together in the ‘must not have’ box in the ‘Combine queries’ section.
3. **Defining base pairs**. After choosing the set of suitable crystal structures for each type of nucleobase, we searched for specific types of base pairs, defining contacts between two hydrogen donor and acceptor atoms (D…A) that are within the sum of van der Waals radii +1 Å, and have an angle between planes formed by nucleobase rings and hydrogen bonds (D…A) smaller than 45°.
4. **Hydrogen bonds and C1′…C1′ distances**. As for the statistics of hydrogen bonds (D–H, H…A, D…A lengths and D–H…A angle), a broad query was made that did not specify, which atom of the molecule acts as a donor or acceptor. Thus, any type of protonation was allowed in each search.

Sometimes the same crystal structure was deposited in the CSD more than once. All such depositions are given refcodes with identical letters but different numbers e.g. ABCDEF01, ABCDEF02 and constitute what we call a ‘refcode family’. For a refcode family consisting of more than one entry, the entire family was counted as a single occurrence for calculations of frequencies of occurrence. For calculations of average hydrogen bond lengths and angles, we took data from every deposition. As the X-ray measurement comprising the majority of all the datasets deposited in the CSD (among all the analyzed structures only six unique ones were from neutron diffraction) are known to give covalent bond lengths to hydrogen atoms which are too short by ∼0.2 Å (30), all the D–H bonds were extended to their averaged lengths obtained from neutron measurements (31).

For the pairs having symmetrical topology the average hydrogen bond lengths and angles were calculated by taking into account all unique hydrogen bonds. The structures without hydrogen positions determined were counted into frequencies of occurrence, and into D…A and C1′…C1′ distance statistics, but obviously could not be included in D–H and H…A lengths and D–H…A angle statistics. The number of such structures differs by the nucleobase type but usually does not exceed 10%. Crystal structures containing more than one type of base pair were counted in every particular pair frequency of occurrence statistics. For this reason, the total number of analyzed base pairs is larger than the total number of analyzed unique structures containing base pairs.

Not all of the investigated structures have all the hydrogen atoms modeled. If there were no hydrogen positions determined (or only some hydrogen atoms were missed), we classified the structure to the particular base pair(s) and listed it as ‘R?’ (substituted, no hydrogen positions determined) or ‘No R?’ (free, no hydrogen positions determined), even if our chemical intuition clearly stated where the hydrogens should be, or the fact of formation of particular base pair required certain type of protonation.

No cut-off for allowed values of the *R*-factor was imposed during the searches. Nevertheless, the vast majority of the investigated structures had *R*-factors below 10%, which is usually accepted for small molecule crystals as being an indicator of good quality. A few structures (∼5%) had higher *R*-factors (up to 20%) and these were included in the analysis because it appeared that base pair interaction lengths found in them were no different than the mean lengths for the entire population of particular base pair type.

The database search algorithm allowed us to find base pairs in which one nucleobase was substituted and the other was free. No such base pair was found in the CSD. Therefore all pairs analyzed here are truly homo-base pairs, also from the point of view of the presence or absence of a substituent.

The frequencies of RNA-specific base pairs, as well as the exemplary C1′–C1′ distances, were taken from the RNA Basepair Catalog (3,6). Base pairs with only one hydrogen bond between nucleobases and other hydrogen bonds formed between nucleobase and sugar were excluded from the analysis.

## RESULTS

### General remarks

There are more than 1400 unique crystal structures deposited in the CSD which contain canonical nucleobases (adenine, guanine, thymine, uracil, cytosine) or their N1/N9-derivatives. Structures containing each distinct type of nucleobase occur with more or less comparable frequencies. Structures with adenine or its derivatives are the most common (399) and those containing guanine (and derivatives) are the least frequent (140) (Table 1). For the sake of comparison, we have added hypoxanthine to our analysis as it is one of the non-canonical bases that is observed in native nucleic acids. Structures containing hypoxanthine or its N9-derivatives are definitely less frequent in the CSD, in which there are only 36.

**Table 1.** Number of unique crystal structures containing nucleobases or their N1/N9-derivatives with division to those containing or not containing base pairs and differentiation between the state of the base pair (neutral or charged, substituted or not). Neutral means true neutral or unknown due to lack of determined hydrogen atom positions, charged–charged base pair due to the presence of one or more additional protons (positively charged), or lack of thereof (negatively charged)

	With base pairs						Without base pairs					
	A	G	Hx	T	U	C	A	G	Hx	T	U	C
Neutral substituted	176	67	10	117	108	37	90	42	21	187	99	94
Charged substituted	30	9	0	0	0	19	30	3	2	0	0	63
Neutral non-substituted	14	4	1	11	2	8	3	0	0	4	1	5
Charged non-substituted	36	10	0	1	0	44	20	5	2	0	0	44
**Total**	256	90	11	129	110	108	143	50	25	191	100	206

In general, structures containing N1/N9-derivatives of nucleobases are more numerous than those containing free (not substituted) bases. The majority of substituted bases (∼85%) appear in the neutral form, whereas about two thirds of free bases are charged. This was most probably governed by human interest, but also could be due to difficulties in (co-)crystallization of free neutral bases due to their low solubility in commonly used solvents.

Nucleobases are believed to have a high propensity to form base pairs. In the case of small molecule crystals, the formation of a base pair must compete with many other possible associations of bases with themselves and the remaining parts of a molecule, or with other molecular components of the crystal. Despite all these competitive processes, homo-base pairs are frequently observed. The largest percentage of structures containing pairs is observed for adenine and guanine (64%) and for uracil (52%) while thymine moieties form homo-base pairs less frequently (40%). For cytosine and hypoxanthine and their derivatives, homo-base pairs are observed in only ∼30% of their crystal structures (Table 1).

An interesting subgroup of structures containing derivatives of nucleobases are nucleosides and nucleotides. There were 626 such structures in the CSD, and 248 of them contained homo-base pairs. In addition, some structures had base pairs involving sugar moiety, however the geometry of such interactions often differed considerably from what occurs in RNA. The wide range of possibilities coupled with a small number of structures to consider makes further investigation into nucleosides and nucleotides unlikely to give any sensible results.

### Homo-base pairs observed in the small molecule crystals

**Figure 2.**
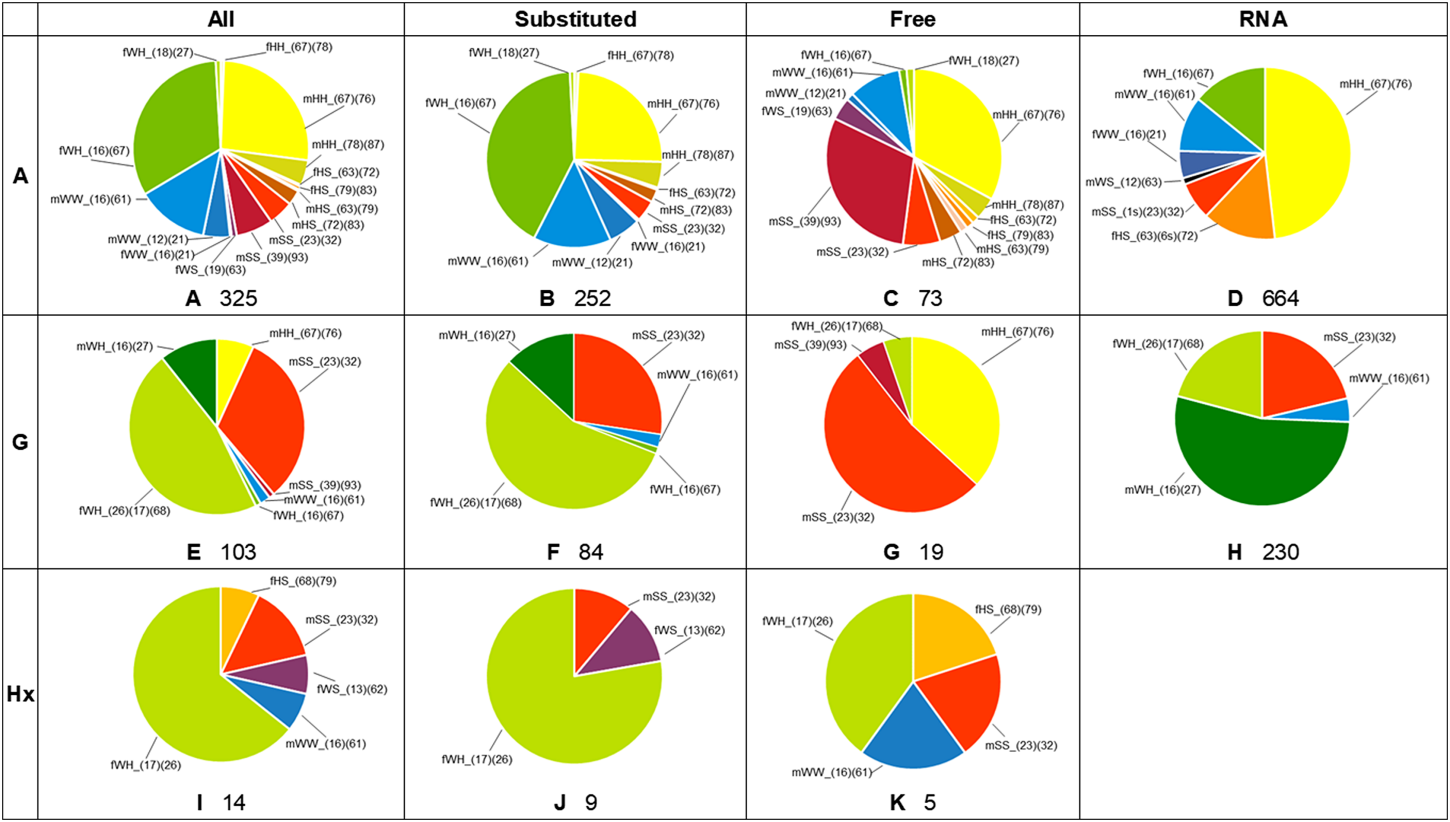
Frequencies of occurrences of homo-base pairs for purines. The pie-charts present the share of frequencies of occurrences of particular types of pairs among all base pairs of particular nucleobase, among pairs with substituted nucleobases, and among pairs with free bases. The fourth column denoted as ‘RNA’ represents the number of nucleobases pairs observed in RNA as indicated in RNA Basepair Catalog (3,6).The base pairs are color-coded for easy visual comparison. A base pair formed by two Watson–Crick edges is colored in blue, two Hoogsteen edges yellow and two Sugar edges – red. The mixed edge base pair shares bear the color that would be an effect of mixing colors corresponding to the edges participating in the formation of the base pair, i.e., base pair with Watson–Crick (blue) edge and Hoogsteen (yellow) edge will be marked green. For more than one base pair formed by particular edges, different shades of the corresponding color were used. If possible, base pairs occurring among different nucleobases but having analogous geometry, i.e. mHH_(67)(76) in both adenine and guanine, were given the same color. The black-colored parts in the charts of RNA section indicate the occurrence of pairs that do not appear in small molecule crystals, but potentially fit our categories. The numbers below the charts indicate the total number of pairs from unique structures belonging to the particular group.

**Figure 3.**
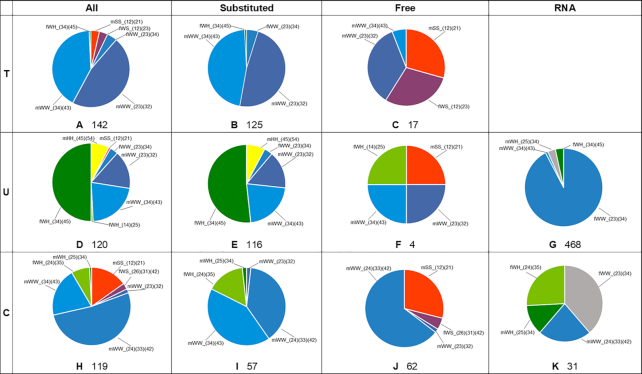
Frequencies of occurrences of homo-base pairs for pyrimidines. The pie-charts present the share of frequencies of occurrences of particular types of pairs among all base pairs of particular nucleobase, among pairs with substituted nucleobases, and among pairs with free bases. The fourth column denoted as ‘RNA’ represents the number of nucleobases pairs observed in RNA as indicated in RNA Basepair Catalog (3,6).The base pairs are color-coded for easy visual comparison. A base pair formed by two Watson–Crick edges is colored in blue, two Hoogsteen edges yellow and two Sugar edges – red. The mixed edge base pair shares bear the color that would be an effect of mixing colors corresponding to the edges participating in the formation of the base pair, i.e., base pair with Watson–Crick (blue) edge and Hoogsteen (yellow) edge will be marked green. For more than one base pair formed by particular edges, different shades of the corresponding color were used. If possible, base pairs occurring among different nucleobases but having analogous geometry, i.e. mHH_(67)(76) in both adenine and guanine, were given the same color. The black-colored parts in the charts of RNA section indicate the occurrence of pairs that do not appear in small molecule crystals, but potentially fit our categories. The numbers below the charts indicate the total number of pairs from unique structures belonging to the particular group.

**Figure 4.**
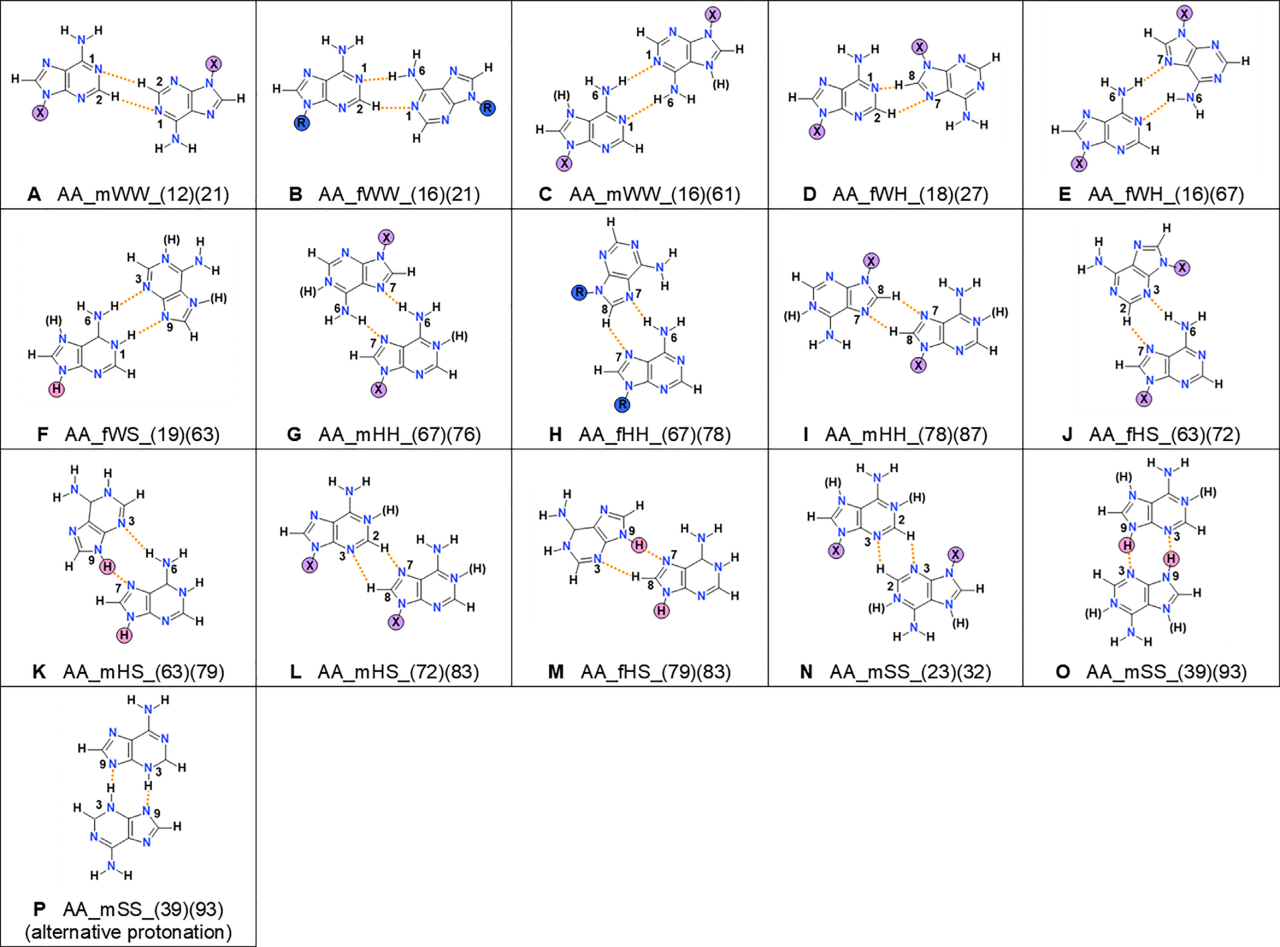
Types of adenine–adenine base pairs found in small molecule crystals containing adenine or its N9-derivatives. As for the circled letters: X denotes that the pair type was observed for both, substituted or free nucleobases; H denotes that only free bases form this type of base pair, even if there is no steric clash preventing substituted ones to form; R denotes that this particular pair is formed exclusively by substituted nucleobases. The (H) denotes where the additional protons may appear. The examples of existing structures from CSD can be found in Supplementary Figure S.8.

**Figure 5.**
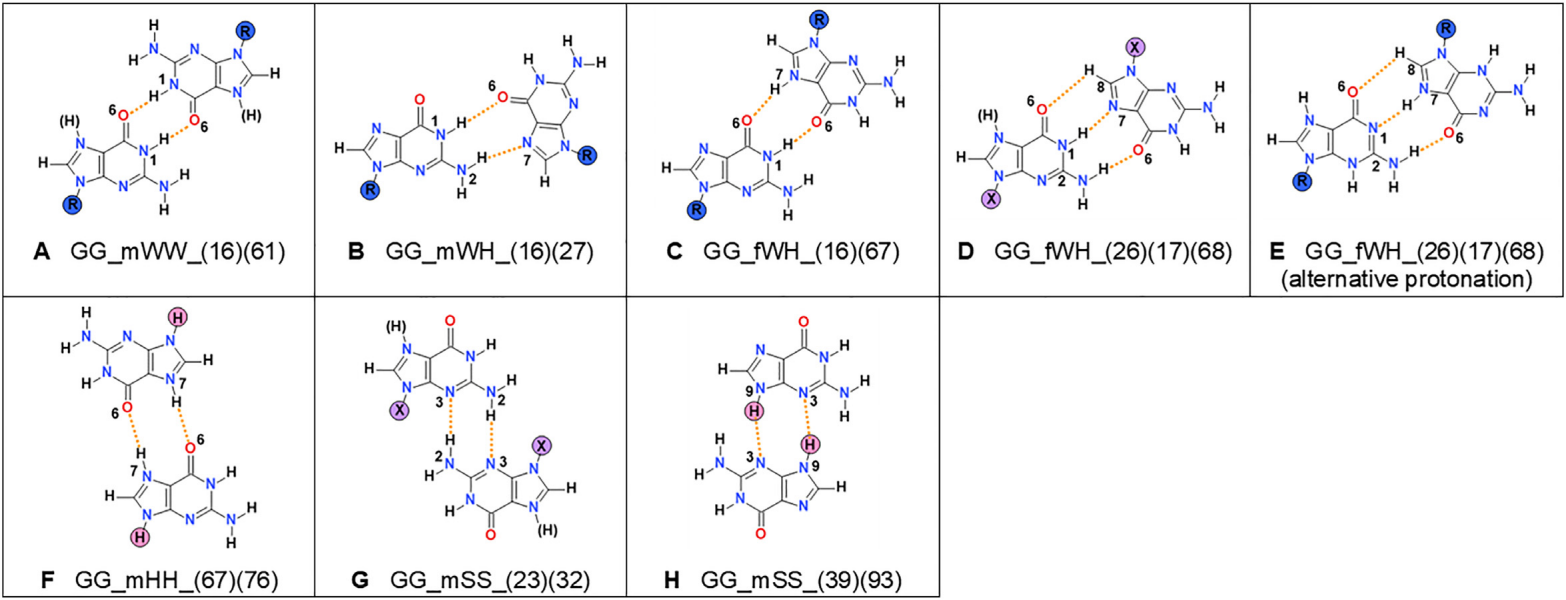
Types of guanine–guanine base pairs found in small molecule crystals containing guanine or its N9-derivatives. For details, see Figure 4 caption. The examples of existing structures from CSD can be found in Supplementary Figure S.9.

**Figure 6.**
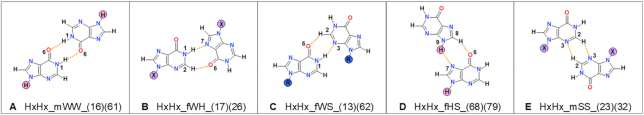
Types of hypoxanthine-hypoxanthine base pairs found in small molecule crystals containing hypoxanthine or its N9-derivatives. For details, see Figure 4 caption. The examples of existing structures from CSD can be found in Supplementary Figure S.10.

**Figure 7.**
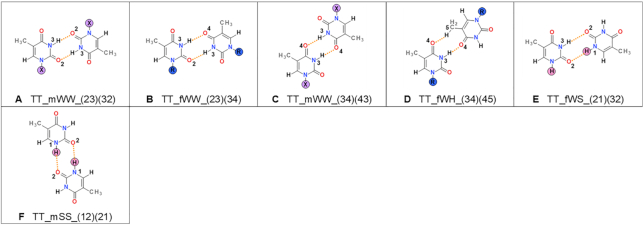
Types of thymine–thymine base pairs found in small molecule crystals containing thymine or its N1-derivatives. For details, see Figure 4 caption. The examples of existing structures from CSD can be found in Supplementary Figure S.11.

**Figure 8.**
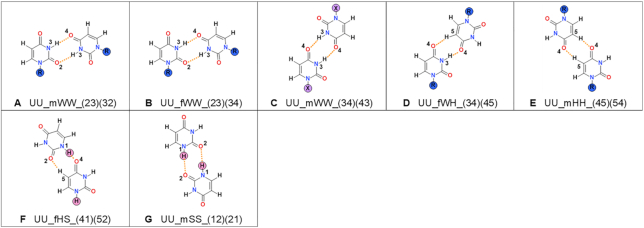
Types of uracil–uracil base pairs found in small molecule crystals containing uracil or its N1-derivatives. For details, see Figure 4 caption. The examples of existing structures from CSD can be found in Supplementary Figure S.12.

**Figure 9.**
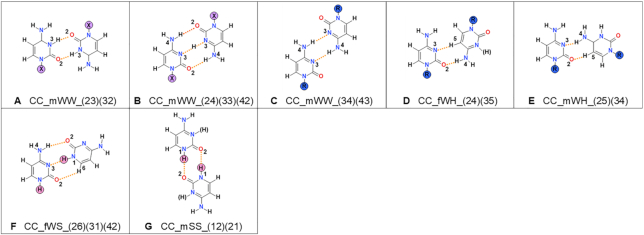
Types of cytosine–cytosine base pairs found in small molecule crystals containing cytosine or its N1-derivatives. For details, see Figure 4 caption. The examples of existing structures from CSD can be found in Supplementary Figure S.13.

The results of our survey of CSD are divided into distinct nucleobases and begins with the analysis of frequencies of occurrence (Figures 2 and 3) of identified base pair types (Figures 4–9). Then a comment on the hydrogen bond types and geometry (Supplementary Tables S.1–6) is given. Finally, observed protonation patterns and charges associated with them are described (Tables 2–7). A compilation of all the findings is presented in SI2, which is designed to be an easy to use atlas of base pair types found in small molecule crystals.

**Table 2.** Protonation patterns for adenine. The number of particular base pair types having a particular type of protonation observed in a unique set of small molecule crystal structures is given. The protonation patterns are defined in the column headers, where the numbers 1 3 7 9 correspond to the nitrogen atoms present in purine rings, and 1 3 – nitrogen atoms in pyrimidine rings. If the number is free, then this particular nitrogen is not protonated, if the letter ‘H’ is present, then this position is protonated. If there is ‘R’, then it indicates the derivative of a particular nucleobase. Question marks represent structures without determined proton positions. One line in the header means both bases are protonated the same way, two lines define protonation patterns which differ for each base from the pair. If there is ‘NA’ – pairs cannot be formed due to the presence of an R substituent. If there is ‘-‘ in the table, it denotes that for some reason this base pair cannot form with this particular type of protonation (either due to steric clash between two protons or lack of a proton to form hydrogen bond). If the number is 0 then no such structures were observed, but there is no steric clash preventing a base pair from forming neither is there a missing proton which would be necessary to form a hydrogen bond

Adenine	1 3 7 9R	1H 3 7 9R	R?	1 3 7 9H	1H 3 7 9H	1 3H 7H 9	1H 3 7H 9H	1H 3 7H 9	1 3 7H 9	1 3 7 9H 1H 3 7 9H	1 3 7H 9 1H 3 7 9H	No R?	Sum
AA_mWW_(12)(21)	13	-	2	1	-	0	-	-	0	-	-	0	16
AA_fWW_(16)(21)	1	-	0	0	-	0	-	-	0	-	-	0	1
AA_mWW_(16)(61)	32	-	4	4	-	0	-	-	3	-	-	0	43
AA_fWH_(18)(27)	2	-	0	1	-	-	-	-	-	0	0	0	3
AA_fWH_(16)(67)	98	-	7	1	-	-	-	-	-	0	0	0	106
AA_fWS_(19)(63)	NA	NA	NA	-	-	-	-	2	-	-	1	0	3
AA_mHH_(67)(76)	27	23	12	2	19	-	-	-	-	1	-	2	86
AA_fHH_(67)(78)	2	0	0	0	0	-	-	-	-	0	-	0	2
AA_mHH_(78)(87)	11	0	1	2	1	-	-	-	-	0	-	0	15
AA_fHS_(63)(72)	1	0	0	1	0	-	-	-	-	0	0	0	2
AA_mHS_(63)(79)	NA	NA	NA	0	1	-	-	-	-	0	0	0	1
AA_mHS_(72)(83)	5	1	0	0	3	-	-	-	-	0	0	0	9
AA_fHS_(79)(83)	NA	NA	NA	0	1	-	-	-	-	0	0	0	1
AA_mSS_(23)(32)	5	2	3	0	3	-	1	0	0	0	0	1	15
AA_mSS_(39)(93)	NA	NA	NA	6	10	1	3	-	-	1	-	1	22
**Sum**	197	26	29	18	38	1	4	2	3	2	1	4	325

**Table 3.** Protonation patterns for guanine. For details, see Table 2 caption

Guanine	1H 3 7 9R	1H 3 7H 9R	1 3H 7H 9R	1H 3 7 9R 1H 3 7H 9R	R?	1H 3 7 9H	1H 3 7H 9H	1H 3 7H 9	1H 3 7 9H 1H 3 7H 9H	No R?	Sum
GG_mWW_(16)(61)	1	1	-	0	0	0	0	0	0	0	2
GG_mWH_(16)(27)	11	-	-	0	0	0	-	-	0	0	11
GG_fWH_(16)(67)	-	0	-	1	0	-	0	0	0	0	1
GG_fWH_(26)(17)(68)	43	-	1	1	2	1	-	-	0	1	49
GG_mHH_(67)(76)	-	0	0	-	0	-	5	1	-	1	7
GG_mSS_(23)(32)	15	4	-	0	3	0	10	0	1	0	33
GG_mSS_(39)(93)	NA	NA	NA	NA	NA	1	0	-	0	0	1
**Sum**	70	5	1	2	5	2	15	1	1	1	103

**Table 4.** Protonation patterns for hypoxanthine. For details, see Table 2 caption

Hypoxanthine	1H 3 7 9R	R?	1H 3 7 9H	No R?	Sum
HxHx_mWW_(16)(61)	0	0	1	0	1
HxHx_fWH_(17)(26)	7	0	0	2	9
HxHx_fWS_(13)(62)	1	0	0	0	1
HxHx_fHS_(68)(79)	NA	NA	1	0	1
HxHx_mSS_(23)(32)	1	0	1	0	2
**Sum**	9	0	3	2	14

**Table 5.** Protonation patterns for thymine. For details, see Table 2 caption

Thymine	1R 3H	R?	1H 3H	1 3H	No R?	Sum
TT_mWW_(23)(32)	52	8	6	0	0	66
TT_fWW_(23)(34)	6	0	0	0	0	6
TT_mWW_(34)(43)	57	1	0	1	0	59
TT_fWH_(34)(45)	1	0	0	0	0	1
TT_fWS_(21)(32)	NA	NA	4	0	1	5
TT_mSS_(12)(21)	NA	NA	5	0	0	5
**Sum**	116	9	15	1	1	142

**Table 6.** Protonation patterns for uracil. For details, see Table 2 caption

Uracil	1R 3H	R?	1H 3H	No R?	Sum
UU_mWW_(23)(32)	17	1	1	0	19
UU_fWW_(23)(34)	3	1	0	0	4
UU_mWW_(34)(43)	25	0	1	0	26
UU_fWH_(34)(45)	53	7	0	0	60
UU_mHH_(45)(54)	9	0	0	0	9
UU_mHS_(41)(52)	NA	NA	1	0	1
UU_mSS_(12)(21)	NA	NA	1	0	1
**Sum**	107	9	4	0	120

**Table 7.** Protonation patterns for cytosine. For details, see Table 2 caption

Cytosine	1R 3	1R 3H	1R 3 1R 3H	R?	1H 3	1H 3H	1H 3 1H 3H	No R?	Sum
CC_mWW_(23)(32)	-	1	-	0	-	1	-	0	2
CC_mWW_(24)(33)(42)	3	-	15	4	3	-	36	1	62
CC_mWW_(34)(43)	24	-	-	0	0	-	-	0	24
CC_fWH_(24)(35)	8	0	1	0	0	-	0	0	9
CC_mWH_(25)(34)	0	-	1	0	0	-	0	0	1
CC_fWS_(26)(31)(42)	NA	NA	NA	NA	3	-	0	0	3
CC_mSS_(12)(21)	NA	NA	NA	NA	5	10	2	1	18
**Sum**	35	1	17	4	11	11	38	2	119

#### Adenine

There are 399 different crystal structures containing a free adenine molecule or its N9-derivatives. Among these structures, 143 contain no adenine-adenine base pairs, while in 256, at least one type of base pair is present (Table 1). Altogether we found 325 adenine homo-base pairs (Figure 2) grouped into 15 different types (Figure 4).

The most commonly occurring pairs for adenine and its N9-derivatives are AA_fWH_(16)(67), AA_mHH_(67)(76) and AA_mWW_(16)(61), see Figure 2A. The ratio stays very similar if we consider only N9-derivatives of adenine (Figure 2B). For free adenine, the AA_mHH_(67)(76) and AA_mWW_(16)(61) base pairs retain more or less similar frequency as N9-derivatives (Figure 2C). The new pair that becomes frequent in structures with free adenine is AA_mSS_(39)(93), which cannot be formed when N9 is substituted. Three other types of adenine pairs which cannot be formed by two N9-substituted adenines also appear, but they were rarely observed.

Among adenine pairs, there are six types formed exclusively through N–H…N hydrogen bonds (Figure 4 and Supplementary Table S.1). Molecules in five other types interact only through C–H…N hydrogen bonds, and there are four base pairs displaying both types of hydrogen bonds. In the observed adenine pairs, hydrogen bonds of the C–H…N kind are quite long and do not have optimal orientation compared to the electron lone pair of the nitrogen acceptor. The C…N distances averaged per each pair type are in the range of 3.3–3.9 Å, and the C–H…N angles are in the range of 118–145°. The N–H…N bonds in analyzed AA pairs have average N…N distances in the range of 2.8–3.2 Å and the average N–H…N angles in the range of 142–175°. They are slightly longer than the shortest known N–H…N hydrogen bonds (32) which have the N…N distance of 2.6 Å (33).

All the above statistics of hydrogen bond geometries were calculated without subdivision into free or substituted nucleobases, or into neutral or protonated nucleobases. An attempt to do so led to statistically identical results, mostly because of the small data set. Only for the AA_mHH_(67)(76) pairs can some statistically significant conclusions be drawn, and in this case there is no influence of substitution of N9 nor protonation of the molecule on the lengths of hydrogen bonds.

The base pairs are formed either by molecules in their neutral or protonated (cationic) form—around 10% of all the adenine base pairs contain at least one charged base. As adenine has more than one possible place to accept the additional proton, various protonation patterns are observed (Table 2 and Supplementary Figure S.2 in SI1). The protonation of the N1 position dominates and this is the only protonation pattern observed among substituted adenines. As for the base pairs with free molecules, other protonation patterns are also observed, even doubly protonated ones. Usually, free base pairs are protonated at the N1 and N7 positions. The majority of protonated adenine base pairs consists of molecules having identical protonation patterns, with only two structures (the CSD refcodes of PANSAA and EVIFIY) having pairs built from differently protonated molecules. The AA_mHH_(67)(76) base pair is the one that has the strongest preference for incorporating the N1-protonated bases, especially when it consists of free adenines.

In our survey, we found that all of the observed pair types can be formed by two neutral adenine molecules, and only in one case, AA_fWS_(19)(63), this would require the transfer of a proton from the N9 to the N1 position. Most of the observed pair types can still be formed if one of the molecules or both molecules are protonated in some way (Figure 4, Table 2). We did not find an adenine base pair type that specifically required at least one of the molecules to be protonated. Despite there being base pair types that can be formed by either neutral or protonated forms, they are strongly preferred by only one form (see, e.g. AA_mHH_(67)(76) for free molecules in Table 2). There is a visible difference between base pairs that are preferred by neutral molecules and those preferred by charged ones (Supplementary Figure S.5 in SI1).

#### Guanine

There are 140 unique structures with guanine or its N9-derivatives present in the CSD, among which 90 contain at least one type of guanine-guanine base pair (Table 1). Among structures containing guanine homo-base pairs, we found 103 such pairs (Figure 2), which could be divided into seven types (Figure 5).

The two most common types of pairs present among guanine and its N9-derivative structures deposited in CSD are GG_fWH_(26)(17)(68) and GG_mSS_(23)(32) (Figure 2). In the case of N9-subsitituted guanines, the GG_fWH_(26)(17)(68) pairs are the most frequent (56%). The GG_cSS_(23)(32) (27%) and the GG_mWH_(16)(27) (13%) pairs are also quite common. Free guanines prefer to form the GG_mSS_(23)(32) pairs (53%) and then GG_mHH_(67)(76) pairs (37%). There is only one base pair type for free guanines which cannot be formed by N9-derivatives, GG_mSS_(39)(93), but was observed only in one crystal structure.

The majority of the pairs have hydrogen bonds only with nitrogen as a proton donor, and nitrogen or oxygen as a proton acceptor (Figure 5). There are three types of base pairs with only N–H…O types of bonds, two with mixed types, the remaining two containing only N–H…N bonds. The average N–H…N bonds tend to be a little shorter for guanine than for adenine (Supplementary Table S.2). Their average N…N distances are in the range of 2.8–3.04 Å and the average N–H…N angles in the range of 161–172°. The N–H..O bonds, like the N–H..N bonds, are of moderate length (32). Most of them have the averaged N…O distances in the range of 2.79–2.90 Å and N–H…O angles (152–170°) (32). The C–H…O bonds are only observed in the GG_fWH_(26)(17)(68) pairs which contain in addition two other hydrogen bonds: N–H…O and N–H…N. The C–H…O bond is quite an exception from the whole statistics as it is rather lengthy (3.4(1) Å), and its angle barely fits into the common hydrogen bond definition (118(6)°). There is one exceptional pair observed in only one structure (the CSD refcode ASUVIU) in which the N–H…O bonds are a bit shorter - the GG_fWH_(16)(67) pair build from a mixture of neutral and the N7-protonated derivative of guanine.

Similarly as for adenine, only for one type of pair, here the GG_mSS_(23)(32) pair, can meaningful statistical analysis of subgroups (free and substituted, protonated or neutral) be carried out. The analysis confirmed that there is no statistically significant difference between the particular subgroups. Again, hydrogen bonds formed between two protonated bases seem to have the same geometry as for neutral bases.

Almost all the cations observed in guanine pairs are singly protonated at the N7 position, the only exception being the structure with refcode REPGAV which is protonated at the N3 and N7 positions, but lacks the standard N1 proton. The most popular type of guanine pair among the protonated forms is GG_mSS_(23)(32). In fact GG_mSS_(23)(32) is the only guanine pair which is quite common among both substituted and free guanines, and among neutral and protonated ones.

For guanine, not all base pairs can be formed by neutral molecules. In our survey we found two base pairs that require the additional proton to be present at N7 to form a hydrogen bond: GG_fWH_(16)(67) and GG_mHH_(67)(76). The first is observed only in one crystal structure and is formed by substituted nucleobases with different protonation patterns, and the second can only be found among free nucleobases (Table 3). The preference for forming particular base pairs differs between neutral and charged molecules (Supplementary Figure S.6 in SI1), and favors Sugar edge for charged nucleobases.

#### Hypoxanthine

The number of structures containing hypoxanthine or its N9-derivatives is relatively small compared to other nucleobases included in our study—there are only 36 such structures (Table 1), and 11 of them contain at least one of the five types (Figure 2) of hypoxanthine pairs identified in small molecule crystals (Figure 6).

Due to the small number of occurrences of hypoxanthine and its derivatives in the CSD, the only thing that can be said with any certainty is that the HxHx_fWH_(17)(26) base pair is preferred (Figure 2). Thus, hypoxanthine behaves more like guanine than adenine while forming pairs even though there is no amino group at the C2 position and it is the C–H group that donates a proton to the C2–H…O6 bond.

Despite the small number of structures present in the CSD, hypoxanthine presents a diverse range of different types of base pair geometries (Figure 6, Supplementary Table S.3). Three of the pairs have ‘mixed’ hydrogen bonds comprising one C–H…O and either an N–H…N, or an N–H…O or a C–H…N hydrogen bond. The most frequent N1–H1…N7 bond type has geometry very similar to the one for the analogous bond in the GG_fWH_(26)(17)(68) pairs (Figures 6 and 5). Due to hypoxanthine lacking the NH_2_ group, the pairs it forms have two hydrogen bonds instead of three because the bond angle C8–H8…O6 is 10–20° below the commonly accepted 120° threshold for hydrogen bonds.

All hypoxanthine base pairs are formed by nucleobases in their neutral state (Table 4).

#### Thymine

We found 320 unique structures of thymine or its N1-derivatives in the CSD, and 129 of them contain thymine base pairs (Table 1). Among these, we identified 142 crystallographically unique thymine-thymine pairs (Figure 3), which could be classified into six types (Figure 7).

For thymine, pairs incorporating Watson–Crick edge in their interactions are the most common, namely TT_mWW_(23)(32) and TT_mWW_(34)(43) (Figure 3). There are far more substituted thymine base pairs (125) than free (unsubstituted) base pairs (17). Among free base pairs there are two new types, TT_mSS_(12)(21) and TT_fWS_(12)(23), and these pairs could not have been formed by substituted thymine. The pairs are almost as frequent as the TT_mWW_(23)(32), whereas TT_mWW_(34)(43) are less frequent in free thymine structures.

Thymine forms almost exclusively N–H…O types of hydrogen bonds, Figure 7. Their averaged N…O distances are in the range of 2.82–3.00 Å and the D-H…A angles of 167–173° (Supplementary Table S.4). Only one base pair, and one structure that forms it, contains a C-H hydrogen bond donor.

Almost all observed thymine pairs are formed by neutral molecules (Table 5) with one exception, which is a pair containing thymine deprotonated at N1 (Refcode KTHYMT). This structure contains, besides the thymine anion, three water molecules and potassium cation, and the thymine anion forms a TT_mWW_(34)(43) base pair. Despite being formed between two anions, the base pair in this structure has hydrogen bond lengths which do not differ from the mean for this pair type. The deprotonated N1 does not partake in the formation of base pair, the K^+^ cations do not mediate in this interaction, and the base pair in this structure forms no differently than in the other structures.

#### Uracil

The CSD contains 210 unique structures with uracil or its N1-derivatives (Table 1). Of these, 110 structures contain 120 crystallographically unique base pairs (Figure 3) classified into seven types (Figure 8).

The most common type of pair for N1-substituted uracil derivatives is UU_fWH_(34)(45), named in literature as the Calcutta pair, (Figure 8). The analogous type cannot be formed by thymine because of the presence of the methyl group at C5 position, which prevents hydrogen-bonding at this position. The UU_mWW_(34)(43) and UU_mWW_(23)(32) pairs are also quite common in uracil homo-base pairs, but they are about half as frequent as the analogous types in the case of substituted thymine pairs.

Similarly to thymine, the number of unique structures containing pairs of substituted uracil derivatives is far larger, namely 108, compared to free uracil – which are only two (Table 1). Among pairs involving free uracil, the Calcutta pair type is not present, but the UU_mWW_(34)(43) and UU_mWW_(23)(32) pairs are found. The first pair co-exists with the UU_mSS_(12)(21) pair in crystal structures with the refcode INONAC, the second pair coexists with the UU_fHS_(41)(52) pair in the URACIL structure. Both of these can only be formed by free uracil molecules.

There are only two types of hydrogen bonds present among uracil base pairs, N–H…O and C–H…N (Figure 8), but the frequencies of occurrence of the two types of hydrogen bonds are comparable. The average N…O distances are in the range of 2.81–2.92 Å and the D–H…A angles of 143–170° (Supplementary Table S.5). The pair with the shortest averaged N…O distance has the smallest (least optimal) average D–H…A angle and,nterestingly, is the most frequent Calcutta pair.

All observed pairs involving uracil are formed by neutral bases (Table 6).

#### Cytosine

There are 314 crystal structures of cytosine or its N1-derivatives in the CSD and only 108 (Table 1) contain at least one of the seven observed types of base pairs (Figures 3 and 9).

The three most common types of pairs formed by cytosine are CC_mWW_(24)(33)(42), CC_mWW_(34)(43) and CC_mSS_(12)(21) (Figure 3), with the first one being the most common. Cytosine is the only pyrimidine with comparable numbers of structures containing base pairs of N1-substituted and of free nucleobases (56:52 ratio). Substituted cytosine molecules usually form either the CC_mWW_(24)(33)(42) or the CC_mWW_(34)(43) type of pairs, with similar frequency (38.6% and 42.1%, respectively). The CC_fWH_(24)(35) base pair is the next most common among substituted molecules and amounts to 15.8%. In structures having free cytosine molecules, the CC_mWW_(24)(33)(42) type is even more frequent (64.5%), and the remaining types are almost completely replaced by the pairs involving the Sugar edge. The latter can be built exclusively by free molecules and among them the CC_mSS_(12)(21) is the most common (29.0%).

Cytosine pairs are connected either by N–H…O, N–H…N, C–H…O or C–H…N bond types (Figure 9). The N–H…O bonds are characterized by average D…A distances in the range of 2.77–3.00 and average D–H…A angles of about 151 to 173° (Supplementary Table S.6). The N–H…N have ranges of 2.82–3.02 Å and 169° to 174°. The C–H…O and C–H…N bonds are more elongated (longer than 3.5 Å) and also differ even more from the 180° optimum for hydrogen bond.

Cytosine is the only nucleobase which frequently forms pairs between nucleobases in different protonation states, almost as frequently as pairs with identically protonated molecules (Table 7). This is because of the high frequency of the hemi-protonated base pair CC_mWW_(24)(33)(42), which has the additional proton located between the N3 atoms and is shared by both cytosines.

Three cytosine base pairs are related to each other - CC_mWW_(23)(32), CC_mWW_(24)(33)(42) and CC_mWW_(34)(43) (Figure 9A–C). Their geometries are very similar, differing only by a small shift of the nucleobases relative to each other and a change in their protonation. The CC_mWW_(23)(32) base pair can only be formed if both nucleobases are protonated at N3, CC_mWW_(24)(33)(42) requires only one nucleobase to be protonated and CC_mWW_(34)(43) can only be present if both molecules are in their neutral state. The neutral base pair has the shortest hydrogen bonds among the three, followed by the semi-protonated base pair, while the longest hydrogen bonds belong to the CC_mWW_(23)(32) made by two cations. The length of the hydrogen bonds does not fully reflect the frequency of occurrence of particular base pairs—the CC_mWW_(24)(33)(42) is undoubtedly the most frequent, followed by the CC_mWW_(34)(43).

The CC_mWH_(25)(34) base pair occurs only once and forms from substituted bases with mixed protonation. In this case the protonation is not required for hydrogen-bonding, but the structure representing this particular base pair happens to be protonated.

When N1 is not blocked by a substituent, two protonated cytosine molecules prefer to form the CC_mSS_(12)(21) pair instead of CC_mWW_(23)(32) (Supplementary Figure S.7). The CC_mSS_(12)(21) pair is also the most frequent among neutral free cytosines.

### Comparison to frequencies of occurrence of homo-base pairs in RNA

The frequency of occurrence of comparable base pairs in crystal structures containing RNA (3,6) and in crystal structures of small organic molecules deposited in the CSD vary by nucleobase. Here we will only compare the occurrence frequencies of base pairs from the CSD containing substituted nucleobases (last column of Figures 2 and 3), as these are most comparable to base pairs observed in RNA.

For adenine the AA_fWH_(16)(67) and AA_mHH_(67)(76) base pairs are the most frequent both among small molecules (41.46% and 24.60%) and in RNA (14.16% and 48.19%), though with reversed order of frequency. The third base pair that appears frequently in both systems, AA_mWW_(16)(61), is a little more common for small molecules (13.23%) than for RNA (10.39%). The fourth base pair found in both systems is named AA_mSS_(1s)(23)(32) in RNA (7.08%) and AA_mSS_(23)(32) in small molecules crystal structures (3.97%)—this difference stems from the fact that in RNA crystal structures the base pair is held together by one more hydrogen bond linking the first nucleobase with a sugar molecule of the other nucleobase. The last base pair appearing in both systems is AA_fWW_(16)(21)—we found only one such base pair among small molecule crystals, but in RNA crystal structures it is a little more frequent (5.12%). The rest of the base pairs occurring in crystals of small molecules do not occur in crystal structures of RNA. The only base pair that is RNA-exclusive is AA_mWS_(12)(63) and it makes up only 1.20% of all RNA crystal structures.

For guanine there are four common base pairs for both small molecules and RNA, although their occurrence frequencies differ among the systems. The GG_mWH_(16)(27) base pair dominates frequencies of other base pairs in RNA (53.48%), but is far less common in crystals of small molecules (13.10%). The GG_mSS_(23)(32) and GG_fWH_(26)(17)(68) base pairs each make up little >20% of all base pairs occurring in RNA, but are far more common for small molecules (27.38% and 55.95% respectively). The last base pair in RNA, GG_mWW_(16)(61), is fairly uncommon both among small molecule crystals (2.38%) and RNA crystals (4.35%). There is also one base pair, GG_fWH_(16)(67), observed in small molecule crystals, but very rarely (only one), and absent in RNA.

For uracil the UU_fWW_(23)(34) base pair makes up 92.31% of all the base pairs in the crystal structures of RNA, but only 3.45% in the crystals of small molecules, which is the largest discrepancy between occurrences in RNA and in small molecules among all the other nucleobases. For the other two base pairs that occur in both systems, UU_fWH_(34)(45) and UU_mWW_(34)(43), the situation is reversed – they are rather frequent in small molecule crystals (51.72% and 21.55% respectively) but far less common in RNA crystals (3.42% and 1.07%). There is one base pair, which is exclusive to RNA crystal structures, namely UU_mWH_(25)(34), and it 3.21% of all RNA base pairs. As for the small molecule crystal structures, there are two base pairs that can be found only among them (UU_mHH_(45)(54), UU_mWW_(23)(32)). These base pairs make up 23.08% of all the base pairs for substituted uracil molecules.

For cytosine there are two base pairs that occur both in crystals of small molecules and in crystal structures of RNA—CC_mWW_(24)(33)(42) and CC_fWH_(24)(35). The CC_mWW_(24)(33)(42) is a common base pair among small molecules (38.60%), but less frequent in RNA (22.58%). The CC_fWH_(24)(35) is also quite common—15.79% and 25.81% respectively. There are four base pairs (CC_mWW(23)(32), CC_mWW_(34)(43), CC_fWS_(26)(31)(42), CC_mSS_(12)(21)) that are only present in small molecule crystals, of which the last two cannot be found among substituted cytosine structures, these make 43.86% of the substituted base pairs. The CC_fWW_(23)(34) base pair is exclusive to RNA structures and makes up 38.71% of base pairs.

### Comparison to geometry families and isostericity matrices found in RNA

Leontis *et al.* (5) discussed geometric families of base pairs and their associated isostericity matrices. One of the objectives of their analysis was to find base pairs that can substitute one another in an RNA structure, while preserving the molecule's 3D structure. After fixing which edges are interacting, the grouping was done on the basis of C1′–C1′ distances.

In our study, we included the averaged C1′–C1′ values for each of the base pair types (Supplementary Tables S.1–6). The C1′–C1′ distances for base pairs occurring in RNA crystal structures and small molecule crystal structures are comparable – in most cases the difference is around 0.5 Å. The biggest difference in C1′–C1′ distances for base pairs in RNA crystal structures and small molecule crystal structures is for the two cytosine base pairs: 0.7 Å for CC_fWH_(24)(35) (which is over two times larger than its uncertainty) and 1.0 Å for CC_mWH_(25)(34) (over three times its uncertainty). As for the rest of the nucleobases and their base pairs, the C1′–C1′ distances from RNA crystal structures fit within the range of uncertainty of small molecule crystal structures. None of the base pairs observed only in small-molecule crystals had the values of C1′–C1′ distances outside the range of the values known for base pairs existing in RNA.

## DISCUSSION

The population of small-molecule crystal structures containing nucleobases is extensive and diverse. Different ways of dividing it can focus on specific features shown by these structures, but such features are usually a result of more than one factor.

### The effect of the absence of a substituent at a nucleobase Sugar edge

Apart from the obvious division by nucleobase type, the first and most important is between crystal structures containing substituted or free nucleobase. For now let us disregard the matter of protonation and discuss the behavior of nucleobases as if we could not determine the positions of hydrogen atoms—a situation very familiar to those working in crystallography on structure determination of biological macromolecules.

The absence of a substituent increases the number of base pairs interacting through the Sugar edge (Figures 2 and 3). There are a few interactions involving Sugar edges in substituted purines, but none for substituted pyrimidines. This is easy to explain if one considers how much ‘space’ is left for the interactions if a substituent blocks the N9 position in purines or N1 in pyrimidines. For pyrimidines the whole edge is completely blocked. For purines there are still atoms no. 2 and 3 left free, which is why we observe interactions involving Sugar edge, in particular the mSS_(23)(32) common for adenine, guanine and hypoxanthine. This interaction is more frequent for substituted guanines than for other purines, which can be explained by the types of atoms that are left free for interactions. For adenine and hypoxanthine there are C2 and N3 left free, whereas for guanine - N2 and N3. As nitrogen is more electronegative than carbon, it will be more prone to form hydrogen bonds. This directly correlates with the frequency of interactions involving Sugar edge being the highest for guanine among all the other nucleobases.

One may think that if the N9 in purines becomes available for interaction, then it would be the main reason for the increase in the number of Sugar edge interactions. This is the case for adenine, but not for guanine or hypoxanthine. This puzzling observation may be explained by comparing the number of structures available for analysis. The number of structures containing adenine is larger than that of guanine or hypoxanthine, which suggests that the features observed for adenine are less biased by small sample size and are more representative of global trends.

### The effect of protonation and charge

Various protonation patterns of nucleobases forming base pairs were observed (Supplementary Figure S.2), all of them were related with the presence or absence of a proton at the nitrogen atoms from purine or pyrimidine rings. Amino groups always had two protons, and carbonyl groups were never protonated. About one third of analyzed nucleobases were charged. In all but one case, the charge of the nucleobase was positive and resulted from protonation of one (rarely two) of the base nitrogen atoms. Adenine, guanine and cytosine formed base pairs with charged nucleobases, but hypoxanthine, thymine and uracil formed only base pairs with neutral molecules.

The protonation is mostly determined by the values of the p*K*_a_ constant(s) and by crystallization conditions. Unfortunately we were unable to take crystallization conditions into consideration as this type of data is not deposited in the CSD on a regular basis, however, analysis of the relation between the p*K*_a_ values and observed phenomena related to protonation was possible to some extent.

The values of p*K*_a_ found in literature (Supplementary Table S.7 in SI1) perfectly explain the differences between the numbers of structures containing neutral and charged nucleobases. Cytosine has the highest p*K*_a_ and the highest fraction of structures with protonated nucleobases (Table 1), followed by adenine, then by .guanine. Looking at hypoxanthine, its p*K*_a_ should not differ much from that of adenine and guanine, yet we found a limited number of structures containing charged hypoxanthine and base pairs with protonated hypoxanthine were not present in any of them. This was most probably caused by the very small number of structures with hypoxanthine deposited in the CSD. As for thymine and uracil, their p*K*_a_ values are the lowest and as such, no structures with protonated uracil or thymine are present.

The experimental p*K*_a_ values are usually given without specifying the protonation or deprotonation site, therefore to explain the preferences in protonation patterns found among the structures, we compared our findings with the theoretical calculations of free energy found in literature (23,30). Halder *et al.* stated that in general ‘The order of site specific protonation (…) is as follows: Cytosine N3 > adenine N1 > guanine N7 > adenine N7 > adenine N3 > guanine N3′, which is in agreement with our findings. Cytosine possesses the largest fraction of protonated structures, adenine is usually protonated at the N1 position, with the N7 and N3 rarely protonated (Supplementary Figure S.2, Tables 2–7). Guanine gets protonated almost exclusively at the N7 position and the fraction of protonated guanines is smaller than the fraction for cytosine or adenine. This is observed for the structures containing base pairs—we did not analyze structures without base pairs.

Typical pKa valuesl for particular types of functional groups may be influenced (30) by numerous factors. Here the most relevant are formal charge of the entire molecule and the presence or absence of a substituent. Apparently, these two factors are not strong enough to make a significant difference in protonation patterns exhibited by the nucleobases—unexpected patterns are generally single occurrences (Supplementary Figure S.2, Tables 2–7).

Protonation patterns should correlate with the ability of a base to form particular types of base pairs and the easiest to spot is the need for protons which participate directly in the formation of hydrogen bonds in a given base pair. There are only a few base pair types whose existence depends on the presence of additional protons (Tables 2–7), for example, GG_fWH_(16)(67), GG_mHH_(67)(76) or CC_mWW_(24)(33)(42). Interestingly, the frequency of occurrence of such bases are some of the lowest, with exception to the CC_mWW_(24)(33)(42) pair.

Another obvious mechanism relating protonation to base pair formation is a steric clash introduced by an added proton. The effect is best seen in adenine and cytosine. For adenine the most common protonation form is protonated N1, which allows N1 to act as a hydrogen bond donor. This prevents the formation of certain pairs but facilitates the formation of others, the former being less frequent. Looking at adenine in its neutral form, one can see that it forms a puzzle piece with each edge containing one proton donor and one acceptor. It should be able to form interactions with another edge that has one proton donor and one acceptor as well. Turning N1 from proton acceptor into donor disrupts this mechanism and makes a puzzle piece with one edge that ‘doesn’t fit’. The Watson-Crick edge, quite common among neutral adenine pairs, almost disappears when it comes to the charged molecules (Table 2, Supplementary Figure S.5 in SI1). In the case of free and charged molecules the chart is almost equally divided between base pairs involving Hoogsteen and Sugar edge.

As for cytosine, the situation is somewhat different from adenine. The presence of an additional proton at N3 does not prevent the Watson–Crick edge, to which this nitrogen atom belongs, from the formation of base pairs (Table 7, Supplementary Figure S.7). This is due to the fact that in the case of cytosine the presence of an additional proton prevents formation of some base pairs, but at the same time enables the ones that possess very similar geometry. This is clearly visible for a series of protonated CC_mWW_(23)(32), semiprotonated CC_mWW_(24)(33)(42) and neutral CC_mWW_(34)(43) base pairs, the last two types being among the most frequent.

There are however, base pair types which can be formed by nucleobases with various protonation patterns yet only some patterns are preferred. Here, deeper analysis of differences in values of p*K*_a_ among available proton donors and acceptors is needed.

It is postulated (33) that the ability to form hydrogen bonds is tightly correlated with the p*K*_a_ of the proton donor and acceptor. The lower the p*K*_a_ value the easier it is for a donor to share the proton, and the more difficult it is for an acceptor to take the proton. If the molecule has more than one proton donor and these donors differ by their p*K*_a_, then the preferred hydrogen bond to form will be with the donor of the lowest p*K*_a_. One may think that this means a hydrogen bond will most likely form between a donor with the lowest p*K*_a_ and an acceptor with the highest p*K*_a_. However it is now quite so simple, and the p*K*_a_ equalization principle explain the phemomenon: ‘the strength of the D–H…A bond increases with decreasing Δp*K*_a_ = p*K*_a_(D–H) – p*K*_a_(A–H+), the difference of acidic constants of the hydrogen bond donor and acceptor, and this strength reaches a maximum when Δp*K*_a_ approaches zero.’ (33).

The appearance of better proton donor or acceptor, or the emergence of the possibility of creating a pair with better balanced p*K*_a_ values, may be partially responsible for observed differences in preferences for forming particular base pair types by the substituted and free nucleobases, or by charged and neutral nucleobases, which cannot be explained simply by the lack or presence of steric clash or the unavailability of a proton donor (Tables 2–7) (Supplementary Figure S.5–7). In adenine, for example, the most frequent pair among substituted adenines is AA_fWH_(16)(67), which is practically not formed by free adenines, although theoretically it could be. Instead, the AA_mSS_(39)(93) pair appears. Evidently, the N6 atom (amine) is a worse proton donor than N9 (imidazole) (34) and the N7 atom (imidazole) is a worse proton acceptor than N3 (pyrimidine). N1 (pyrimidine) seem to be a slightly better proton acceptor than N3 (pyrimidine).

Finally, there are cases when there does not seem to be any particular preference and a base pair is more or less equally formed by various protonation or substitution states of nucleobases. The most prominent case is AA_mHH_(67)(76) which is formed by substitutes (both neutral and charged) with similar inclination. The CC_mSS_(12)(21) is an analogous case but for free cytosine—there is not much of a change in numbers between neutral, protonated or semi-protonated nucleobases forming this base pair. The third base which somehow fits to this category is the GG_mSS_(23)(32) pair. The base prefers the neutral form if substituted, but the protonated one if free.

The last remark related to the effect of charge regards the length of hydrogen bonds. It would feel natural for the hydrogen bonds to be longer if they are formed between identically charged molecules as two molecules of the same charge should repulse each other. However, in our study we did not find proof for this. Unfortunately, in most cases it was a matter of the sample being too small to draw a statistically significant conclusion. Surprisingly when samples were big enough, we found no differences in hydrogen bond lengths between charged and neutral base pairs. This may be related with the fact that the base pairs are not in the gas phase but are part of a crystal in which counterions may balance same charge repulsion (1,2).

### Searching for a relation between the frequency of occurrence and energy of intermolecular interaction

In our investigations, we aimed to compare the frequencies of occurrences of base pairs in small molecule crystals with intermolecular interaction energies calculated for the base pairs by means of quantum mechanics available in the literature (35–44) (Supplementary Tables S.8–12 in the SI1). Unfortunately, the available data does not permit is to draw any conclusions. Energies are calculated for only a few of type of base pairs, with different methodology and mostly for free nucleobases only.

However, on the basis of this scarce energy data, a positive correlation between the calculated energy and the type and number of hydrogen bonds forming the base pair can be observed. Following the generally accepted (23) order of hydrogen bond strength as N–H…O > N–H…N >> C–H…O > C–H…N it can be concluded from Supplementary Tables S.8–12 that the presence of stronger and more numerous hydrogen bonds result in more favorable base pair energy.

Our analysis shows that there does not seem to be any relationship between the type and number of hydrogen bonds and frequency of particular base pairs. Whether we consider the whole set of base pairs for each nucleobase without division between substituted, free, charged or neutral nucleobases, or we make the division, the correlation between type and number of hydrogen bonds and the frequency of base pairs seem to be random.

For cytosine, for example, it is true that the three most common base pairs (CC_mWW_(24)(33)(42), CC_mWW_(34)(43) and CC_mSS_(12)(21)) are comprised of rather strong hydrogen bonds, especially the most common CC_mWW_(24)(33)(42), which has three, not two bonds. The situation is different for guanine – one might think that the most common base pairs should be GG_mWW_(16)(61) and GG_fWH_(16)(67) as they both are formed by strong N–H…O hydrogen bonds, yet the complete opposite is true and these base pairs are one of the rarest. It is true that the most common base pair is formed by three bonds (GG_fWH_(26)(17)(68)), but at the same time the geometry of the third bond is on the very edge of allowing it to be considered a hydrogen bond. It is difficult to assess the influence of types of bonds on thymine, as there is only one base pair (and a single structure) that has different type of hydrogen bond than N–H…O, namely TT_fWH_(34)(45). If we look at uracil, then we may think that, similarly to thymine, most of the base pairs should be formed by N–H…O bonds, yet it turns out not to be true. The most common base pair is the ‘Calcutta’ pair (UU_fWH_(34)(45)) (45,46), and is formed by N–H…O and C–H…O bonds. This base pair is frequently studied thanks to its biological importance, but it is hard to imagine that this importance would be enough for the crystallographers to populate the CSD with sixty unique structures containing it.

From this lack of consensus, a conclusion can be drawn—the process of base pair formation is complex and influenced by many factors like presence of additional molecules or the ability for nucleobases to form more elaborate, higher dimensional structures (like ribbons, layers or stacked columns). This is why the attempts to explain frequencies of occurrence of particular base pairs by calculating and approximating their interaction energies are prone to mistakes. Some nucleobases may behave with accordance to the energies, but it is not the majority.

It would be ideal if we could include the crystallization energy for found structures, and correlate it with frequencies of occurrence. Unfortunately such energies are not easy to obtain. Calculation of crystallization energy or experimental estimation of it is not trivial and therefore not a routine part of structure solution and refinement, and as such is not deposited in the CSD nor found in corresponding publications.

### The effect of nucleic acid backbone

We have shown that the presence of a substituent at the position of glycosidic bond in nucleic acids does change the frequency of occurrence of particular types of base pairs. To see the influence of polymeric chain binding the nucleobases in RNA crystal structures, we will now discuss the comparison of frequence of occurrence of substituted only base pairs present in small molecules crystal structures with base pairs present in RNA crystal structures.

The differences in frequencies of occurrence of base pairs between substituted nucleobases found in small molecules crystal structures and crystal structures of RNA (3,6) vary among nucleobases. Here, we wish to stress again that we are comparing small molecule crystal structures with substituted base pairs, and RNA crystal structures with base pairs fitting the criteria as described in ‘Search for base pairs in the CSD’, so the base pairs interacting through the sugar ring are excluded.

For each nucleobase, most of the base pairs are found in both systems, which is not surprising considering that there is a limited number of ways the base pair can be formed. If we order the base pairs by how frequently they appear in each system, we see that the top three most frequent base pairs are the same in most of the cases, but their order is different. Such behavior is rather surprising as we are comparing base pairs in different environments (small molecule crystal and RNA) but choosing similar molecules (substituted nucleobases as opposed to comparison to free ones or a summary of both).

The number of base pair types unique to small molecule crystal structures is slightly larger than the ones found only in RNA crystal structures. This is true to both purines and pyrimidines. It can be explained by the differences in the environment—in small molecule crystal structures the molecules have more freedom to form base pairs than they have while restricted by the RNA sugar-phosphate backbone.

We tried to investigate if the frequency of base pairs in small molecule crystal structures and RNA crystal structures is somehow linked to their symmetry, relative orientation of substituents or ability to form higher dimensional assemblies. We searched if base pairs with rotation symmetry (like mSS_(23)(32) or mWW_(34)(43)) or base pairs that are able to form a ‘tape’ of base pairs (like fWH_(16)(67) or fWH_(34)(45)) are more common among small molecule crystal structures, than in RNA crystal structures. It seemed probable that such base pairs would pack better and therefore be favored in the small molecule crystal structure, but would lose this advantage in RNA structures. At the same time, we tried to see if base pairs with substituents arranged in such a way that they may fit into an RNA strand are favored among RNA structures. Of course it is easy to think about a base pair that seems to be advantageous in both environments, so we took this factor into account. For now, there does not seem to be much correlation between the base pair geometry and its preference to be more common in one of the environments (small molecule crystal structures or RNA crystal structures). Other factors most probably outweigh the advantages of more symmetrical geometry enabling better packing or fitting into an RNA strand.

### The atlas as an aid in RNA structure solution and analysis

Many base pairs observed in small molecule crystals are protonated, and this may also be true for some base pairs in RNA structures. This is even more likely when the presence of the negatively charged backbone is taken into account, which may compensate for positive charge of protonated bases. Knowledge regarding the preferences for formation of particular pair types exhibited by neutral and charged bases can be useful in RNA crystal structure solution. RNA crystal structures often do not permit unequivocal identification of each base pair and require making an educated guess as to which base pair may be formed. Knowing the differences in preferences between neutral and charged molecules may help in making more accurate predictions In addition, there is no statistically significant difference in the hydrogen bond lengths for neutral and protonated base pairs, therefore the bond length and C1′–C1′ distance cannot be an accurate indicator for base pair charge.

Another matter is metastable pairs of charged nucleobases (2). Our study shows that such base pairs are more common than a scientist would think (like metastable AA_mHH_(67)(76) (19), second most common adenine base pair). Metastable base pairs may serve as a pH dependent switch. They may form in lower (protonated base pairs) or higher (neutral base pairs) pH, be moved into different pH that does not facilitate their formation, but does not ‘break’ them either. Then such base pairs may exist but be very fragile (metastable) and break apart easily with a release of energy (19).

## CONCLUSIONS

This survey of small molecule crystal structures containing nucleobase hydrogen bonded pairs provides interesting insight into nucleobase pair properties. Many of the base pairs can be found both among small molecule crystals and in RNA crystal structures—it is interesting that the most frequent base pairs are found in both systems, although the frequency of particular base pairs differs. Obviously there are occurrences of base pairs that are unique for small molecules crystal structures or for RNA crystal structures, but such instances are rare. One may conclude that the geometry of the most frequent base pairs results from properties intrinsic to a particular base rather than from the external forces.

We compared the frequencies of occurrence of particular base pairs with the interaction energies calculated by the means of quantum mechanics and found many publications with such data, but none of them had values for all the base pairs found in crystal structures.

Although p*K*_a_ values can be influenced by substituents and external conditions, we found that most of the nucleobases forming base pairs were protonated in their most probable way. In most cases the protonation heavily influenced the type of base pair that was formed for various reasons. Sometimes the presence of the additional proton prevented the base pair from forming, and in other cases the additional proton was necessary to form it. Surprisingly there were many cases in which the protonation was not needed to form the base pair, yet the pairs displayed strong preference for a particular protonation. We found that base pairs formed from differently protonated molecules are also possible, but not so common.

Our survey included commentary on the average bond lengths of various hydrogen bonds formed in observed types of base pairs. Interestingly, we did not find any statistically significant differences between the bond lengths of two positively charged molecules and their neutral versions. This may be due to this difference being minimal despite the obvious fact that the electrostatic repulsive forces are rather strong, or the number of investigated structures being too small to give significant results.

The similarity of C1′–C1′ distances between RNA crystal structures and small molecule crystal structures prove that these distances are determined not by the sugar-phosphate backbone of RNA but rather by the nucleobases themselves.

While there are many general conclusions applicable to all the base pairs, we found that despite being closely related, each nucleobase expresses some characteristic behavior that may at first seem puzzling. All these puzzling behaviors can be explained by considering additional factors like the presence or absence of a substituent at the N1/N9 position, the protonation pattern, the geometry of the base pair and its ability to form more complex structures. Especially the last element seems to have a large impact on the frequency of occurrence of base pairs – for both comparisons with RNA structures and values of dimer interaction energies the differences could be logically attributed to the ability of particular base pairs to pack in certain ways in a crystal and form additional interactions. It is clear that a thorough analysis of more complex structures formed by base pairs is an inevitable next step in order to deepen our understanding of base pair interactions.

## DATA AVAILABILITY

Original data were retrieved from CCDC ConQuest version 1.23, database version 5.39 (August 2018). Detailed data regarding data analysis is available from the authors upon request.

## Supplementary Material

gkaa629_Supplemental_Files
