# Supplementary material for: Frequency and hydrogen bonding of nucleobase homopairs in small molecule crystals
Source: Nucleic Acids Res. 2020 Jul 29;48(15):8302–19. doi: 10.1093/nar/gkaa629 (PMC7470937; doi:10.1093/nar/gkaa629)
Supplement: gkaa629_Supplemental_Files [file gkaa629_supplemental_files.zip › Supplementary1_20200710.pdf]

Frequency and hydrogen bonding of nucleobase homopairs in small molecule crystals – supplementary data

Małgorzata Katarzyna Cabaj[1] and Paulina Maria Dominiak[1,]*

[1] Biological and Chemical Research Center, Department of Chemistry, University of Warsaw, ul. Żwirki i Wigury 101, 02-089 Warszawa, Poland

[*] To whom correspondence should be addressed. Tel: +48 22 55 26 714; Fax: not applicable; Email: pdomin@chem.uw.edu.pl

ORCID

Małgorzata Katarzyna Cabaj: 0000-0001-9184-4513

Paulina Maria Dominiak: 0000-0002-1466-1243

Keywords

nucleobase pairs, hydrogen bonds, small molecule crystals, nucleobases protonation

Key points

1. Frequency of occurrence of nucleobase pairs in small molecule crystal structures
2. Hydrogen bond geometry of nucleobase pairs in small molecule crystal structures
3. Protonation of nucleobase pairs in small molecule crystal structures

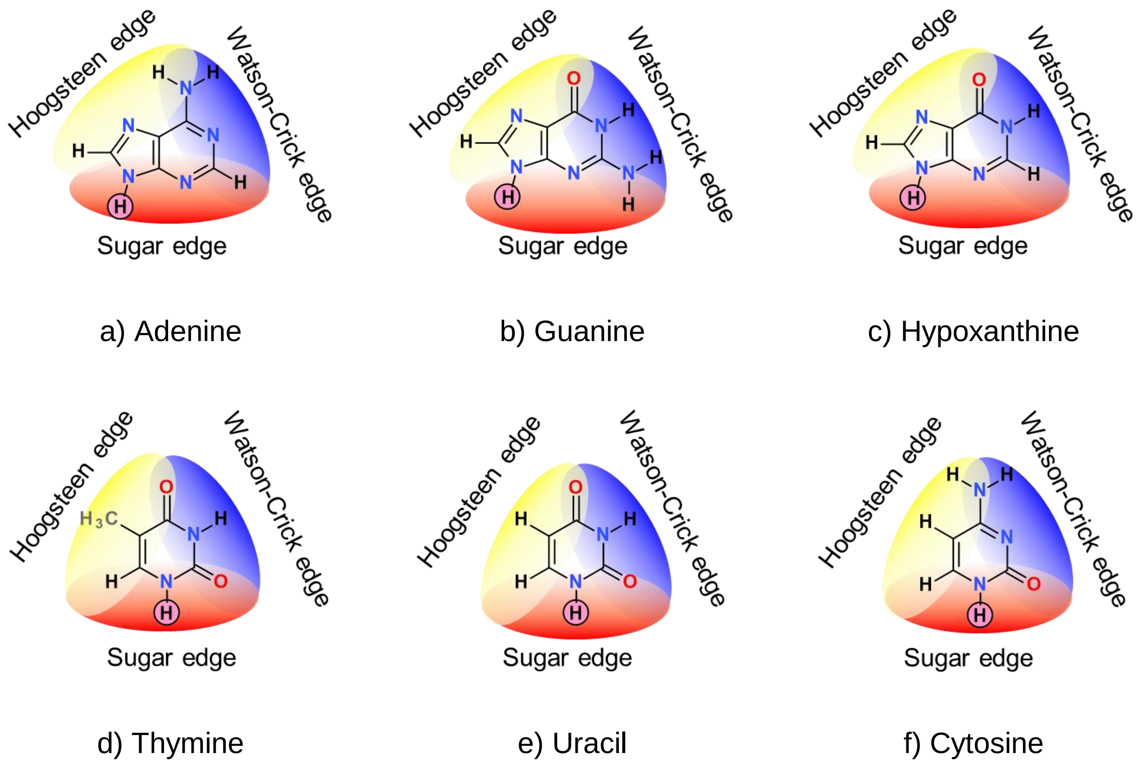

Figure S.1 Naming scheme for the edges of investigated nucleobases. The particular edges are color coded in the same way as in the rest of the publication – Watson-Crick edge is blue, Hoogsteen edge is yellow, and Sugar edge - red.

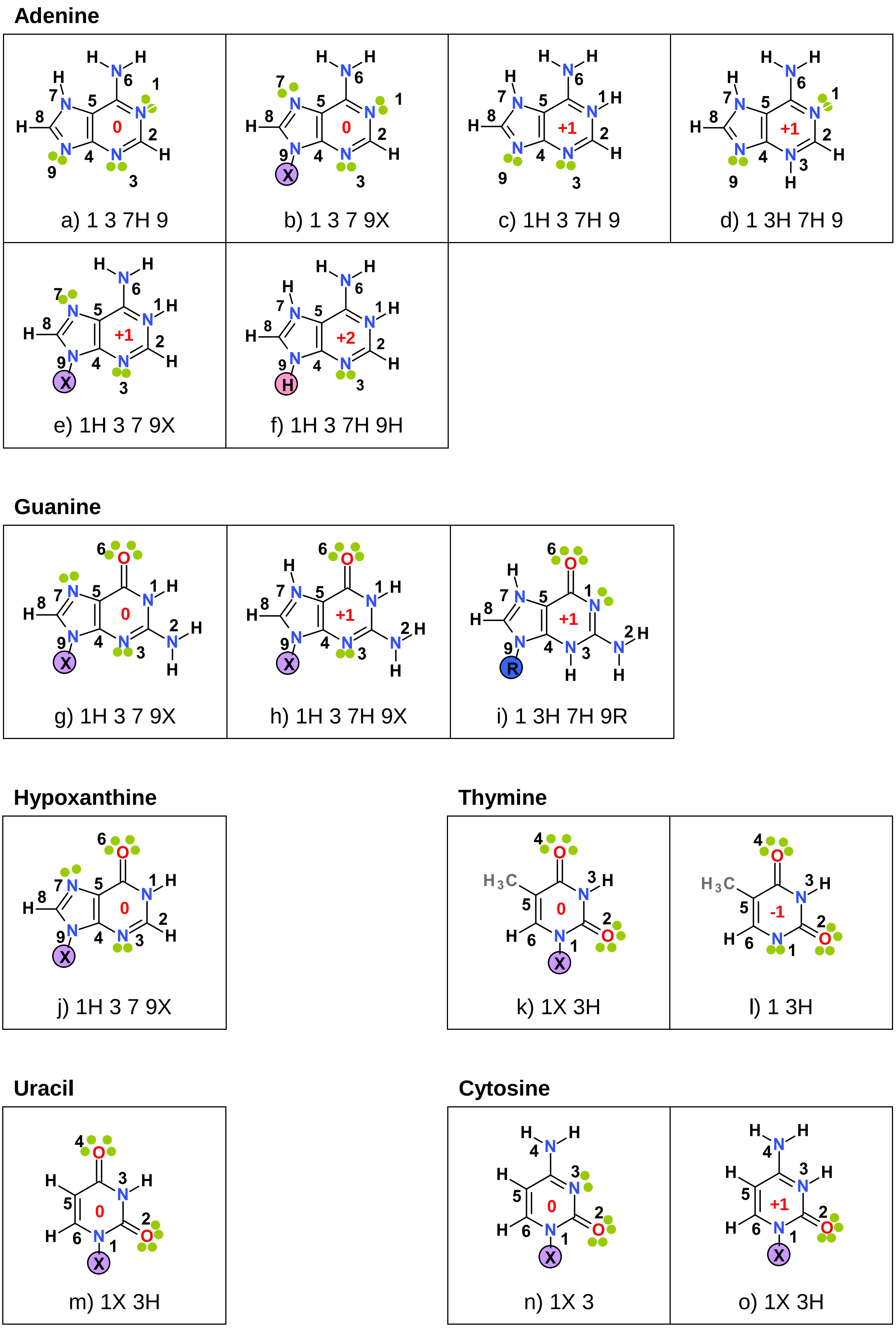

Figure S.2 Atom naming scheme for the nucleobases with the protonation patterns observed among the investigated structures containing base pairs.

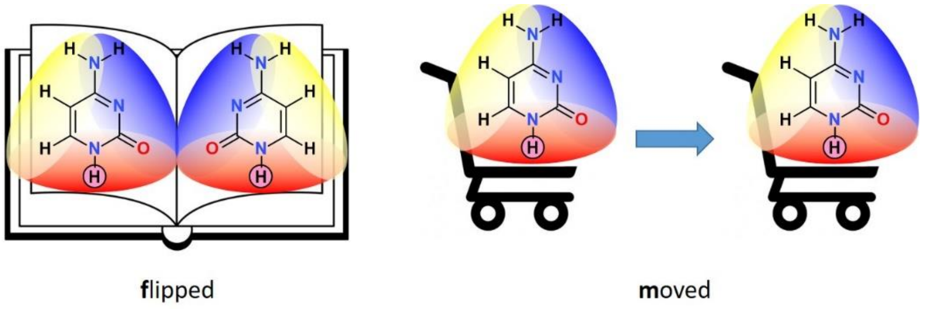

Figure S.3 Examples of flipped and moved orientation of the base pairs.

Naming of hetero base pairs

Although the number of structures containing base pairs formed by different types of nucleobases deposited in CSD is too small to make any statistically significant conclusions, and subsequently will not be a subject of this analysis, we find it useful to present a base pair naming scheme for hetero-base pairs too. If we consider hypothetical base pair in Figure S.4, then the naming is very similar to the one for homo base pairs. To make the naming explicit:

(1) We order the nucleobases letter symbols firstly by type of a molecule (purine first, pyrimidine second) and for the same type of molecule - alphabetically, so in this particular example adenine will be the first, and guanine the second - AG.

(2) Next, we need to decide whether these two bases are in flipped or moved relative orientation. For hetero-base pair this is not as straightforward as for homo-base pairs. In homo-base pairs, moved orientation means that bases present the same side of the ring(s) to the observer, flipped – different sides. In hetero-base pairs, one first has to decide what “the same side” means. Here the relative orientation of three different edges, Watson-Crick, Hoogsteen and Sugar Edge, is decisive. The observer can start from Watson-Crick edge and check which edge is next when going counterclockwise. If for each base the next edge is different, the bases are presenting different sides and are in “flipped” relative orientation. If both bases next edge is the same, the bases are presenting the same side and are in “moved” relative orientation. This is the case for exemplary base in Figure S.4.

(3) Adenine interacts with Hoogsteen Edge, and guanine - with Watson-Crick Edge. Here we cannot order the edges by their order of importance, as it would make it confusing to discern which nucleobase interact with which edge, therefore we write them in the order of nucleobases - HW.

(4) Then we number the interacting atoms and put each hydrogen bond inside a parenthesis, starting from the smallest number from the first nucleobase. In this example, adenine is interacting via N6 and N7, and guanine - via N1 and O6. The N6-H6…O6 and N7…H1-N1 hydrogen bonds are formed. We have to start from adenine, so the numbers in parenthesis will look as follows: (61)(76).

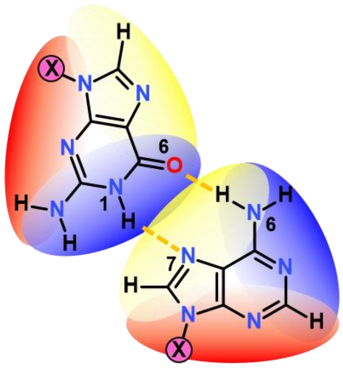

Figure S.4 Example of a hypothetical hetero-base pair - AG_mHW_(61)(76).

Another example could be the canonical AT base pair, which according to our nomenclature will be named AT_fWW_(13)(64).

Table S.1 Table presents the average lengths of hydrogen bonds present in adenine base pairs illustrated in Figure 4 in the main text. N_{all} column tells how many separate entries there were in CSD for particular base pair. N_H is the number of structures with hydrogen positions determined. N_{uniq} is the number of unique structures present in CSD, as they are represented in pie charts in Figure 3.a in the main text. The D-H bond lengths were adjusted to their neutron lengths to compensate for the underestimate of the X-ray methods.

	**D-H…A**	**D…A [Å]**	**D-H [Å]**	**H…A [Å]**	**D-H…A [º]**	**C1'…C1' [Å]**	**N_{all}**	**N_H**	**N_{uniq}**
AA_mWW_(12)(21)	C2-H2…N1	3.9 (2)	1.089	3.0 (2)	145 (16)	11.9 (4)	18	16	16
AA_fWW_(16)(21)	N6-H6…N1	3.214	1.015	2.201	175	12.000	1	1	1
	C2-H2…N1	3.251	1.089	2.316	143				
AA_mWW_(16)(61)	N6-H6…N1	3.06 (16)	1.015	2.1 (3)	166 (17)	13.75 (12)	45	41	43
AA_fWH_(18)(27)	C8-H8…N1	3.61 (15)	1.089	2.7 (2)	142 (17)	9.97 (14)	4	4	3
	C2-H2…N7	3.83 (17)	1.089	3.03 (13)	130 (5)				
AA_fWH_(16)(67)	N6-H6…N1	2.97 (6)	1.015	2.01 (6)	158 (7)	12.33 (9)	152	144	106
	N6-H6…N7	3.04 (6)	1.015	2.1 (1)	170 (7)				
AA_fWS_(19)(63)	N1-H1…N9	2.92 (8)	1.015	1.77 (2)	171 (4)	-	4	4	3
	N6-H6…N3	2.82 (8)	1.015	1.95 (4)	174 (2)				
AA_mHH_(67)(76)	N6-H6…N7	3.0 (1)	1.015	2.0 (2)	158 (9)	11.1 (3)	97	78	86
AA_fHH_(67)(78)	N6-H6…N7	3.194 (8)	1.015	2.34 (2)	142 (4)	8.5 (1)	2	2	2
	C8-H8…N7	3.81 (7)	1.089	2.89 (7)	144 (2)				
AA_mHH_(78)(87)	C8-H8…N7	3.7 (2)	1.089	2.90 (18)	127 (5)	8.0 (3)	23	22	15
AA_fHS_(63)(72)	N6-H6…N3	3.02 (5)	1.015	2.02 (5)	168 (0)	9.447 (5)	2	2	2
	C2-H2…N7	3.26 (3)	1.015	2.30 (1)	145 (3)				
AA_mHS_(63)(79)	N6-H6…N3	2.935	1.015	1.937	167	-	1	1	1
	N9-H9…N7	2.888	1.015	1.882	171				
AA_mHS_(72)(83)	C2-H2…N7	3.5 (2)	1.089	2.7 (3)	130 (13)	7.1 (3)	11	11	9
	C8-H8…N3	3.9 (2)	1.089	3.3 (1)	118 (8)				
AA_fHS_(79)(83)	N9-H9…N7	2.921	1.015	1.914	171	-	1	1	1
	C8-H8…N3	3.608	1.089	2.677	143				
AA_mSS_(23)(32)	C2-H2…N3	3.5 (3)	1.089	2.8 (3)	126 (11)	8.9 (8)	22	16	15
AA_mSS_(39)(93)	N9-H9…N3	2.90 (7)	1.015	1.92 (8)	162 (5)	-	23	22	21
AA_mSS_(39)(93)	N3-H3…N9*	2.808	1.015	1.826	162	-	1	1	1

Table S.2 Table presents the average lengths of hydrogen bonds present in guanine base pairs illustrated in Figure 5 in the main text. N_{all} column tells how many separate entries there were in CSD for particular base pair. N_H is the number of structures with hydrogen positions determined. N_{uniq} is the number of unique structures present in CSD, as they are represented in pie charts in Figure 3.e in the main text. The D-H bond lengths were adjusted to their neutron lengths to compensate for the underestimate of the X-ray methods.

	**D-H…A**	**D…A [Å]**	**D-H [Å]**	**H…A [Å]**	**D-H…A [º]**	**C1'…C1' [Å]**	N_{all}	N_H	N_{uniq}
GG_mWW_(16)(61)	N1-H1…O6	2.79 (3)	1.015	1.80 (4)	164 (3)	13.39 (7)	2	2	2
GG_mWH_(16)(27)	N1-H1…O6	2.88 (4)	1.015	1.91 (4)	167 (5)	11.59 (9)	47	47	11
	N2-H2…N7	2.91 (5)	1.015	1.92 (5)	161 (3)				
GG_fWH_(16)(67)	N1-H1…O6	2.799	1.015	1.804	166	12.050	1	1	1
	N7-H7…O6	2.687	1.015	1.678	173				
GG_fWH_(26)(17)(68)	N2-H2…O6	2.90 (7)	1.015	1.9 (1)	155 (9)	11.2 (1)	77	74	48
	N1-H1…N7	2.84 (4)	1.015	1.84 (4)	169 (6)				
	C8-H8…O6	3.4 (1)	1.089	2.8 (2)	118 (6)				
GG_fWH_(26)(17)(68)	N2-H2…O6	2.859 (2)	1.015	1.919 (1)	152.6 (4)		1	1	1
	N7-H7…N1*	2.827 (4)	1.015	1.828 (5)	167.48 (7)				
	C8-H8…O6	3.52 (1)	1.089	2.85 (2)	119 (1)				
GG_mHH_(67)(76)	N7-H7… N6	2.8 (3)	1.015	1.71 (3)	162 (4)	-	10	8	7
GG_mSS_(23)(32)	N2-H2…N3	3.04 (6)	1.015	2.9 (8)	172 (7)	8.2 (2)	48	44	33
GG_mSS_(39)(93)	N9-H9…N3	2.892	1.015	1.888	170	-	1	1	1

Table S.3 Table presents the average lengths of hydrogen bonds present in hypoxanthine base pairs illustrated in Figure 6 in the main text. N_{all} column tells how many separate entries there were in CSD for particular base pair. N_H is the number of structures with hydrogen positions determined. N_{uniq} is the number of unique structures present in CSD, as they are represented in pie charts in Figure 3.i in the main text. The D-H bond lengths were adjusted to their neutron lengths to compensate for the underestimate of the X-ray methods.

	**D-H…A**	**D…A [Å]**	**D-H [Å]**	**H…A [Å]**	**D-H…A [º]**	**C1'…C1' [Å]**	**N_{all}**	**N_H**	**N_{uniq}**
HxHx_mWW_(16)(61)	N1-H1…O6	2.785	1.015	1.778 (4)	171 (3)	-	3	3	1
HxHx_fWH_(17)(26)	N1-H1…N7	2.85 (7)	1.015	1.90 (8)	165 (8)	11.37 (9)	18	13	9
	C2-H2…O6	3.28 (21)	1.089	2.39 (19)	137 (15)				
HxHx_fWS_(13)(62)	N1-H1…N3	3.085	1.015	2.183	147	8.269	1	1	1
	C2-H2…O6	3.145	1.089	2.313	132				
HxHx_fHS_(68)(79)	C8-H8…O6	3.170 (17)	1.089	2.33 (4)	133 (3)	-	3	3	1
	N9-H9…N7	2.810 (8)	1.015	1.822 (11)	164 (4)				
HxHx_mSS_(23)(32)	C2-H2…N3	3.35 (4)	1.089	2.48 (6)	136 (4)	8.44 (1)	4	4	2

Table S.4 Table presents the average lengths of hydrogen bonds present in thymine base pairs illustrated in Figure 7 in the main text. N_{all} column tells how many separate entries there were in CSD for particular base pair. N_H is the number of structures with hydrogen positions determined. N_{uniq} is the number of unique structures present in CSD, as they are represented in pie charts in Figure 4.a in the main. The D-H bond lengths were adjusted to their neutron lengths to compensate for the underestimate of the X-ray methods.

	**D-H…A**	**D…A [Å]**	**D-H [Å]**	**H…A [Å]**	**D-H…A [º]**	**C1'…C1' [Å]**	**N_{all}**	**N_H**	**N_{uniq}**
TT_mWW_(23)(32)	N3-H3…O2	2.84 (5)	1.015	1.84 (5)	170 (6)	8.60 (15)	86	75	66
TT_fWW_(23)(34)	N3-H3…O2	2.84 (6)	1.015	1.86 (8)	170 (5)	8.49 (18)	6	6	6
	N3-H3…O4	2.85 (6)	1.015	1.85 (7)	170 (4)				
TT_mWW_(34)(43)	N3-H3…O4	2.87 (17)	1.015	1.84 (7)	171 (5)	11.04 (14)	72	71	59
TT_fWH_(34)(45)	N3-H3…O4	3.001	1.015	2.005	167	11.56	1	1	1
	C5-H5…O4	3.175	1.089	2.975	124				
TT_fWS_(21)(32)	N1-H1…O2	2.83 (3)	1.015	1.84 (2)	171 (10)	-	6	5	5
	N3-H3…O2	2.82 (1)	1.015	1.813 (9)	173 (3)				
TT_mSS_(12)(21)	N1-H1…O2	2.828 (13)	1.015	1.823 (16)	170 (2)	-	7	7	5

Table S.5 Table presents the average lengths of hydrogen bonds present in uracil base pairs illustrated in Figure 8 in the main text. N_{all} column tells how many separate entries there were in CSD for particular base pair. N_H is the number of structures with hydrogen positions determined. N_{uniq} is the number of unique structures present in CSD, as they are represented in pie charts in Figure 4.d in the main text. The D-H bond lengths were adjusted to their neutron lengths to compensate for the underestimate of the X-ray methods.

	**D-H…A**	**D…A [Å]**	**D-H [Å]**	**H…A [Å]**	**D-H…A [º]**	**C1'…C1' [Å]**	**N_{all}**	**N_H**	**N_{uniq}**
UU_mWW_(23)(32)	N3-H3…O2	2.85 (4)	1.015	1.86 (5)	167 (6)	8.58 (8)	21	20	19
UU_fWW_(23)(34)	N3-H3…O2	2.92 (11)	1.015	1.87 (6)	168 (6)	8.49 (11)	4	3	4
	N3-H3…O4	2.89 (8)	1.015	1.85 (7)	164 (7)				
UU_mWW_(34)(43)	N3-H3…O4	2.84 (4)	1.015	1.84 (4)	170 (6)	11.04 (15)	31	31	26
UU_fWH_(34)(45)	N3-H3…O4	2.81 (5)	1.015	1.83 (7)	143 (9)	11.32 (17)	72	63	60
	C5-H5…O4	3.32 (12)	1.089	2.39 (16)	168 (7)				
UU_mHH_(45)(54)	C5-H5…O4	3.5 (3)	1.089	2.5 (3)	153 (18)	11.5 (4)	9	9	9
UU_mHS_(41)(52)	N1-H1…O4	2.864	1.015	1.858	171	-	1	1	1
	C5-H5…O2	3.320	1.089	2.268	161.71				
UU_mSS_(12)(21)	N1-H1…O2	2.845	1.015	1.844	168	-	1	1	1

Table S.6 Table presents the average lengths of hydrogen bonds present in cytosine base pairs illustrated in Figure 9 in the main text. N_{all} column tells how many separate entries there were in CSD for particular base pair. N_H is the number of structures with hydrogen positions determined. N_{uniq} is the number of unique structures present in CSD, as they are represented in pie charts in Figure 4.h in the main text. The D-H bond lengths were adjusted to their neutron lengths to compensate for the underestimate of the X-ray methods.

	**D-H…A**	**D…A [Å]**	**D-H [Å]**	**H…A [Å]**	**D-H…A [º]**	**C1-C1 [Å]**	**N_{all}**	**N_H**	**N_{uniq}**
CC_mWW_(23)(32)	N3-H3…O2	2.77 (3)	1.015	1.78 (4)	163 (3)	8.526	2	2	2
CC_mWW_(24)(33)(42)	N4-H4…O2	2.84 (5)	1.015	1.84 (7)	172 (9)	9.53 (9)	105	96	62
	N3-H3…N3	2.82 (3)							
CC_mWW_(34)(43)	N4-H4…N3	3.00 (4)	1.015	2.01 (5)	169 (8)	10.96 (17)	27	26	24
CC_fWH_(24)(35)	N4-H4…O2	3.0 (2)	1.015	2.8 (3)	151 (18)	11.0 (2)	11	11	9
	C5-H5…N3	3.8 (3)	1.089	2.2 (4)	149 (12)				
CC_mWH_(25)(34)	C5-H5…O2	3.489	1.089	2.448	159	8.996	1	1	1
	N4-H4…N3	3.017	1.015	2.006	174				
CC_fWS_(26)(31)(42)	C6-H6…O2	3.7 (2)	1.089	2.95 (16)	127 (3)	-	14	14	3
	N1-H1…N3	2.89 (7)	1.015	1.88 (6)	172 (7)				
	N4-H4…O2	3.00 (5)	1.015	2.00 (5)	172 (4)				
CC_mSS_(12)(21)	N1-H1…O2	2.82 (2)	1.015	1.81 (3)	173 (4)	-	26	24	18

Adenine

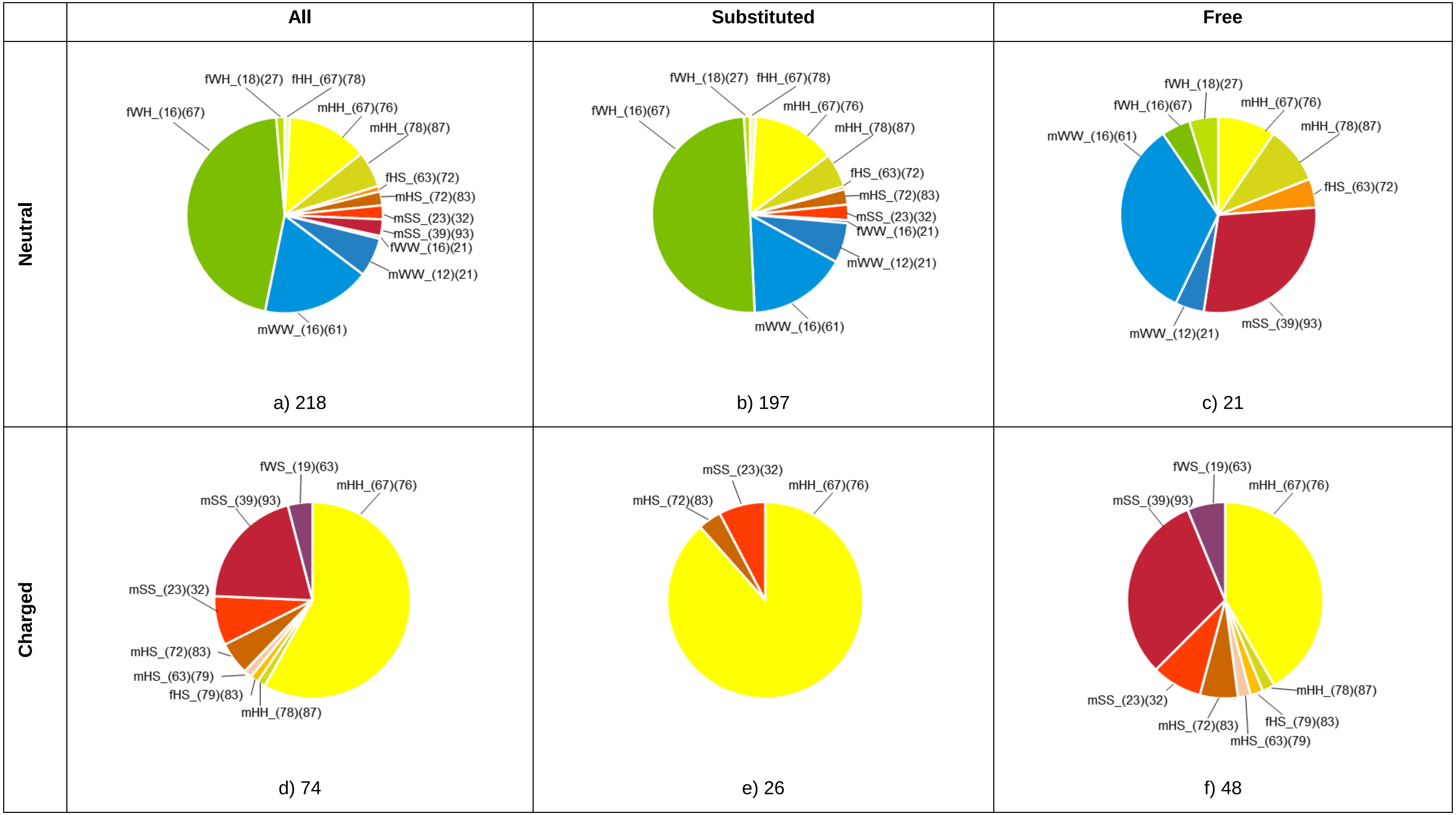

Figure S.5 Frequencies of occurrence of base pairs for adenine with partition into charged and neutral state. The structures without the hydrogen positions determined were not included.

Guanine

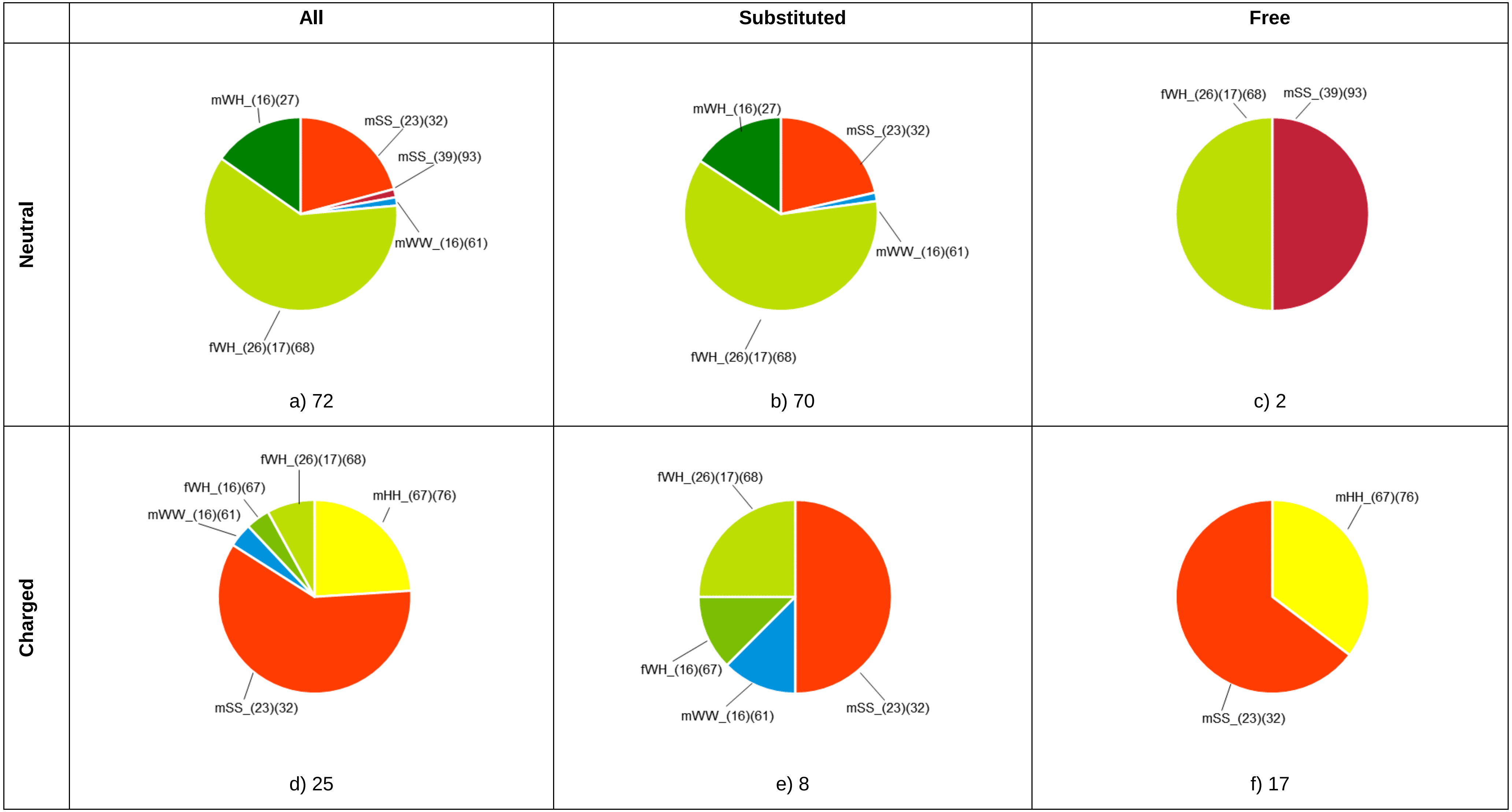

Figure S.6 Frequencies of occurrence of base pairs for guanine with partition into charged and neutral state. The structures without the hydrogen positions determined were not included.

Cytosine

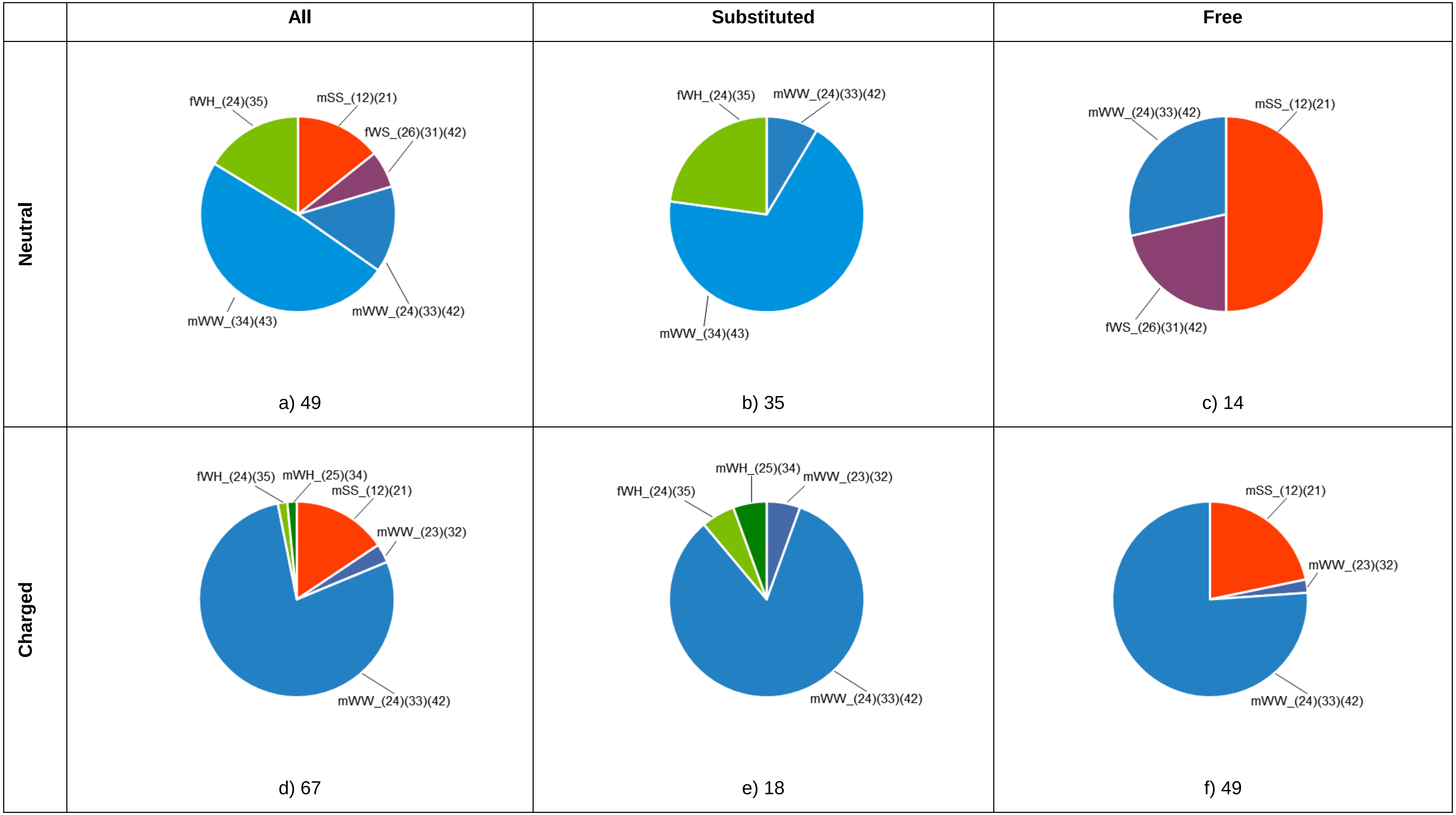

Figure S.7 Frequencies of occurrence of base pairs for cytosine with partition into charged and neutral state. The structures without the hydrogen positions determined were not included.

Table S.7 Summary of pK_a values for nitrogen atoms in nucleobases.

		Wilcox et al., 2011 (1)	Thaplyal et al., 2014 (2)	Krishnamurthy et al., 2013 (3)	Schnabl et al., 2010 (4)
Adenine	N1	3.5	3.5		3.8
	N3				
	N7				
	N9				
Guanine	N1	9.2	9.2	9.5	9.4
	N3				
	N7	2.2	1.6		
	N9				
Hypoxanthine	N1				
	N3				
	N7				
	N9				
Thymine	N1				
	N3			9.8	
Uracil	N1				
	N3	9.2	9.2		9.3
Cytosine	N1				
	N3	4.2	4.2	4.1	4.3

Table S.8 The table below summarizes either stabilization energies (E_{stab}) or total energies (E_{tot}) of adenine. The energies are given in kcal/mol.

**Adenine**	**D-H…A**	**E_{stab}** (5)	**E_{stab}** (5)	**E_{stab}** (6)	**E_{stab}** (7)	**E_{stab}** (8)	**E_{stab}** (9)	**E_{stab}** (10)	**$E_{tot, est}$** (11)
AA_mWW_(16)(21)	N6-H6…N1	-6.0	-6.7						
	C2-H2…N1								
AA_tWW_(12)(21)	C2-H2…N1	-1.4	-2.1	0.5					
AA_tWW_(16)(61)	N6-H6…N1	-13.4	-13.8	-4.4	-14.8	-3.2	-11.8	-8.7	-13.5
AA_mWH_(16)(67)	N6-H6…N1	-12.5	-13.1				-11.1	-8.0	-12.8
	N6-H6…N7								
AA_mWH_(18)(27)	C8-H8…N1	-2.1	-2.8						
	C2-H2…N7								
AA_mWS_(19)(63)	N1-H1…N9	-16.1	-16.6			-4.2			
	N6-H6…N3								
AA_mHH_(67)(78)	N6-H6…N7	-7.6	-8.1						
	C8-H8…N7								
AA_tHH_(67)(76)	N6-H6…N7	-10.8	-11.3	-6.2	-11.8	-3.0	-9.5	-6.9	-11.7
AA_tHH_(78)(87)	C8-H8…N7	-3.9	-4.2	-0.7					
AA_mHS_(63)(72)	N6-H6…N3	-5.8	-6.9						
	C2-H2…N7								
AA_mHS_(79)(83)	N9-H9…N7	-9.9	-10.8						
	C8-H8…N3								
AA_tHS_(63)(79)	N6-H6…N3	-15.2	-15.9			-3.9			
	N9-H9…N7								
AA_tHS_(72)(83)	C2-H2…N7	-2.3	-0.9						
	C8-H8…N3								
AA_tSS_(23)(32)	C2-H2…N3	-1.4	-2.3	-0.7					
AA_tSS_(39)(93)	N9-H9…N3	-19.8	-18.2	-10.1	-14.3	-7.8			

Table S.9 The table below summarizes either stabilization energies (E_{stab}) or total energies (E_{tot}) of guanine. The energies are given in kcal/mol.

**Guanine**	**D-H…A**	E_{stab} (12)	E_{stab} (9)	E_{stab} (13)	E_{stab} (8)	E_{stab} (10)	$E_{tot, est}$ (11)
GG_tWW_(16)(61)	N1-H1…O6	-25.8		-22.1	-10.6	-25.0	-24.7
GG_mWH_(16)(67)	N1-H1… O6						
	N7-H7…O6						
GG_mWH_(26)(17)(68)	N2-H2…O6	-17.3	-18.4	-17.1	-8.1		
	N1-H1…N7						
	C8-H8…O6						
GG_tWH_(16)(27)	N1-H1…O6	-12.0			-2.5	-16.8	-19.5
	N2-H2…N7						
GG_tHH_(67)(76)	N7-H7… N6						
GG_tSS_(23)(32)	N2-H2…N3	-9.0			-4.6	-7.3	-11.8
GG_tSS_(39)(93)	N9-H9…N3	-12.0			-5.1		

Table S.10 The table below summarizes either stabilization energies (E_{stab}) or total energies (E_{tot}) of thymine. The energies are given in kcal/mol.

**Thymine**	**D-H…A**	$\mathbf{E_{stab}}$ (14)	$\mathbf{E_{stab}}$ (13)	$\mathbf{E_{stab}}$ (8)	$\mathbf{E_{stab}}$ (10)	$\mathbf{E_{tot,\ est}}$ (11)
TT_mWW_(23)(34)	N3-H3…O2	-12,5	-9,9	-6,2	-9,1	-12,5
	N3-H3…O4					
TT_tWW_(23)(32)	N3-H3…O2	-11.8	-9.9	-6.5	-9.1	-12.4
TT_tWW_(34)(43)	N3-H3…O4	-13.1	-9.9	-6.2	-9.0	-12.5
TT_mWH_(34)(45)	N3-H3…O4	-5.5				
	C5-H5…O4					
TT_mWS_(12)(23)	N1-H1…O2	-15.0				
	N3-H3…O2					
TT_tSS_(12)(21)	N1-H1…O2	-18.9				

Table S.11 The table below summarizes either stabilization energies (E_{stab}) or total energies (E_{tot}) of uracil. The energies are given in kcal/mol.

**Uracil**	**D-H…A**	$\mathbf{E_{int}}$ (14)	$\mathbf{E_{stab}}$ (9)	$\mathbf{E_{stab}}$ (15)
UU_mWW_(23)(34)	N3-H3…O2	-12.2	-11.3	-10.6
	N3-H3…O4			
UU_tWW_(23)(32)	N3-H3…O2	-11.5		-10.6
UU_tWW_(34)(43)	N3-H3…O4	-13.4		-10.4
UU_mWH_(14)(25)	N1-H1…O4	-12.2		
	C5-H5…O2			
UU_mWH_(34)(45)	N3-H3…O4	-8.3		
	C5-H5…O4			
UU_tHH_(45)(54)	C5-H5…O4	-6.2		
UU_tSS_(12)(21)	N1-H1…O2	-16.4		-15.9

Table S.12 The table below summarizes either stabilization energies (E_{stab}) or total energies (E_{tot}) of cytosine. The energies are given in kcal/mol.

**Cytosine**		**E**$_{stab}$ (12)	**E**$_{stab}$ (13)	**E**$_{stab}$ (8)	**E**$_{stab}$ (10)	**E**$_{tot, est}$ (11)	**E**$_{tot}$ (16)
CC_tWW_(23)(32)	N3-H3…N2						
CC_tWW_(24)(33)(42)	N4-H4…O2	-11.8		-1.2			-42.45
	N3-H3…N3						
CC_tWW_(34)(43)	N4-H4…N3	-20.1	-17.5	-9.2	-17.3	-20.0	
CC_mWH_(24)(35)	N4-H4…O2	-9.9					
	C5-H5…N3						
CC_tWH_(25)(34)	C5-H5…O2	-10.1					
	N4-H4…N3						
CC_mWS_(26)(31)(42)	C6-H6…O2	-22.1					
	N1-H1…N3						
	N4-H4…O2						
CC_tSS_(12)(21)	N1-H1…O2	-22.8	0.0	-11.1			

Adenine

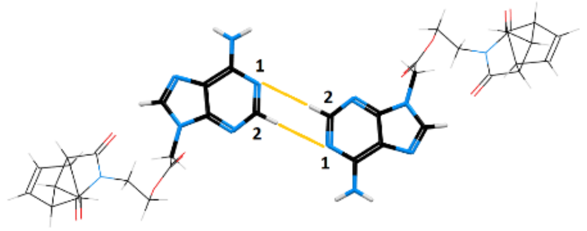<br>a) AA_mWW_(12)(21)<br>ACUHUB	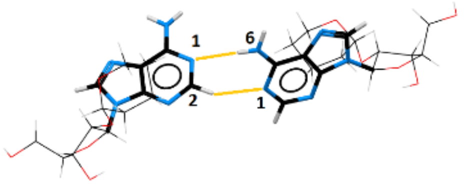<br>b) AA_fWW_(16)(21)<br>THOPAD10	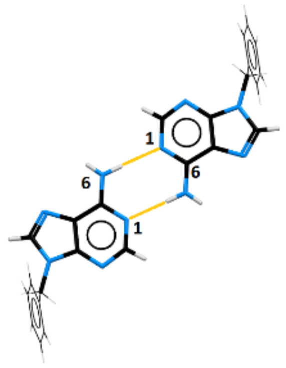<br>c) AA_mWW_(16)(61)<br>EWOCUP01	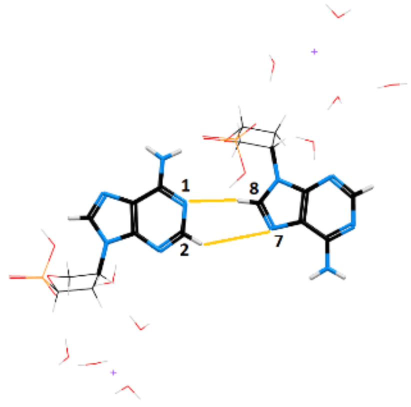<br>d) AA_fWH_(18)(27)<br>LOQROY
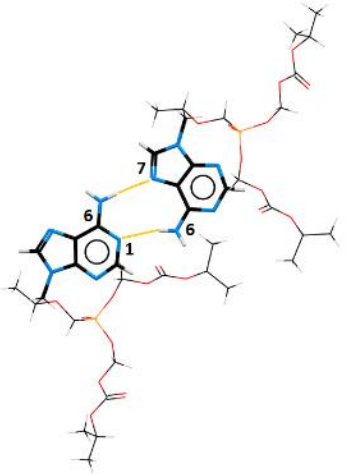<br>e) AA_fWH_(16)(67)<br>TEDTUU	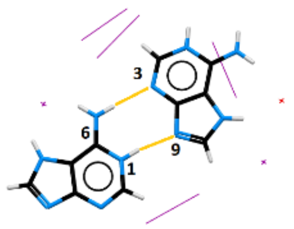<br>f) AA_fWS_(19)(63)<br>RELKUR	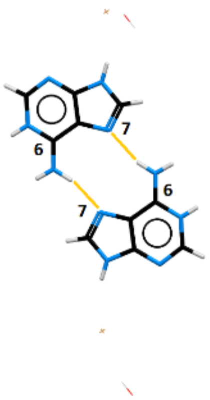<br>g) AA_mHH_(67)(76)<br>ADENBH	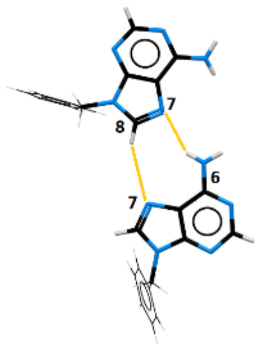<br>h) AA_fHH_(67)(78)<br>EWOCUP01

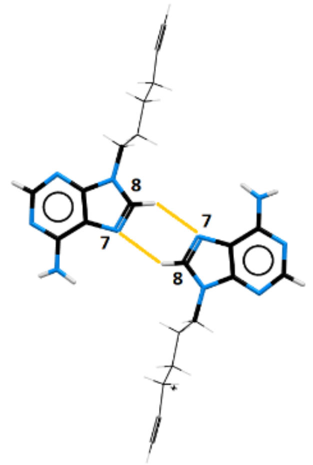

i) AA_mHH_(78)(87)
AWAYON

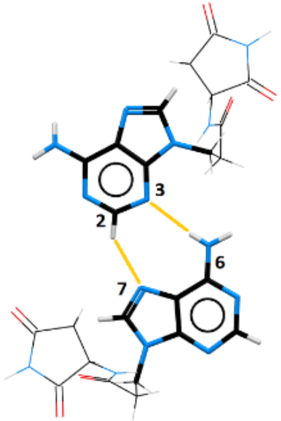

j) AA_fHS_(63)(72)
CUBRUM

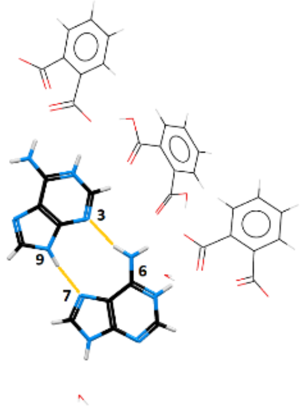

k) AA_mHS_(63)(79)
LICGIO

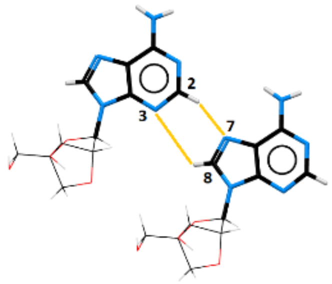

l) AA_mHS_(72)(83)
JACVAL

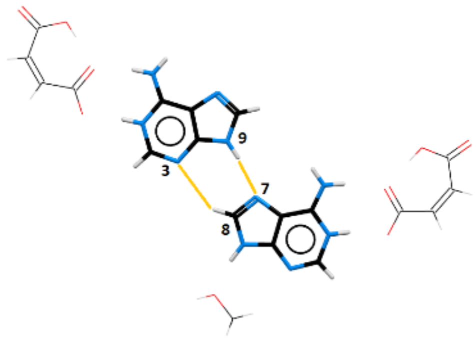

m) AA_fHS_(79)(83)
SEQTUF

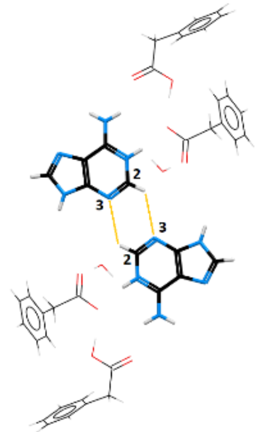

n) AA_mSS_(23)(32)
RIGMEA

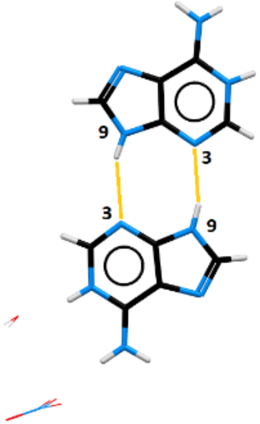

o) AA_mSS_(39)(93)
ADENOH10

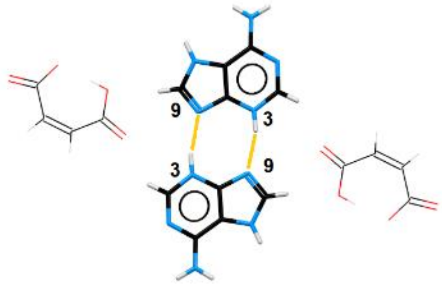

p) AA_mSS_(39)(93)
SEQVER, (alternative protonation)

Figure S.8 Examples of adenine structures containing particular base pair with their respective CSD refcodes.

Guanine

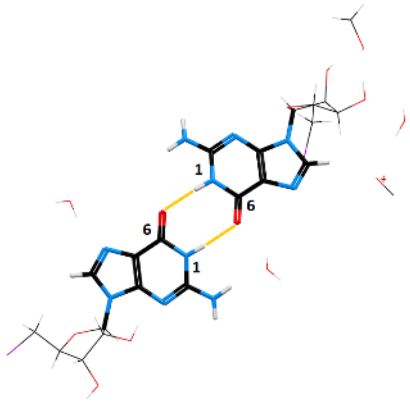<br>a) GG_mWW_(16)(61)<br>OXIJOW	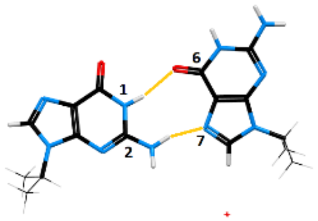<br>b) GG_mWH_(16)(27)<br>ZEVPIB	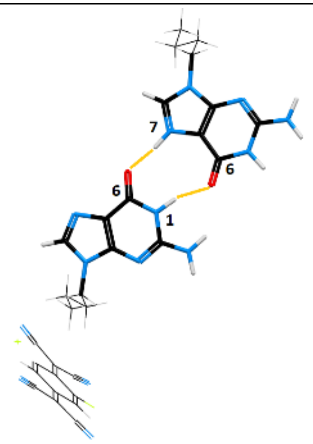<br>c) GG_fWH_(16)(67)<br>ASUVIU	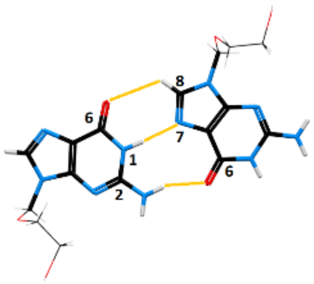<br>d) GG_fWH_(26)(17)(68)<br>MECWIC
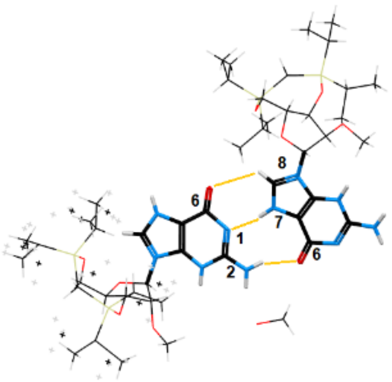<br>e) GG_fWH_(26)(17)(68)<br>REPGAV, (alternative protonation)	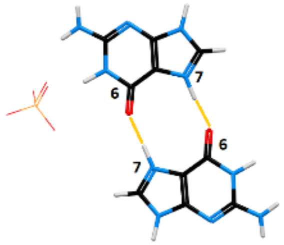<br>f) GG_mHH_(67)(76)<br>DUKKOJ	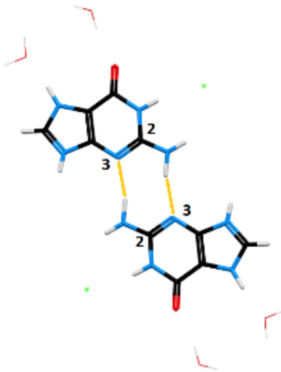<br>g) GG_mSS_(23)(32)<br>GUANCD	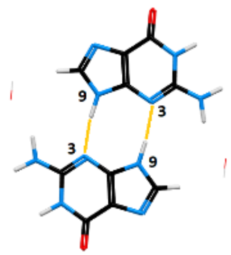<br>h) GG_mSS_(39)(93)<br>GUANMH10

Figure S.9 Examples of guanine structures containing particular base pair with their respective CSD refcodes.

Hypoxanthine

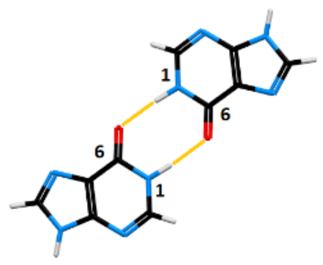

a) HxHx_mWW_(16)(61)
GEBTUC

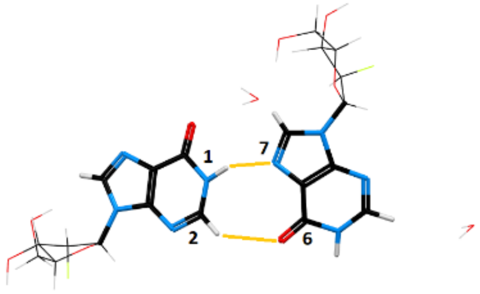

b) HxHx_fWH_(17)(26)
DOFLIN

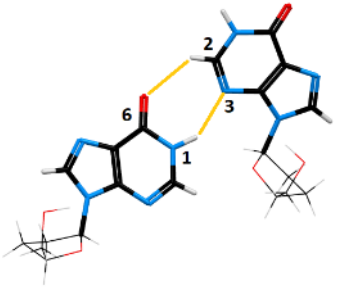

c) HxHx_fWS_(13)(62)
CUYDOQ

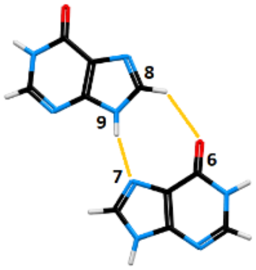

d) HxHx_fHS_(68)(79)
GEBTUC

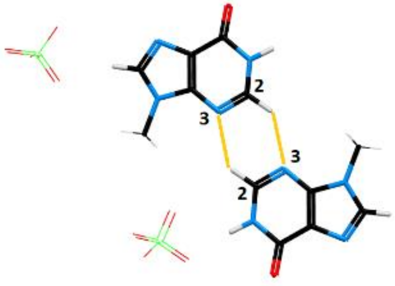

e) HxHx_mSS_(23)(32)
PAHWAX

Figure S.10 Examples of hypoxanthine structures containing particular base pair with their respective CSD refcodes.

Thymine

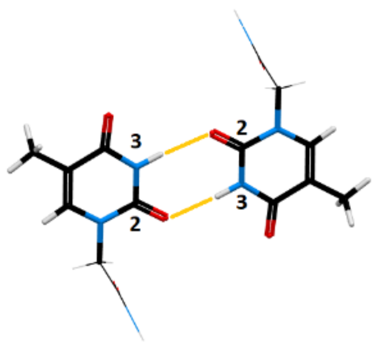

a) TT_mWW_(23)(32)
GEHVOF

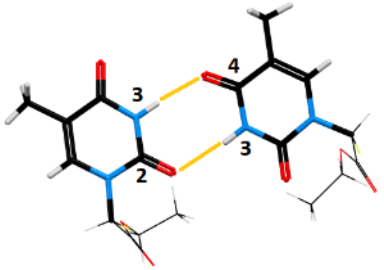

b) TT_fWW_(23)(34)
UTEWIB

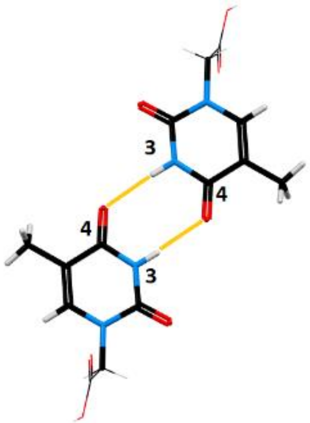

c) TT_mWW_(34)(43)
ABEPIH

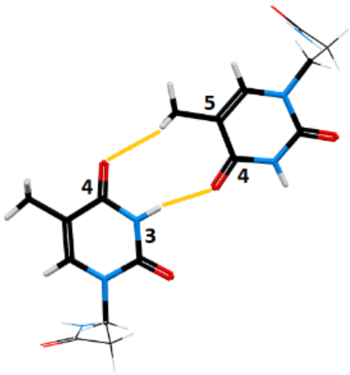

d) TT_fWH_(34)(45)
GAKSEQ01

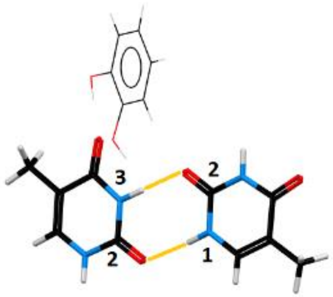

e) TT_fWS_(21)(32)
OGIYEK

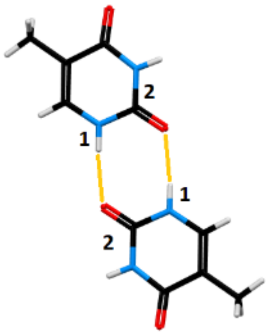

f) TT_mSS_(12)(21)
THYMIN03

Figure S.11 Examples of thymine structures containing particular base pair with their respective CSD refcodes.

Uracil

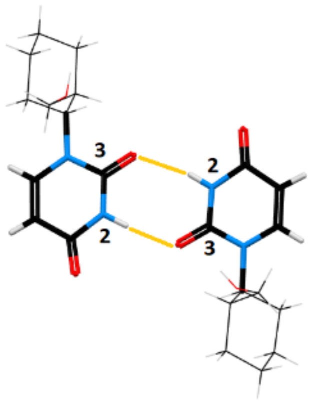<br>a) UU_mWW_(23)(32)<br>BINFEK	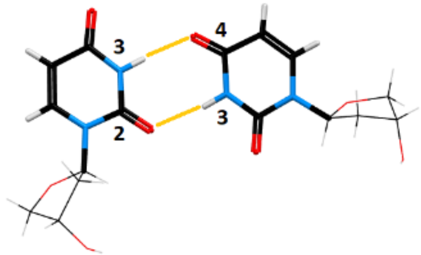<br>b) UU_fWW_(23)(34)<br>QIXYUR	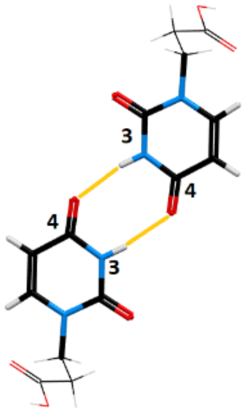<br>c) UU_mWW_(34)(43)<br>BIYRIK	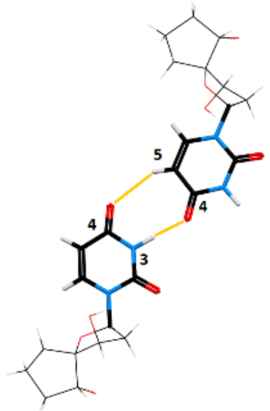<br>d) UU_fWH_(34)(45)<br>QAHZAB
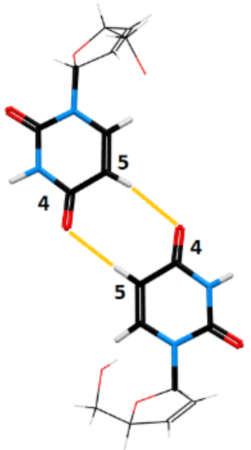<br>e) UU_mHH_(45)(54)<br>WAXLEM	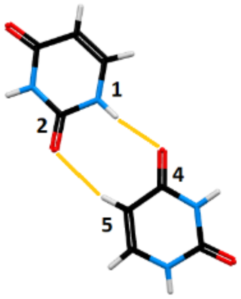<br>f) UU_mHS_(41)(52)<br>URACIL	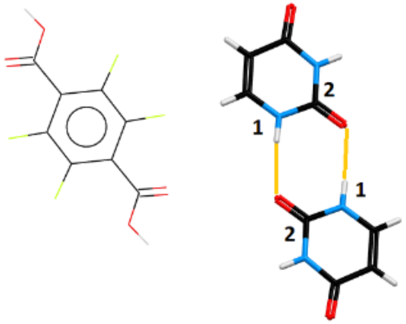<br>g) UU_mSS_(12)(21)<br>INONAC	

Figure S.12 Examples of uracil structures containing particular base pair with their respective CSD refcodes.

Cytosine

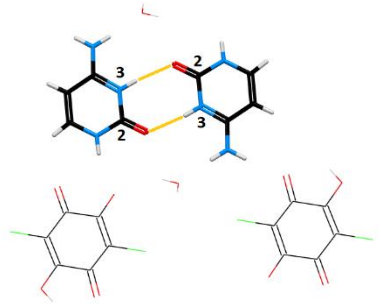<br>a) CC_mWW_(23)(32)<br>MCYTOS	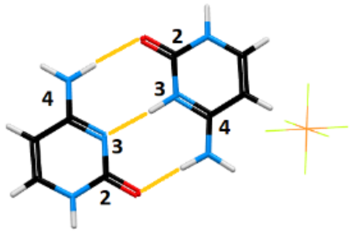<br>b) CC_mWW_(24)(33)(42)<br>YUMGOD	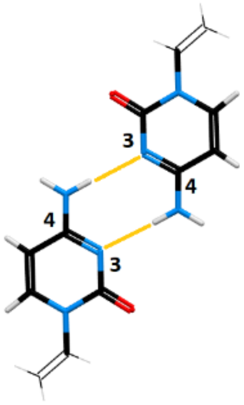<br>c) CC_mWW_(34)(43)<br>BOMTAY	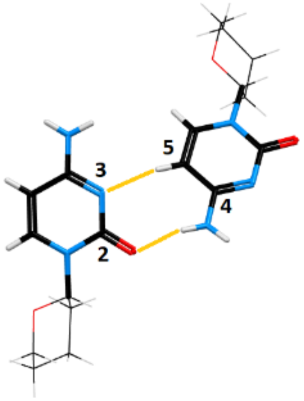<br>d) CC_fWH_(24)(35)<br>XOFZAV
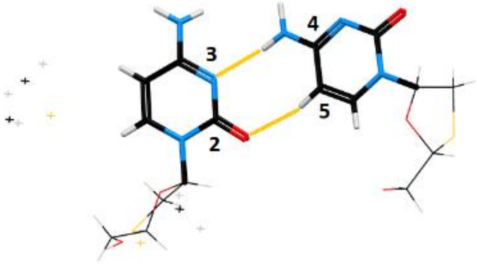<br>e) CC_mWH_(25)(34)<br>RUKGUZ	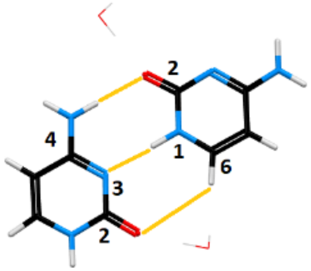<br>f) CC_fWS_(26)(31)(42)<br>CYTOSM14	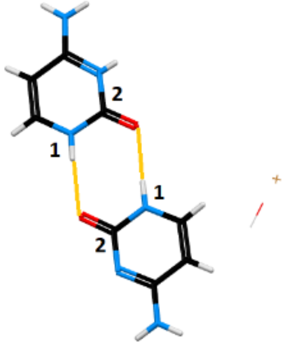<br>g) CC_mSS_(12)(21)<br>ZEYJOE	

Figure S.13 Examples of cytosine structures containing particular base pair with their respective CSD refcodes.

References

1. Wilcox,J.L., Ahluwalia,A.K., Bevilacqua,P.C., Biology,S. and Fedor,M. (2011) Charged Nucleobases and Their Potential for RNA Catalysis. *Acc. Chem. Res.*, **44**, 1270–1279.

2. Thaplyal,P. and Bevilacqua,P.C. (2014) Experimental approaches for measuring pKa's in RNA and DNA 1st ed. Elsevier Inc.

3. Krishnamurthy,R. (2013) Correction to Role of p K a of Nucleobases in the Origins of Chemical Evolution . *Acc. Chem. Res.*, **46**, 1670–1670.

4. Schnabl,J. and Sigel,R.K. (2010) Controlling ribozyme activity by metal ions. *Curr. Opin. Chem. Biol.*, **14**, 269–275.

5. Kelly,R.E.A., Lee,Y.J. and Kantorovich,L.N. (2005) Homopairing possibilities of the DNA base adenine. *J. Phys. Chem. B*, **109**, 11933–11939.

6. Chen,Q., Frankel,D.J. and Richardson,N. V. (2002) Self-Assembly of Adenine on Cu(110) Surfaces. *Langmuir*, **18**, 3219–3225.

7. Sowerby,S.J., Edelwirth,M. and Heckl,W.M. (1998) Self-Assembly at the Prebiotic Solid−Liquid Interface: Structures of Self-Assembled Monolayers of Adenine and Guanine Bases Formed on Inorganic Surfaces. *J. Phys. Chem. B*, **102**, 5914–5922.

8. Furukawa,M., Tanaka,H. and Kawai,T. (2001) The role of dimer formation in the self-assemblies of DNA base molecules on Cu(111) surfaces: A scanning tunneling microscope study. *J. Chem. Phys.*, **115**, 3419–3423.

9. Šponer,J., Jurečka,P., Hobza,P., Šponer,J., Jurečka,P. and Hobza,P. (2004) Accurate interaction energies of hydrogen-bonded nucleic acid base pairs. *J. Am. Chem. Soc.*, **126**, 10142–10151.

10. Hobza,P. and Šponer,J. (1999) Structure, Energetics, and Dynamics of the Nucleic Acid Base Pairs: Nonempirical Ab Initio Calculations. *Chem. Rev.*, **99**, 3247–3276.

11. Šponer,J., Leszczyński,J. and Hobza,P. (2001) Hydrogen bonding, stacking and cation binding of DNA bases. *J. Mol. Struct.*, **573**, 43–53.

12. Kelly,R.E.A., Lee,Y.J. and Kantorovich,L.N. (2005) Homopairing possibilities of the DNA bases cytosine and guanine: An ab initio DFT study. *J. Phys. Chem. B*, **109**, 22045–22052.

13. Šponer,J., Leszczynski,J. and Hobza,P. (1996) Structures and Energies of Hydrogen-Bonded DNA Base Pairs. A Nonempirical Study with Inclusion of Electron Correlation. *J.*

Phys. Chem., **100**, 1965–1974.

14. Kelly,R.E.A. and Kantorovich,L.N. (2006) Homopairing Possibilities of the DNA Base Thymine and the RNA Base Uracil: An ab Initio Density Functional Theory Study. *J. Phys. Chem. B*, **110**, 2249–2255.

15. Kratochvıl,M., Engkvist,O., Jungwirth,P., Hobza,P. and Heyro,J. V. (1998) Uracil Dimer : Potential Energy and Free Energy Surfaces . Ab Initio beyond Hartree - Fock and Empirical Potential Studies. *J. Phys. Chem. A*, **102**, 6921–6926.

16. Chawla,M., Sharma,P., Halder,S., Bhattacharyya,D. and Mitra,A. (2011) Protonation of base pairs in RNA : Context analysis and Quantum Chemical investigations of their geometries and stabilities. *J. Phys. Chem. B*, **115**, 1469–1484.

Figures

Figure S.1 Naming scheme for the edges of investigated nucleobases. The particular edges are color coded in the same way as in the rest of the publication – Watson-Crick edge is blue, Hoogsteen edge is yellow, and Sugar edge - red.

Figure S.2 Atom naming scheme for the nucleobases with the protonation patterns observed among the investigated structures containing base pairs.

Figure S.3 Examples of flipped and moved orientation of the base pairs.

Figure S.4 Example of a hypothetical hetero base pair - AG_mHW_(61)(76).

Figure S.5 Frequencies of occurrence of base pairs for adenine with partition into charged and neutral state. The structures without the hydrogen positions determined were not included.

Figure S.6 Frequencies of occurrence of base pairs for guanine with partition into charged and neutral state. The structures without the hydrogen positions determined were not included.

Figure S.7 Frequencies of occurrence of base pairs for cytosine with partition into charged and neutral state. The structures without the hydrogen positions determined were not included.

Figure S.8 Examples of adenine structures containing particular base pair with their respective CSD refcodes.

Figure S.9 Examples of guanine structures containing particular base pair with their respective CSD refcodes.

Figure S.10 Examples of hypoxanthine structures containing particular base pair with their respective CSD refcodes.

Figure S.11 Examples of thymine structures containing particular base pair with their respective CSD refcodes.

Figure S.12 Examples of uracil structures containing particular base pair with their respective CSD refcodes.

Figure S.13 Examples of cytosine structures containing particular base pair with their respective CSD refcodes.

Tables

Table S.1 Table presents the average lengths of hydrogen bonds present in adenine base pairs illustrated in Figure 4 in the main text. N_{all} column tells how many separate entries there were in CSD for particular base pair. N_H is the number of structures with hydrogen positions determined. N_{uniq} is the number of unique structures present in CSD, as they are represented in pie charts in Figure 3.a in the main text. The D-H bond lengths were adjusted to their neutron lengths to compensate for the underestimate of the X-ray methods.

Table S.2 Table presents the average lengths of hydrogen bonds present in guanine base pairs illustrated in Figure 5 in the main text. N_{all} column tells how many separate entries there were in CSD for particular base pair. N_H is the number of structures with hydrogen positions determined. N_{uniq} is the number of unique structures present in CSD, as they are represented in pie charts in Figure 3.e in the main text. The D-H bond lengths were adjusted to their neutron lengths to compensate for the underestimate of the X-ray methods.

Table S.3 Table presents the average lengths of hydrogen bonds present in hypoxanthine base pairs illustrated in Figure 6 in the main text. N_{all} column tells how many separate entries there were in CSD for particular base pair. N_H is the number of structures with hydrogen positions determined. N_{uniq} is the number of unique structures present in CSD, as they are represented in pie charts in Figure 3.i in the main text. The D-H bond lengths were adjusted to their neutron lengths to compensate for the underestimate of the X-ray methods.

Table S.4 Table presents the average lengths of hydrogen bonds present in thymine base pairs illustrated in Figure 7 in the main text. N_{all} column tells how many separate entries there were in CSD for particular base pair. N_H is the number of structures with hydrogen positions determined. N_{uniq} is the number of unique structures present in CSD, as they are represented in pie charts in Figure 4.a in the main. The D-H bond lengths were adjusted to their neutron lengths to compensate for the underestimate of the X-ray methods.

Table S.5 Table presents the average lengths of hydrogen bonds present in uracil base pairs illustrated in Figure 8 in the main text. N_{all} column tells how many separate entries there were in CSD for particular base pair. N_H is the number of structures with hydrogen positions determined. N_{uniq} is the number of unique structures present in CSD, as they are represented in pie charts in Figure 4.d in the main text. The D-H bond lengths were adjusted to their neutron lengths to compensate for the underestimate of the X-ray methods.

Table S.6 Table presents the average lengths of hydrogen bonds present in cytosine base pairs illustrated in Figure 9 in the main text. N_{all} column tells how many separate entries there were in CSD for particular base pair. N_H is the number of structures with hydrogen positions

determined. N_{uniq} is the number of unique structures present in CSD, as they are represented in pie charts in Figure 4.h in the main text. The D-H bond lengths were adjusted to their neutron lengths to compensate for the underestimate of the X-ray methods.

Table S.7 Summary of pK_a values for nitrogen atoms in nucleobases.

Table S.8 The table below summarizes either stabilization energies (E_{stab}) or total energies (E_{tot}) of adenine. The energies are given in kcal/mol.

Table S.9 The table below summarizes either stabilization energies (E_{stab}) or total energies (E_{tot}) of guanine. The energies are given in kcal/mol.

Table S.10 The table below summarizes either stabilization energies (E_{stab}) or total energies (E_{tot}) of thymine. The energies are given in kcal/mol.

Table S.11 The table below summarizes either stabilization energies (E_{stab}) or total energies (E_{tot}) of uracil. The energies are given in kcal/mol.

Table S.12 The table below summarizes either stabilization energies (E_{stab}) or total energies (E_{tot}) of cytosine. The energies are given in kcal/mol.
