# Supplementary material for: Frequency and hydrogen bonding of nucleobase homopairs in small molecule crystals
Source: Nucleic Acids Res. 2020 Jul 29;48(15):8302–19. doi: 10.1093/nar/gkaa629 (PMC7470937; doi:10.1093/nar/gkaa629)
Supplement: gkaa629_Supplemental_Files [file gkaa629_supplemental_files.zip › Supplementary2_20200710.pdf]

Frequency and hydrogen bonding of nucleobase homopairs in small molecule crystals – supplementary data

Małgorzata Katarzyna Cabaj[1] and Paulina Maria Dominiak[1,]*

[1] Biological and Chemical Research Center, Department of Chemistry, University of Warsaw, ul. Żwirki i Wigury 101, 02-089 Warszawa, Poland

[*] To whom correspondence should be addressed. Tel: +48 22 55 26 714; Fax: not applicable; Email: pdomin@chem.uw.edu.pl

ORCID

Małgorzata Katarzyna Cabaj: 0000-0001-9184-4513

Paulina Maria Dominiak: 0000-0002-1466-1243

Keywords

nucleobase pairs, hydrogen bonds, small molecule crystals, nucleobases protonation

Key points

1. Frequency of occurrence of nucleobase pairs in small molecule crystal structures
2. Hydrogen bond geometry of nucleobase pairs in small molecule crystal structures
3. Protonation of nucleobase pairs in small molecule crystal structures

Here we wish to present an atlas of base pairs found in small molecule crystal structures with all the data facilitating easy analysis of the base pair geometry and comparison with analogous base pair found in RNA crystal structures.

Most of the content of each base pair “card” is self-explanatory.

The Leontis-Westhof name is the name of the base pair in the convention set by Leontis and Westhof in their publication (Leontis, N.B. and Westhof, E. (2001) Geometric nomenclature and classification of RNA base pairs. RNA, 7, 499–512). This convention is suited for RNA base pairs, and in many cases does not fit base pairs found in small molecule crystal structures. In the case of base pairs that are unique only to small molecule crystal structures there is a question mark (?) next to the Leontis-Westhof name indicating that the name may not fit the pair too well.

In the “Protonation” part the numbers 1 3 7 9 correspond to the nitrogen atoms present in purines, and 1 3 – nitrogen atoms in pyrimidine rings. If the number is free, then this particular nitrogen is not protonated, if the letter “H” is present, then this spot is protonated. If there is “R”, then it indicates the derivative of a particular nucleobase. Question marks are representing structures without proton positions determined.

In the hydrogen bond lengths part the N_{all} column tells how many separate entries there were in CSD for particular base pair. N_H is the number of structures with hydrogen positions determined. The N_{uniq} is the number of unique structures present in CSD.

ADENINE

**AA_mWW_(12)(21)**	**Leontis-Westhof name**	tWW (?)

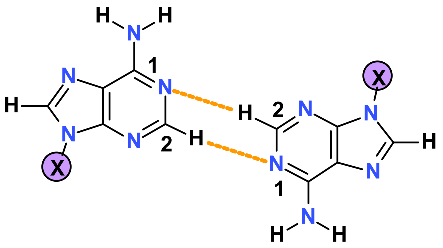

unique in CSD	16
in RNA Basepair Catalog	-

**Division**		**Protonation patterns**	
substituted neutral	15	1 3 7 9R	13
		R?	2
substituted charged	0		
free neutral	1	1 3 7 9H	1
free charged	0		

**D-H…A**	**D…A [Å]**	**D-H [Å]**	**H…A [Å]**	**D-H…A [º]**	**C1’…C1’ [Å]**	**N_{all}**	**N_H**	**N_{uniq}**
C2-H2…N1	3.9 (2)	1.089	3.0 (2)	145 (16)	11.9 (4)	18	16	16

**AA_fWW_(16)(21)**	**Leontis-Westhof name**	cWW

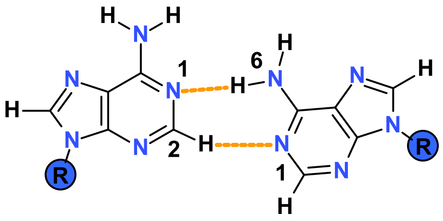

unique in CSD	1
in RNA Basepair Catalog	34

**Division**		**Protonation patterns**	
substituted neutral	1	1 3 7 9R	1
substituted charged	0		
free neutral	0		
free charged	0		

**D-H…A**	**D…A [Å]**	**D-H [Å]**	**H…A [Å]**	**D-H…A [º]**	**C1’…C1’ [Å]**	**N_{all}**	**N_H**	**N_{uniq}**
N6-H6…N1	3.214	1.015	2.201	175	12.000	1	1	1
C2-H2…N1	3.251	1.089	2.316	143				

AA_mWW_(16)(61)	Leontis-Westhof name			tWW
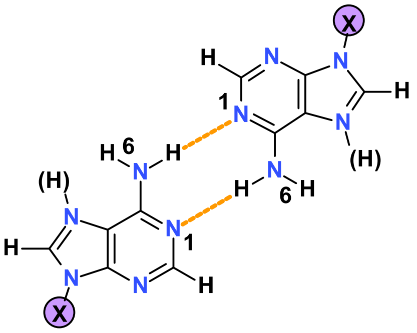	unique in CSD			43
	in RNA Basepair Catalog			69
	**Division**		**Protonation patterns**	
	substituted neutral	36	1 3 7 9R	32
			R?	4
	substituted charged	0		
	free neutral	7	1 3 7 9H	4
			1 3 7H 9	3
	free charged	0		

D-H…A	D…A [Å]	D-H [Å]	H…A [Å]	D-H…A [º]	C1'…C1' [Å]	N_{all}	N_H	N_{uniq}
N6-H6…N1	3.06 (16)	1.015	2.1 (3)	166 (17)	13.75(12)	45	41	43

AA_fWH_(18)(27)	Leontis-Westhof name			tWH (?)
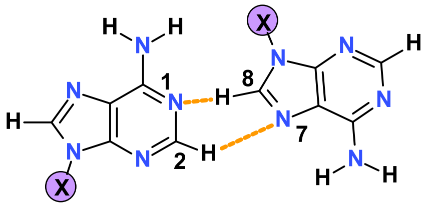	unique in CSD			3
	in RNA Basepair Catalog			-
	**Division**		**Protonation patterns**	
	substituted neutral	2	1 3 7 9R	2
	substituted charged	0		
	free neutral	1	1 3 7 9H	1
	free charged	0		

D-H…A	D…A [Å]	D-H [Å]	H…A [Å]	D-H…A [º]	C1'…C1' [Å]	N_{all}	N_H	N_{uniq}
C8-H8…N1	3.61 (15)	1.089	2.7 (2)	142 (17)	9.97 (14)	4	4	3
C2-H2…N7	3.83 (17)	1.089	3.03(13)	130 (5)				

AA_fWH_(16)(67)

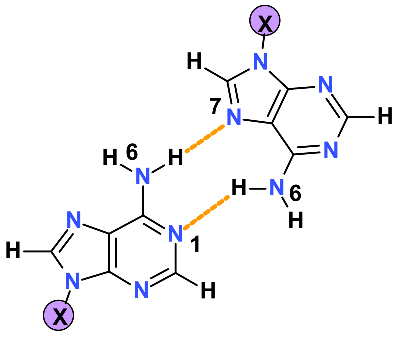

Leontis-Westhof name	tWH
unique in CSD	106
in RNA Basepair Catalog	94

Division		Protonation patterns	
substituted neutral	105	1 3 7 9R	98
		R?	7
substituted charged	0		
free neutral	1	1 3 7 9H	1
free charged	0		

D-H…A	D…A [Å]	D-H [Å]	H…A [Å]	D-H…A [º]	C1'…C1' [Å]	N_{all}	N_{H}	N_{uniq}
N6-H6…N1	2.97 (6)	1.015	2.01 (6)	158 (7)	12.33 (9)	152	144	106
N6-H6…N7	3.04 (6)	1.015	2.1 (1)	170 (7)				

AA_fWS_(19)(63)

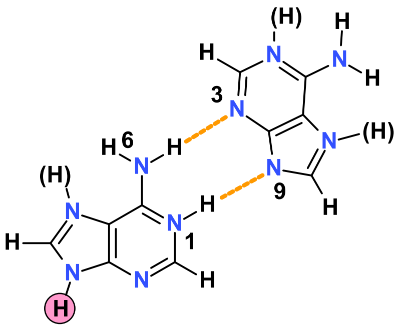

Leontis-Westhof name	cWS (?)
unique in CSD	3
in RNA Basepair Catalog	-

Division		Protonation patterns	
substituted neutral	0		
substituted charged	0		
free neutral	0		
free charged	3	1H 3 7H 9H	2
		1 3 7H 9<br>1H 3 7 9H	1

D-H…A	D…A [Å]	D-H [Å]	H…A [Å]	D-H…A [º]	C1'…C1' [Å]	N_{all}	N_{H}	N_{uniq}
N1-H1…N9	2.92 (8)	1.015	1.77 (2)	171 (4)	-	4	4	3
N6-H6…N3	2.82 (8)	1.015	1.95 (4)	174 (2)				

AA_mHH_(67)(76)	Leontis-Westhof name			tHH
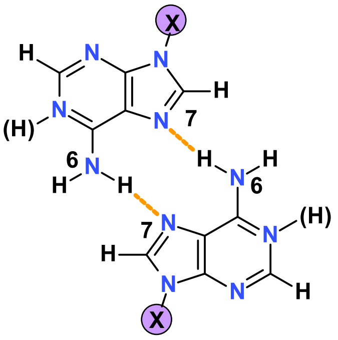	unique in CSD			86
	in RNA Basepair Catalog			320
	**Division**		**Protonation patterns**	
	substituted neutral	39	1 3 7 9R	27
			R?	12
	substituted charged	23	1H 3 7 9R	23
	free neutral	4	1 3 7 9H	2
			No R?	2
	free charged	20	1H 3 7 9H	19
			1 3 7 9H<br>1H 3 7 9H	1

**D-H…A**	**D…A [Å]**	**D-H [Å]**	**H…A [Å]**	**D-H…A [º]**	**C1'…C1' [Å]**	**N_{all}**	**N_{H}**	**N_{uniq}**
N6-H6…N7	3.0 (1)	1.015	2.0 (2)	158 (9)	11.1 (3)	97	78	86

AA_mHH_(67)(78)	Leontis-Westhof name			cHH (?)
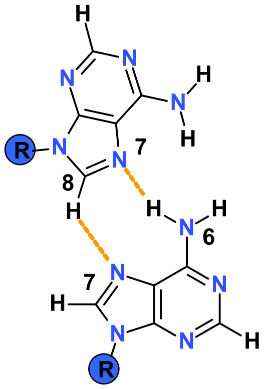	unique in CSD			2
	in RNA Basepair Catalog			-
	**Division**		**Protonation patterns**	
	substituted neutral	2	1 3 7 9R	2
	substituted charged	0		
	free neutral	0		
	free charged	0		

**D-H…A**	**D…A [Å]**	**D-H [Å]**	**H…A [Å]**	**D-H…A [º]**	**C1'…C1' [Å]**	**N_{all}**	**N_{H}**	**N_{uniq}**
N6-H6…N7	3.194 (8)	1.015	2.34 (2)	142 (4)	8.5 (1)	2	2	2
C8-H8…N7	3.81 (7)	1.089	2.89 (7)	144 (2)				

**AA_mHH_(78)(87)**	**Leontis-Westhof name**	tHH (?)
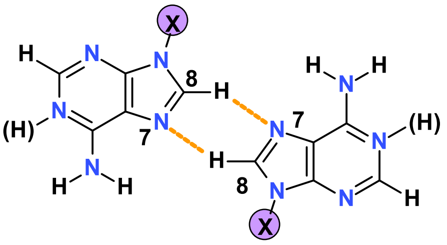	unique in CSD	15
	in RNA Basepair Catalog	-

**Division**		**Protonation patterns**	
substituted neutral	12	1 3 7 9R	11
		R?	1
substituted charged	0		
free neutral	2	1 3 7 9H	2
free charged	1	1H 3 7 9H	1

**D-H…A**	**D…A [Å]**	**D-H [Å]**	**H…A [Å]**	**D-H…A [º]**	**C1'…C1' [Å]**	**N_{all}**	**N_{H}**	**N_{uniq}**
C8-H8…N7	3.7 (2)	1.089	2.90(18)	127 (5)	8.0 (3)	23	22	15

**AA_fHS_(63)(72)**	**Leontis-Westhof name**	tHS
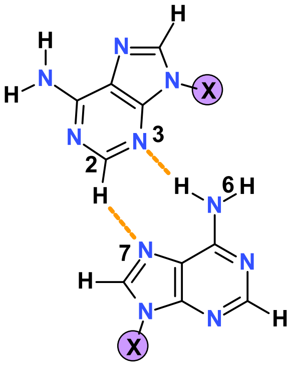	unique in CSD	2
	in RNA Basepair Catalog	92

**Division**		**Protonation patterns**	
substituted neutral	1	1 3 7 9R	1
substituted charged	0		
free neutral	1	1 3 7 9H	1
free charged	0		

**D-H…A**	**D…A [Å]**	**D-H [Å]**	**H…A [Å]**	**D-H…A [º]**	**C1'…C1' [Å]**	**N_{all}**	**N_{H}**	**N_{uniq}**
N6-H6…N3	3.02 (5)	1.015	2.02 (5)	168 (0)	9.447 (5)	2	2	2
C2-H2…N7	3.26 (3)	1.015	2.30 (1)	145 (3)				

AA_mHS_(63)(79)	Leontis-Westhof name	cHS (?)
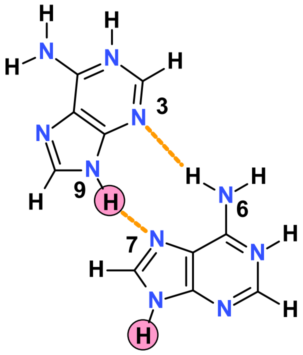	unique in CSD	1
	in RNA Basepair Catalog	-

Division		Protonation patterns	
substituted neutral	0		
substituted charged	0		
free neutral	0		
free charged	1	1H 3 7 9H	1

D-H…A	D…A [Å]	D-H [Å]	H…A [Å]	D-H…A [º]	C1'…C1' [Å]	N_{all}	N_H	N_{uniq}
N6-H6…N3	2.935	1.015	1.937	167	-	1	1	1
N9-H9…N7	2.888	1.015	1.882	171				

AA_mHS_(72)(83)	Leontis-Westhof name	cHS (?)
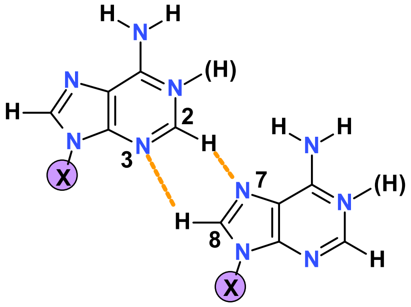	unique in CSD	9
	in RNA Basepair Catalog	-

Division		Protonation patterns	
substituted neutral	5	1 3 7 9R	5
substituted charged	1	1H 3 7 9R	1
free neutral	0		
free charged	3	1H 3 7 9H	3

D-H…A	D…A [Å]	D-H [Å]	H…A [Å]	D-H…A [º]	C1'…C1' [Å]	N_{all}	N_H	N_{uniq}
C2-H2…N7	3.5 (2)	1.089	2.7 (3)	130 (13)	7.1 (3)	11	11	9
C8-H8…N3	3.9 (2)	1.089	3.3 (1)	118 (8)				

AA_fHS_(79)(83)	Leontis-Westhof name		cHS (?)
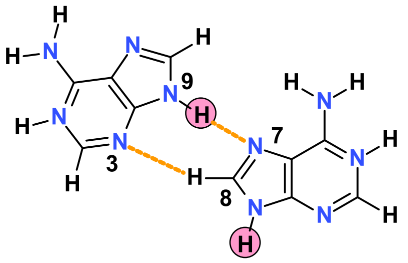	unique in CSD		1
	in RNA Basepair Catalog		-
	**Division**		**Protonation patterns**
	substituted neutral	0	
	substituted charged	0	
	free neutral	0	
	free charged	1	1H 3 7 9H 1

D-H…A	D…A [Å]	D-H [Å]	H…A [Å]	D-H…A [º]	C1'…C1' [Å]	N_{all}	N_H	N_{uniq}
N9-H9…N7	2.921	1.015	1.914	171	-	1	1	1
C8-H8…N3	3.608	1.089	2.677	143				

AA_mSS_(23)(32)	Leontis-Westhof name		tSS	
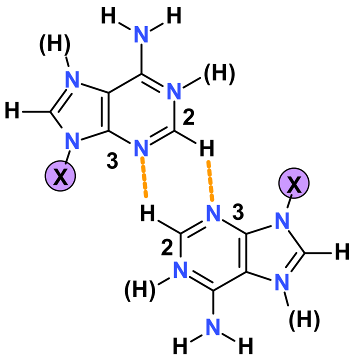	unique in CSD			15
	in RNA Basepair Catalog			-
	**Division**		**Protonation patterns**	
	substituted neutral	8	1 3 7 9R	5
			R?	3
	substituted charged	2	1H 3 7 9R	2
	free neutral	1	No R?	1
	free charged	4	1H 3 7 9H	3
			1H 3 7H 9H	1

D-H…A	D…A [Å]	D-H [Å]	H…A [Å]	D-H…A [º]	C1'…C1' [Å]	N_{all}	N_H	N_{uniq}
C2-H2…N3	3.5 (3)	1.089	2.8 (3)	126 (11)	8.9 (8)	22	16	15

**AA_mSS_(39)(93)**	**Leontis-Westhof name**			tSS (?)
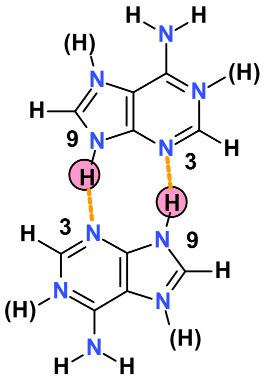	unique in CSD			21
	in RNA Basepair Catalog			-
	**Division**		**Protonation patterns**	
	substituted neutral	0		
	substituted charged	0		
	free neutral	7	1 3 7 9H	6
			No R?	1
	free charged	14	1H 3 7 9H	10
			1H 3 7H 9H	3
			1 3 7 9H<br>1H 3 7 9H	1

**D-H…A**	**D…A [Å]**	**D-H [Å]**	**H…A [Å]**	**D-H…A [º]**	**C1'…C1' [Å]**	**N_{all}**	**N_H**	**N_{uniq}**
N9-H9…N3	2.90 (7)	1.015	1.92 (8)	162 (5)	-	23	22	21

**AA_mSS_(39)(93)**<br>**(alternative protonation)**	**Leontis-Westhof name**			tSS (?)
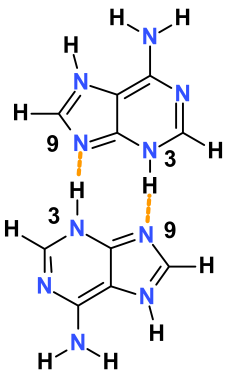	unique in CSD			1
	in RNA Basepair Catalog			-
	**Division**		**Protonation patterns**	
	substituted neutral	0		
	substituted charged	0		
	free neutral	0		0
	free charged	1	1 3H 7H 9	1

**D-H…A**	**D…A [Å]**	**D-H [Å]**	**H…A [Å]**	**D-H…A [º]**	**C1'…C1' [Å]**	**N_{all}**	**N_H**	**N_{uniq}**
N3-H3…N9	2.808	1.015	1.826	162	-	1	1	1

GUANINE

**GG_mWW_(16)(61)**	**Leontis-Westhof name**	tWW
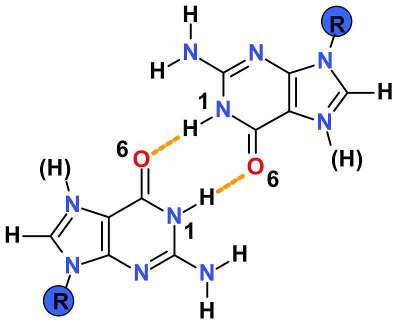	unique in CSD	2
	in RNA Basepair Catalog	10

**Division**		**Protonation patterns**	
substituted neutral	1	1H 3 7 9R	1
substituted charged	1	1H 3 7H 9R	1
free neutral	0		
free charged	0		

**D-H…A**	**D…A [Å]**	**D-H [Å]**	**H…A [Å]**	**D-H…A [º]**	**C1'…C1' [Å]**	N_{all}	N_H	N_{uniq}
N1-H1…O6	2.79 (3)	1.015	1.80 (4)	164 (3)	13.39 (7)	2	2	2

**GG_mWH_(16)(27)**	**Leontis-Westhof name**	cWH
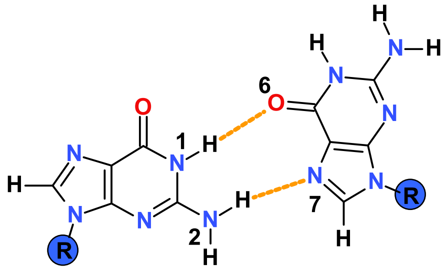	unique in CSD	11
	in RNA Basepair Catalog	123

**Division**		**Protonation patterns**	
substituted neutral	11	1H 3 7 9R	11
substituted charged	0		
free neutral	0		
free charged	0		

**D-H…A**	**D…A [Å]**	**D-H [Å]**	**H…A [Å]**	**D-H…A [º]**	**C1'…C1' [Å]**	N_{all}	N_H	N_{uniq}
N1-H1…O6	2.88 (4)	1.015	1.91 (4)	167 (5)	11.59 (9)	47	47	11
N2-H2…N7	2.91 (5)	1.015	1.92 (5)	161 (3)				

<table>
<tr><th colspan="2">GG_fWH_(16)(67)</th><th colspan="4">Leontis-Westhof name</th><th colspan="4">cWH (?)</th></tr>
<tr><td colspan="2" rowspan="4">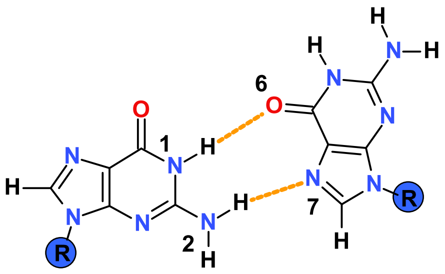</td><td colspan="8">unique in CSD 1
in RNA Basepair Catalog -</td></tr>
<tr><td colspan="4">Division</td><td colspan="4">Protonation patterns</td></tr>
<tr><td colspan="3">substituted neutral
substituted charged
free neutral
free charged</td><td>0
1
0
0</td><td colspan="3">
1H 3 7 9R
1H 3 7H 9R</td><td>
1</td></tr>
<tr></tr>
<tr><td>D-H…A</td><td>D…A [Å]</td><td>D-H [Å]</td><td>H…A [Å]</td><td>D-H…A [º]</td><td>C1’…C1’ [Å]</td><td>N_{all}</td><td>N_H</td><td>N_{uniq}</td><td></td></tr>
<tr><td>N1-H1…O6</td><td>2.799</td><td>1.015</td><td>1.804</td><td>166</td><td>12.050</td><td>1</td><td>1</td><td>1</td><td></td></tr>
<tr><td>N7-H7…O6</td><td>2.687</td><td>1.015</td><td>1.678</td><td>173</td><td></td><td></td><td></td><td></td><td></td></tr>
</table>

<table>
<tr><th colspan="2">GG_fWH_(26)(17)(68)</th><th colspan="4">Leontis-Westhof name</th><th colspan="4">tWH</th></tr>
<tr><td colspan="2" rowspan="3">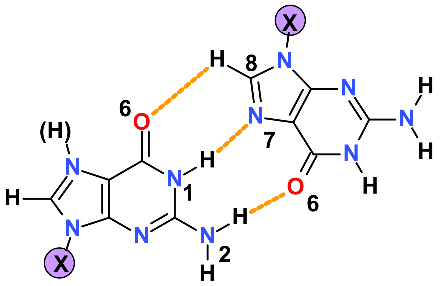</td><td colspan="8">unique in CSD 48
in RNA Basepair Catalog 48</td></tr>
<tr><td colspan="4">Division</td><td colspan="4">Protonation patterns</td></tr>
<tr><td colspan="3">substituted neutral

substituted charged
free neutral

free charged</td><td>45

1
2

0</td><td colspan="3">1H 3 7 9R
R?
1 3H 7H 9R
1H 3 7 9H
No R?</td><td>43
2
1
1
1</td></tr>
<tr><td>D-H…A</td><td>D…A [Å]</td><td>D-H [Å]</td><td>H…A [Å]</td><td>D-H…A [º]</td><td>C1’…C1’ [Å]</td><td>N_{all}</td><td>N_H</td><td>N_{uniq}</td><td></td></tr>
<tr><td>N2-H2…O6</td><td>2.90 (7)</td><td>1.015</td><td>1.9 (1)</td><td>155 (9)</td><td>11.2 (1)</td><td>77</td><td>74</td><td>48</td><td></td></tr>
<tr><td>N1-H1…N7</td><td>2.84 (4)</td><td>1.015</td><td>1.84 (4)</td><td>169 (6)</td><td></td><td></td><td></td><td></td><td></td></tr>
<tr><td>C8-H8…O6</td><td>3.4 (1)</td><td>1.089</td><td>2.8 (2)</td><td>118 (6)</td><td></td><td></td><td></td><td></td><td></td></tr>
</table>

GG_fWH_(26)(17)(68) (alternative protonation)	Leontis-Westhof name	tWH

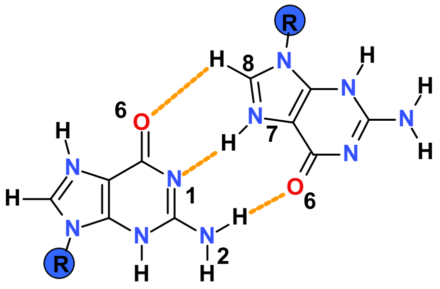

unique in CSD		1
in RNA Basepair Catalog		-

**Division**		**Protonation patterns**	
substituted neutral	0		
substituted charged	1	1H 3 7 9R<br>1H 3 7H 9R	1
free neutral	0		
free charged	0		

D-H…A	D…A [Å]	D-H [Å]	H…A [Å]	D-H…A [º]	C1'…C1' [Å]	N_{all}	N_H	N_{uniq}
N2-H2…O6	2.859 (2)	1.015	1.919(1)	152.6 (4)		1	1	1
N7-H7…N1*	2.827 (4)	1.015	1.828(5)	167.48(7)				
C8-H8…O6	3.52 (1)	1.089	2.85 (2)	119 (1)				

GG_mHH_(67)(76)	Leontis-Westhof name	tHH (?)

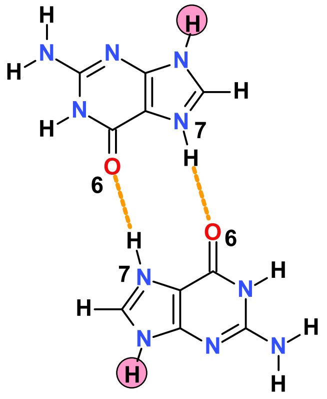

unique in CSD		7
in RNA Basepair Catalog		-

**Division**		**Protonation patterns**	
substituted neutral	0		
substituted charged	0		
free neutral	2	No R?	2
free charged	5	1H 3 7H 9H	5

D-H…A	D…A [Å]	D-H [Å]	H…A [Å]	D-H…A [º]	C1'…C1' [Å]	N_{all}	N_H	N_{uniq}
N7-H7… N6	2.8 (3)	1.015	1.71 (3)	162 (4)	-	10	8	7

**GG_mSS_(23)(32)**	**Leontis-Westhof name**	tSS
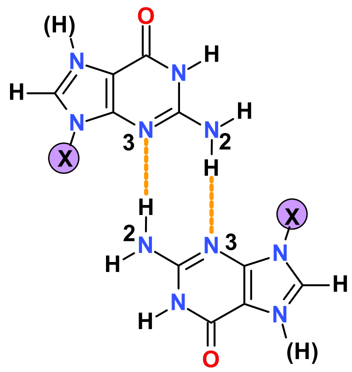	unique in CSD	33
	in RNA Basepair Catalog	49

**Division**		**Protonation patterns**	
substituted neutral	18	1H 3 7 9R	15
		R?	3
substituted charged	4	1H 3 7H R	4
free neutral	0		
free charged	11	1H 3 7H 9H	10
		1H 3 7 9H<br>1H 3 7H 9H	1

**D-H…A**	**D…A [Å]**	**D-H [Å]**	**H…A [Å]**	**D-H…A [º]**	**C1'…C1' [Å]**	**N_{all}**	**N_H**	**N_{uniq}**
N2-H2…N3	3.04 (6)	1.015	2.9 (8)	172 (7)	8.2 (2)	48	44	33

**GG_mSS_(39)(93)**	**Leontis-Westhof name**	tSS (?)
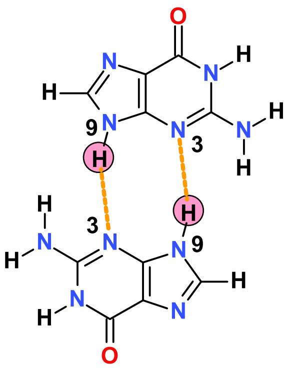	unique in CSD	1
	in RNA Basepair Catalog	-

**Division**		**Protonation patterns**	
substituted neutral	0		
substituted charged	0		
free neutral	1	1H 3 7 9H	1
free charged	0		

**D-H…A**	**D…A [Å]**	**D-H [Å]**	**H…A [Å]**	**D-H…A [º]**	**C1'…C1' [Å]**	**N_{all}**	**N_H**	**N_{uniq}**
N9-H9…N3	2.892	1.015	1.888	170	-	1	1	1

HYPOXANTHINE

HxHx_mWW_(16)(61)

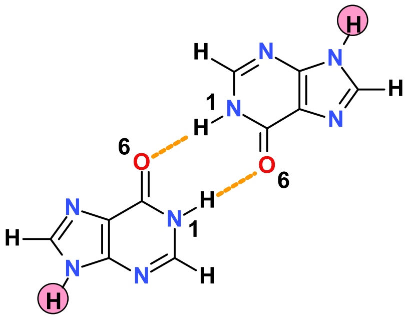

**Leontis-Westhof name**	tWW (?)
unique in CSD	1
in RNA Basepair Catalog	-

**Division**		**Protonation patterns**	
substituted neutral	0		
substituted charged	0		
free neutral	1	1H 3 7 9H	1
free charged	0		

**D-H…A**	**D…A [Å]**	**D-H [Å]**	**H…A [Å]**	**D-H…A [º]**	**C1'…C1' [Å]**	**N_{all}**	**N_H**	**N_{uniq}**
N1-H1…O6	2.785	1.015	1.778(4)	171 (3)	-	3	3	1

HxHx_fWH_(17)(26)

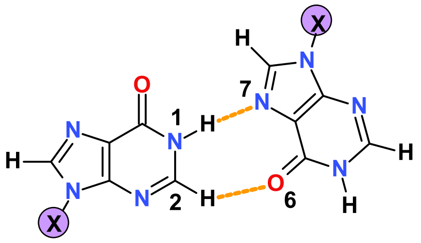

**Leontis-Westhof name**	tWH (?)
unique in CSD	9
in RNA Basepair Catalog	-

**Division**		**Protonation patterns**	
substituted neutral	7	1H 3 7 9R	7
substituted charged	0		
free neutral	2	No R?	2
free charged	0		

**D-H…A**	**D…A [Å]**	**D-H [Å]**	**H…A [Å]**	**D-H…A [º]**	**C1'…C1' [Å]**	**N_{all}**	**N_H**	**N_{uniq}**
N1-H1…N7	2.85 (7)	1.015	1.90 (8)	165 (8)	11.37 (9)	18	13	9
C2-H2…O6	3.28 (21)	1.089	2.39(19)	137 (15)				

HxHx_fWS_(13)(62)	Leontis-Westhof name		cWS (?)
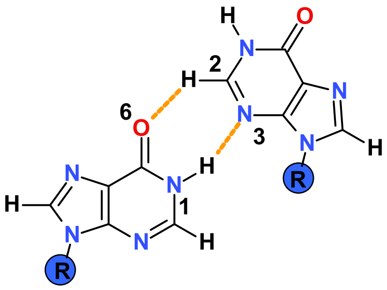	unique in CSD		1
	in RNA Basepair Catalog		-
	**Division**		**Protonation patterns**
	substituted neutral	1	1H 3 7 9R 1
	substituted charged	0	
	free neutral	0	
	free charged	0	

D-H…A	D…A [Å]	D-H [Å]	H…A [Å]	D-H…A [º]	C1'…C1' [Å]	N$_{all}$	N$_H$	N$_{uniq}$
N1-H1…N3	3.085	1.015	2.183	147	8.269	1	1	1
C2-H2…O6	3.145	1.089	2.313	132				

HxHx_fHS_(68)(79)	Leontis-Westhof name		cHS (?)
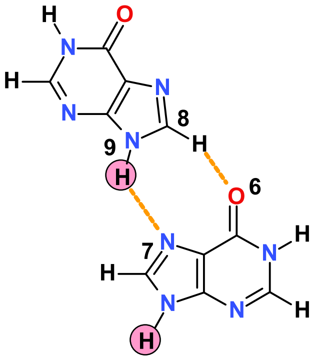	unique in CSD		1
	in RNA Basepair Catalog		-
	**Division**		**Protonation patterns**
	substituted neutral	0	
	substituted charged	0	
	free neutral	1	1H 3 7 9H 1
	free charged	0	

D-H…A	D…A [Å]	D-H [Å]	H…A [Å]	D-H…A [º]	C1'…C1' [Å]	N$_{all}$	N$_H$	N$_{uniq}$
C8-H8…O6	3.170(17)	1.089	2.33 (4)	133 (3)	-	3	3	1
N9-H9…N7	2.810 (8)	1.015	1.822(11)	164 (4)				

**HxHx_mSS_(23)(32)**	**Leontis-Westhof name**	tSS (?)
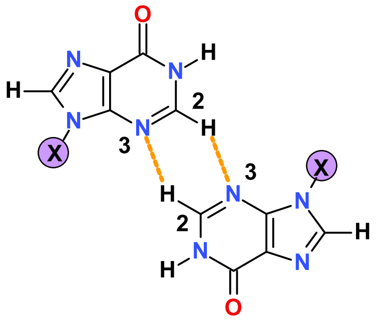	unique in CSD	2
	in RNA Basepair Catalog	-

**Division**		**Protonation patterns**	
substituted neutral	1	1H 3 7 9R	1
substituted charged	0		
free neutral	1	1H 3 7 9H	1
free charged	0		

**D-H…A**	**D…A [Å]**	**D-H [Å]**	**H…A [Å]**	**D-H…A [º]**	**C1’…C1’ [Å]**	**N_{all}**	**N_H**	**N_{uniq}**
C2-H2…N3	3.35 (4)	1.089	2.48 (6)	136 (4)	8.44 (1)	4	4	2

THYMINE

TT_mWW_(23)(32)	Leontis-Westhof name		tWW (?)
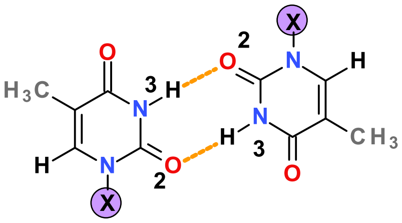	unique in CSD		66
	in RNA Basepair Catalog		-
	**Division**		**Protonation patterns**
	substituted neutral	60	1R 3H 52
			R? 8
	substituted charged	0	
	free neutral	6	1H 3H 6
	free charged	0	

D-H…A	D…A [Å]	D-H [Å]	H…A [Å]	D-H…A [º]	C1’…C1’ [Å]	N_{all}	N_H	N_{uniq}
N3-H3…O2	2.84 (5)	1.015	1.84 (5)	170 (6)	8.60 (15)	86	75	66

TT_fWW_(23)(34)	Leontis-Westhof name		cWW (?)
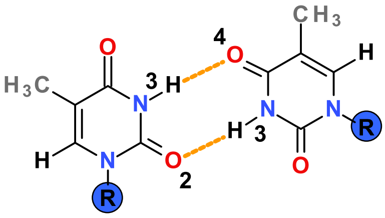	unique in CSD		6
	in RNA Basepair Catalog		-
	**Division**		**Protonation patterns**
	substituted neutral	6	1R 3H 6
	substituted charged	0	
	free neutral	0	
	free charged	0	

D-H…A	D…A [Å]	D-H [Å]	H…A [Å]	D-H…A [º]	C1’…C1’ [Å]	N_{all}	N_H	N_{uniq}
N3-H3…O2	2.84 (6)	1.015	1.86 (8)	170 (5)	8.49 (18)	6	6	6
N3-H3…O4	2.85 (6)	1.015	1.85 (7)	170 (4)				

TT_mWW_(34)(43)

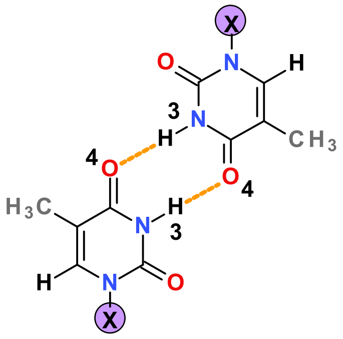

Leontis-Westhof name	tWW (?)
unique in CSD	59
in RNA Basepair Catalog	-

Division		Protonation patterns	
substituted neutral	58	1R 3H	57
		R?	1
substituted charged	0		
free neutral	0		
free charged	1	1 3H	1

D-H…A	D…A [Å]	D-H [Å]	H…A [Å]	D-H…A [º]	C1'…C1' [Å]	N_{all}	N_H	N_{uniq}
N3-H3…O4	2.87 (17)	1.015	1.84 (7)	171 (5)	11.04(14)	72	71	59

TT_fWH_(34)(45)

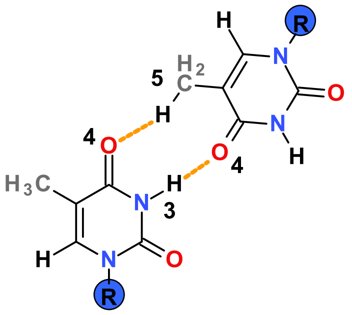

Leontis-Westhof name	tWH (?)
unique in CSD	1
in RNA Basepair Catalog	-

Division		Protonation patterns	
substituted neutral	1	1R 3H	1
substituted charged	0		
free neutral	0		
free charged	0		

D-H…A	D…A [Å]	D-H [Å]	H…A [Å]	D-H…A [º]	C1'…C1' [Å]	N_{all}	N_H	N_{uniq}
N3-H3…O4	3.001	1.015	2.005	167	11.56	1	1	1
C5-H5…O4	3.175	1.089	2.975	124				

**TT_fWS_(21)(32)**	**Leontis-Westhof name**	cWS (?)
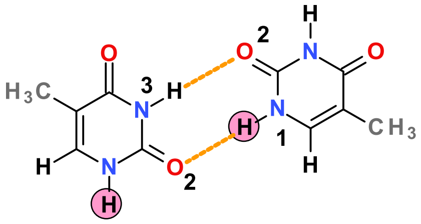	unique in CSD	5
	in RNA Basepair Catalog	-

**Division**		**Protonation patterns**	
substituted neutral	0		
substituted charged	0		
free neutral	5	1H 3H	4
		No R?	1
free charged	0		

**D-H…A**	**D…A [Å]**	**D-H [Å]**	**H…A [Å]**	**D-H…A [º]**	**C1'…C1' [Å]**	**N_{all}**	**N_H**	**N_{uniq}**
N1-H1…O2	2.83 (3)	1.015	1.84 (2)	171 (10)	-	6	5	5
N3-H3…O2	2.82 (1)	1.015	1.813 (9)	173 (3)				

**TT_mSS_(12)(21)**	**Leontis-Westhof name**	tSS (?)
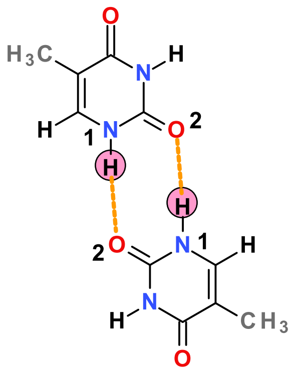	unique in CSD	5
	in RNA Basepair Catalog	-

**Division**		**Protonation patterns**	
substituted neutral	0		
substituted charged	0		
free neutral	5	1H 3H	5
free charged	0		

**D-H…A**	**D…A [Å]**	**D-H [Å]**	**H…A [Å]**	**D-H…A [º]**	**C1'…C1' [Å]**	**N_{all}**	**N_H**	**N_{uniq}**
N1-H1…O2	2.828(13)	1.015	1.823(16)	170 (2)	-	7	7	5

URACIL

UU_mWW_(23)(32)	Leontis-Westhof name	tWW (?)
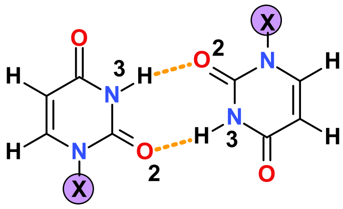	unique in CSD	19
	in RNA Basepair Catalog	-

Division		Protonation patterns	
substituted neutral	18	1R 3H	17
		R?	1
substituted charged	0		
free neutral	1	1H 3H	1
free charged	0		

D-H…A	D…A [Å]	D-H [Å]	H…A [Å]	D-H…A [º]	C1'…C1' [Å]	N_{all}	N_H	N_{uniq}
N3-H3…O2	2.85 (4)	1.015	1.86 (5)	167 (6)	8.58 (8)	21	20	19

UU_fWW_(23)(34)	Leontis-Westhof name	cWW
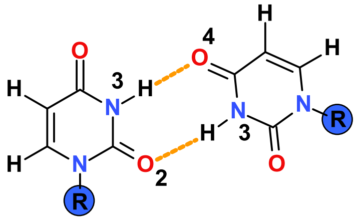	unique in CSD	4
	in RNA Basepair Catalog	432

Division		Protonation patterns	
substituted neutral	4	1R 3H	3
		R?	1
substituted charged	0		
free neutral	0		
free charged	0		

D-H…A	D…A [Å]	D-H [Å]	H…A [Å]	D-H…A [º]	C1'…C1' [Å]	N_{all}	N_H	N_{uniq}
N3-H3…O2	2.92 (11)	1.015	1.87 (6)	168 (6)	8.49 (11)	4	3	4
N3-H3…O4	2.89 (8)	1.015	1.85 (7)	164 (7)				

UU_mWW_(34)(43)

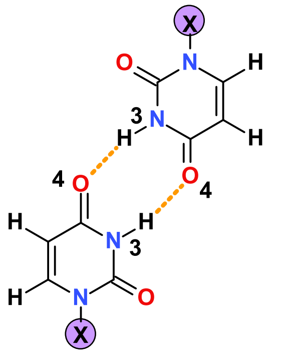

Leontis-Westhof name		tWW
unique in CSD		26
in RNA Basepair Catalog		5

**Division**		**Protonation patterns**	
substituted neutral	25	1R 3H	25
substituted charged	0		
free neutral	1	1H 3H	1
free charged	0		

**D-H…A**	**D…A [Å]**	**D-H [Å]**	**H…A [Å]**	**D-H…A [º]**	**C1'…C1' [Å]**	**N_{all}**	**N_H**	**N_{uniq}**
N3-H3…O4	2.84 (4)	1.015	1.84 (4)	170 (6)	11.04(15)	31	31	26

UU_fWH_(34)(45)

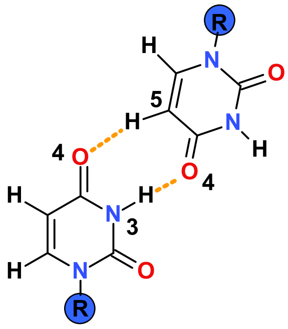

Leontis-Westhof name		tWH
unique in CSD		60
in RNA Basepair Catalog		16

**Division**		**Protonation patterns**	
substituted neutral	60	1R 3H	53
		R?	7
substituted charged	0		
free neutral	0		
free charged	0		

**D-H…A**	**D…A [Å]**	**D-H [Å]**	**H…A [Å]**	**D-H…A [º]**	**C1'…C1' [Å]**	**N_{all}**	**N_H**	**N_{uniq}**
N3-H3…O4	2.81 (5)	1.015	1.83 (7)	143 (9)	11.32(17)	72	63	60
C5-H5…O4	3.32 (12)	1.089	2.39 (16)	168 (7)				

**UU_mHH_(45)(54)**	**Leontis-Westhof name**			tHH (?)
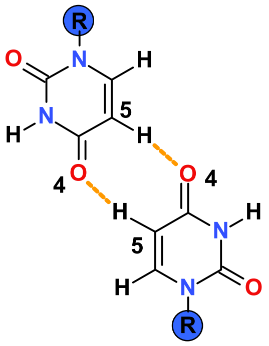	unique in CSD			9
	in RNA Basepair Catalog			-
	**Division**		**Protonation patterns**	
	substituted neutral	9	1R 3H	9
	substituted charged	0		
	free neutral	0		
	free charged	0		

**D-H…A**	**D…A [Å]**	**D-H [Å]**	**H…A [Å]**	**D-H…A [º]**	**C1’…C1’ [Å]**	**N_{all}**	**N_H**	**N_{uniq}**
C5-H5…O4	3.5 (3)	1.089	2.5 (3)	153 (18)	11.5 (4)	9	9	9

**UU_mHS_(41)(52)**	**Leontis-Westhof name**			cHS (?)
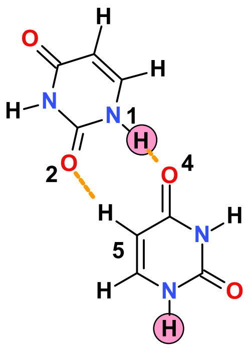	unique in CSD			1
	in RNA Basepair Catalog			-
	**Division**		**Protonation patterns**	
	substituted neutral	0		
	substituted charged	0		
	free neutral	1	1H 3H	1
	free charged	0		

**D-H…A**	**D…A [Å]**	**D-H [Å]**	**H…A [Å]**	**D-H…A [º]**	**C1’…C1’ [Å]**	**N_{all}**	**N_H**	**N_{uniq}**
N1-H1…O4	2.864	1.015	1.858	171	-	1	1	1
C5-H5…O2	3.320	1.089	2.268	161.71				

UU_mSS_(12)(21)	**Leontis-Westhof name**	tSS (?)
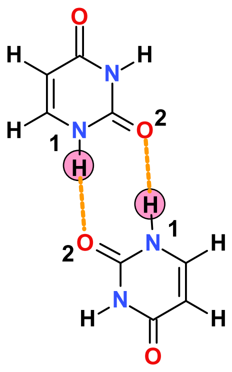	unique in CSD	1
	in RNA Basepair Catalog	-

**Division**		**Protonation patterns**	
substituted neutral	0		
substituted charged	0		
free neutral	1	1H 3H	1
free charged	0		

**D-H…A**	**D…A [Å]**	**D-H [Å]**	**H…A [Å]**	**D-H…A [º]**	**C1'…C1' [Å]**	**N_{all}**	**N_H**	**N_{uniq}**
N1-H1…O2	2.845	1.015	1.844	168	-	1	1	1

CYTOSINE

**CC_mWW_(23)(32)**	**Leontis-Westhof name**			tWW (?)
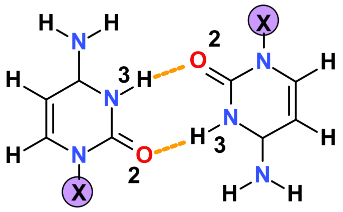	unique in CSD			2
	in RNA Basepair Catalog			-
	**Division**		**Protonation patterns**	
	substituted neutral	0		
	substituted charged	1	1R 3H	1
	free neutral	0		
	free charged	1	1H 3H	1

**D-H…A**	**D…A [Å]**	**D-H [Å]**	**H…A [Å]**	**D-H…A [º]**	**C1'…C1' [Å]**	N_{all}	N_{H}	N_{uniq}
N3-H3…O2	2.77 (3)	1.015	1.78 (4)	163 (3)	8.526	2	2	2

**CC_mWW_(24)(33)(42)**	**Leontis-Westhof name**			tWW
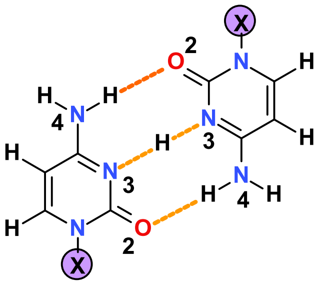	unique in CSD			62
	in RNA Basepair Catalog			7
	**Division**		**Protonation patterns**	
	substituted neutral	7	1R 3	3
			R?	4
	substituted charged	15	1R 3<br>1R 3H	15
	free neutral	4	1H 3	3
			No R?	1
	free charged	36	1H 3<br>1H 3H	36

**D-H…A**	**D…A [Å]**	**D-H [Å]**	**H…A [Å]**	**D-H…A [º]**	**C1'…C1' [Å]**	N_{all}	N_{H}	N_{uniq}
N4-H4…O2	2.84 (5)	1.015	1.84 (7)	172 (9)	9.53 (9)	105	96	62
N3-H3…N3	2.82 (3)							

<table>
<tr><td colspan="2">CC_mWW_(34)(43)</td><td colspan="4">Leontis-Westhof name</td><td>tWW (?)</td></tr>
<tr><td colspan="2" rowspan="4">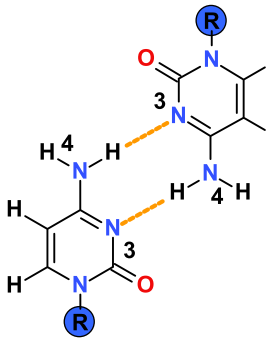</td><td colspan="4">unique in CSD
in RNA Basepair Catalog</td><td>24
-</td></tr>
<tr><td colspan="3">Division</td><td colspan="2">Protonation patterns</td></tr>
<tr><td colspan="2">substituted neutral
substituted charged
free neutral
free charged</td><td>24
0
0
0</td><td>1R 3</td><td>24</td></tr>
</table>

D-H…A	D…A [Å]	D-H [Å]	H…A [Å]	D-H…A [º]	C1'…C1' [Å]	N_{all}	N_H	N_{uniq}
N4-H4…N3	3.00 (4)	1.015	2.01 (5)	169 (8)	10.96(17)	27	26	24

<table>
<tr><td colspan="2">CC_fWH_(24)(35)</td><td colspan="4">Leontis-Westhof name</td><td>tWH</td></tr>
<tr><td colspan="2" rowspan="4">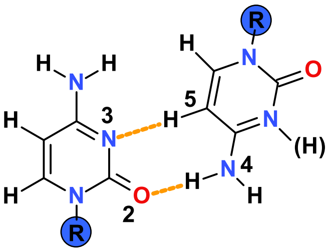</td><td colspan="4">unique in CSD
in RNA Basepair Catalog</td><td>9
8</td></tr>
<tr><td colspan="3">Division</td><td colspan="2">Protonation patterns</td></tr>
<tr><td colspan="2">substituted neutral
substituted charged

free neutral
free charged</td><td>8
1

0
0</td><td>1R 3
1R 3
1R 3H</td><td>8
1</td></tr>
</table>

D-H…A	D…A [Å]	D-H [Å]	H…A [Å]	D-H…A [º]	C1'…C1' [Å]	N_{all}	N_H	N_{uniq}
N4-H4…O2	3.0 (2)	1.015	2.8 (3)	151 (18)	11.0 (2)	11	11	9
C5-H5…N3	3.8 (3)	1.089	2.2 (4)	149 (12)				

**CC_mWH_(25)(34)**	**Leontis-Westhof name**	cWH		
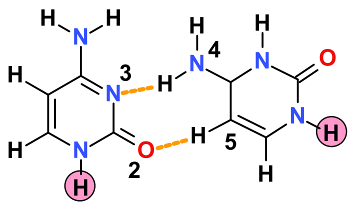	unique in CSD	1		
	in RNA Basepair Catalog	4		
	**Division**		**Protonation patterns**	
	substituted neutral	0		
	substituted charged	1	1R 3<br>1R 3H	1
	free neutral	0		
	free charged	0		

D-H…A	D…A [Å]	D-H [Å]	H…A [Å]	D-H…A [º]	C1'…C1' [Å]	N_{all}	N_H	N_{uniq}
C5-H5…O2	3.489	1.089	2.448	159	8.996	1	1	1
N4-H4…N3	3.017	1.015	2.006	174				

**CC_fWS_(26)(31)(42)**	**Leontis-Westhof name**	tWS (?)		
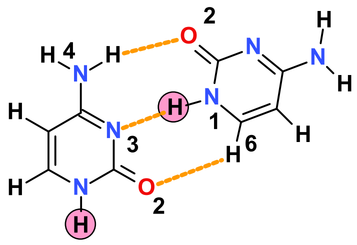	unique in CSD	3		
	in RNA Basepair Catalog	-		
	**Division**		**Protonation patterns**	
	substituted neutral	0		
	substituted charged	0		
	free neutral	3	1H 3	3
	free charged	0		

D-H…A	D…A [Å]	D-H [Å]	H…A [Å]	D-H…A [º]	C1'…C1' [Å]	N_{all}	N_H	N_{uniq}
C6-H6…O2	3.7 (2)	1.089	2.95 (16)	127 (3)	-	14	14	3
N1-H1…N3	2.89 (7)	1.015	1.88 (6)	172 (7)				
N4-H4…O2	3.00 (5)	1.015	2.00 (5)	172 (4)				

**CC_mSS_(12)(21)**	**Leontis-Westhof name**			tSS (?)
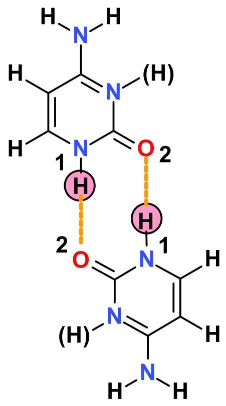	unique in CSD			18
	in RNA Basepair Catalog			-
	**Division**		**Protonation patterns**	
	substituted neutral	0		
	substituted charged	0		
	free neutral	6	1H 3	5
			No R?	1
	free charged	12	1H 3H	10
			1H 3<br>1H 3H	2

**D-H…A**	**D…A [Å]**	**D-H [Å]**	**H…A [Å]**	**D-H…A [º]**	**C1'…C1' [Å]**	**N_{all}**	**N_H**	**N_{uniq}**
N1-H1…O2	2.82 (2)	1.015	1.81 (3)	173 (4)	-	26	24	18
